# Supplementary material for: IRAK1-dependent Regnase-1-14-3-3 complex formation controls Regnase-1-mediated mRNA decay
Source: eLife. 2021 Oct 12;10:e71966. doi: 10.7554/eLife.71966 (PMC8553338; doi:10.7554/eLife.71966)

Figure 1—figure supplement 1

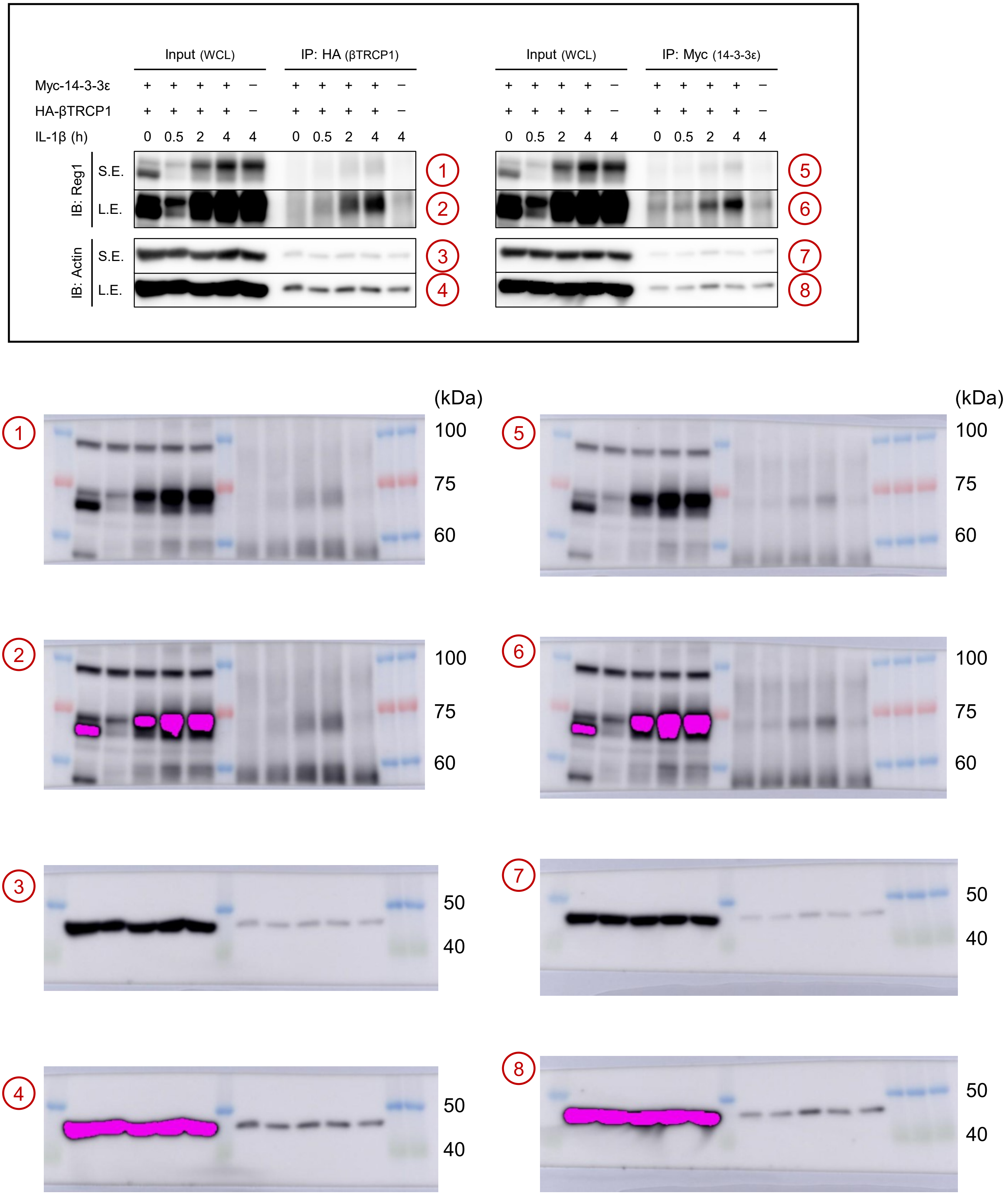

Figure 1—figure supplement 2

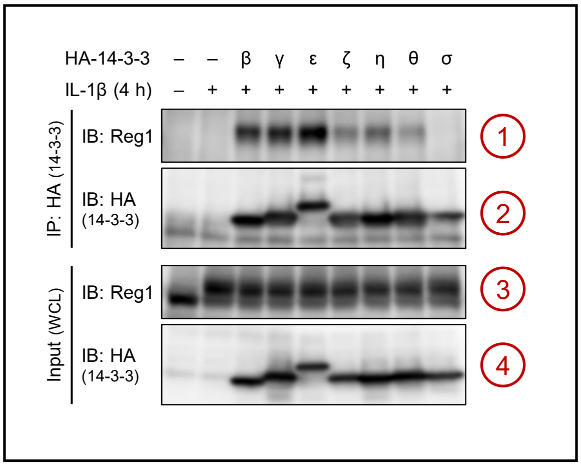

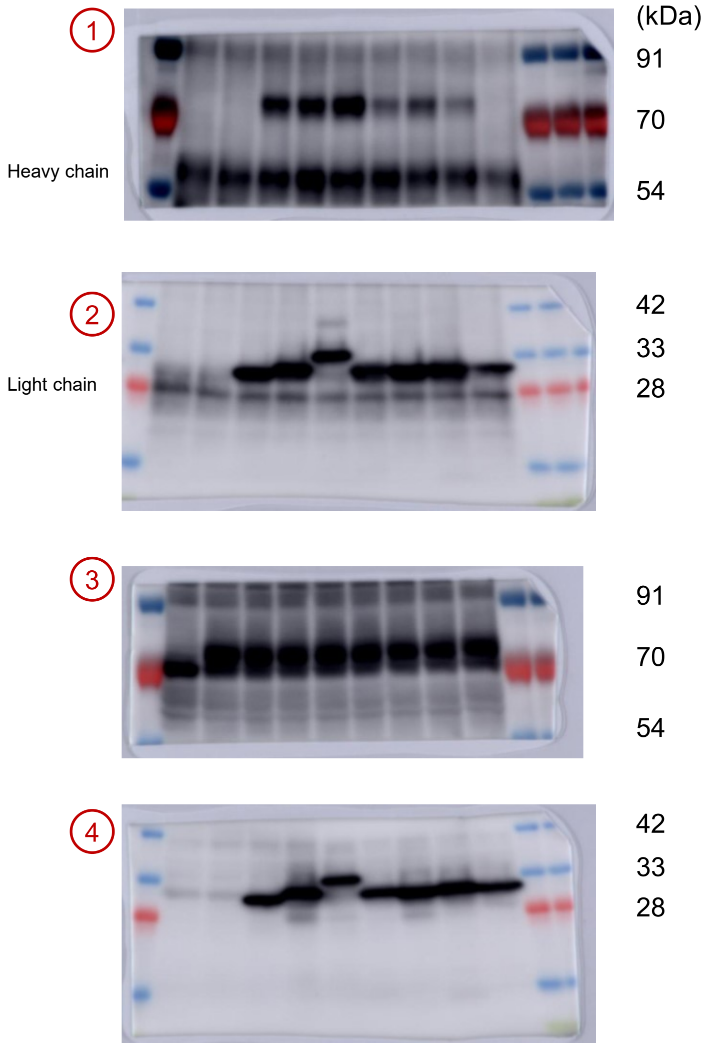

Figure 2—figure supplement 1

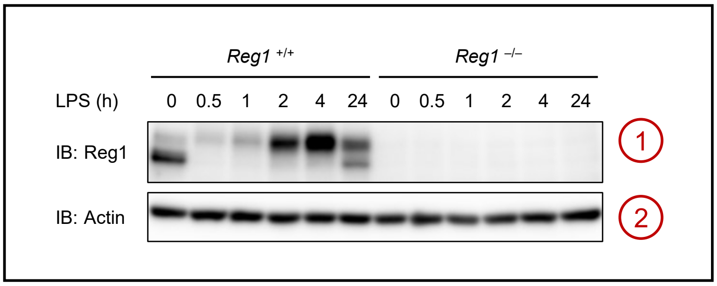

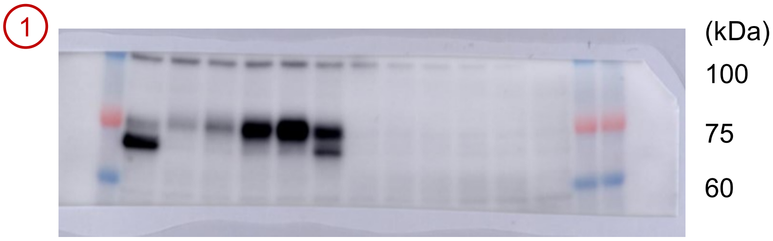

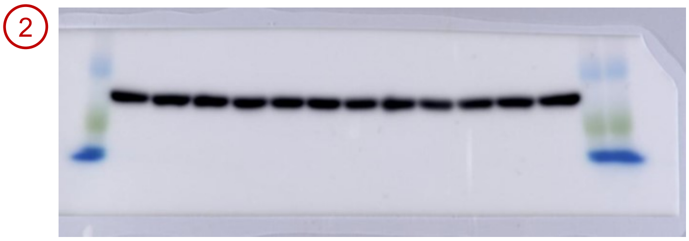

Figure 2—figure supplement 3

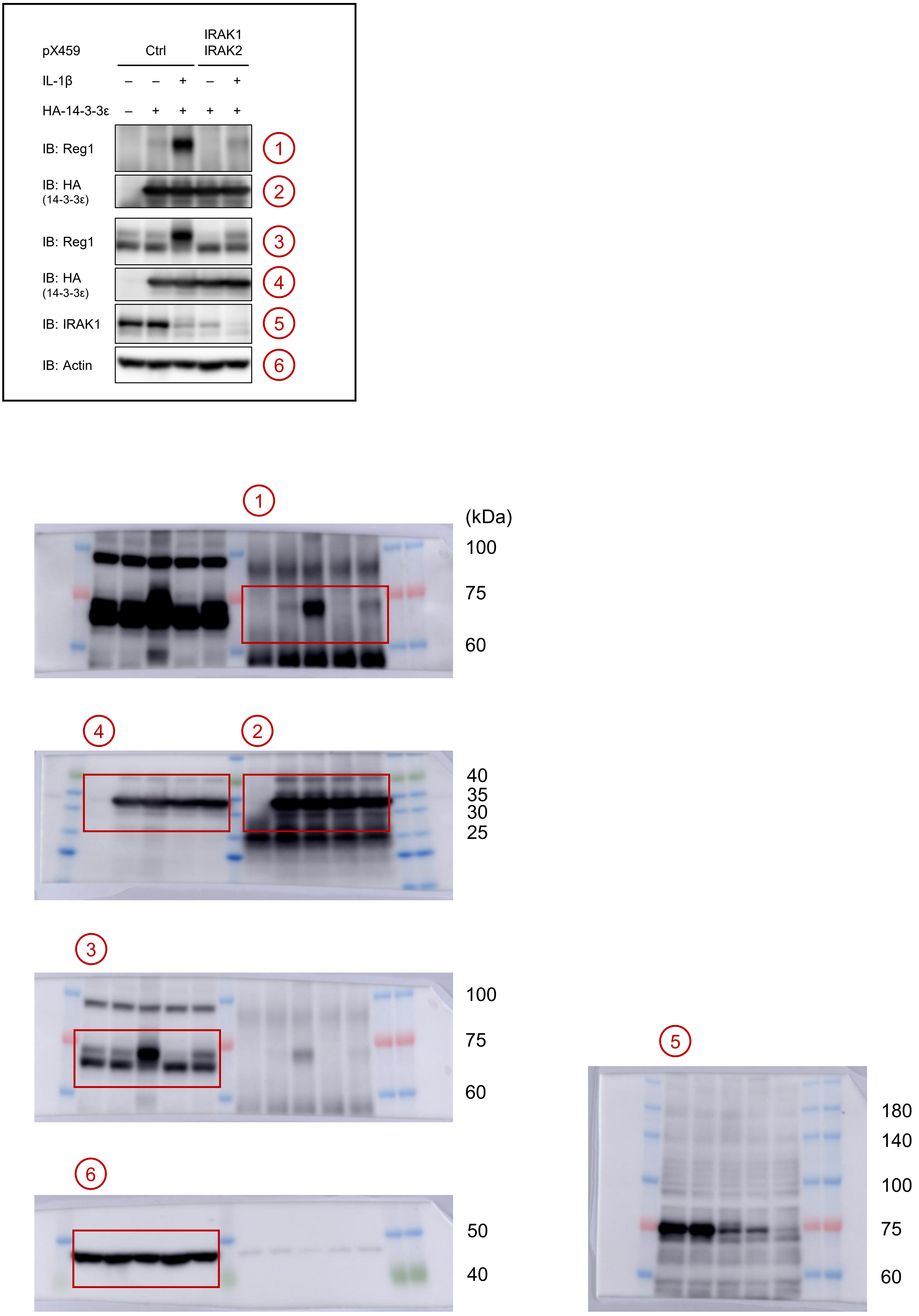

Figure 2—figure supplement 8

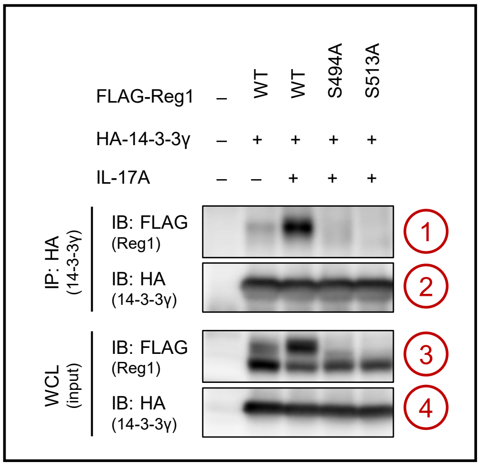

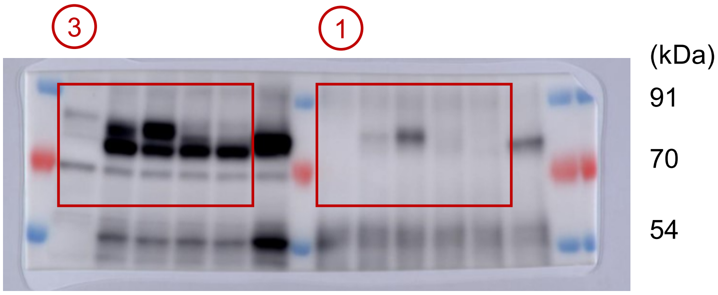

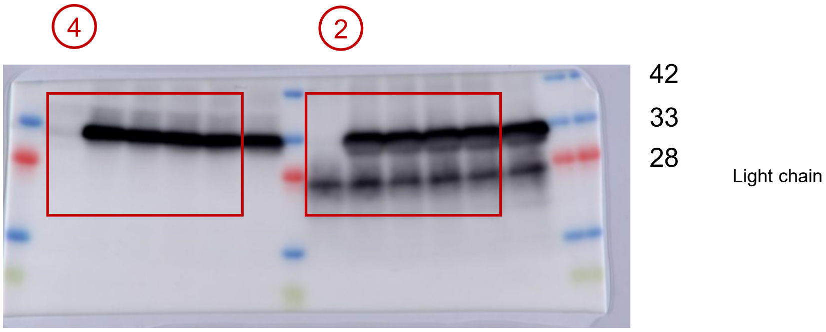

Figure 4—figure supplement 2

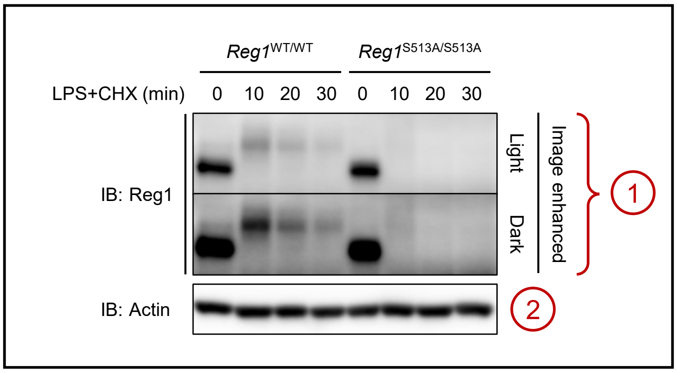

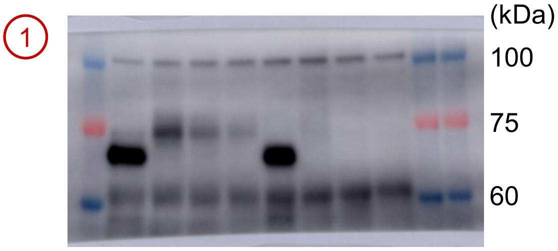

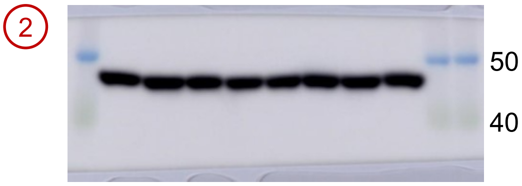

Figure 5—figure supplement 1

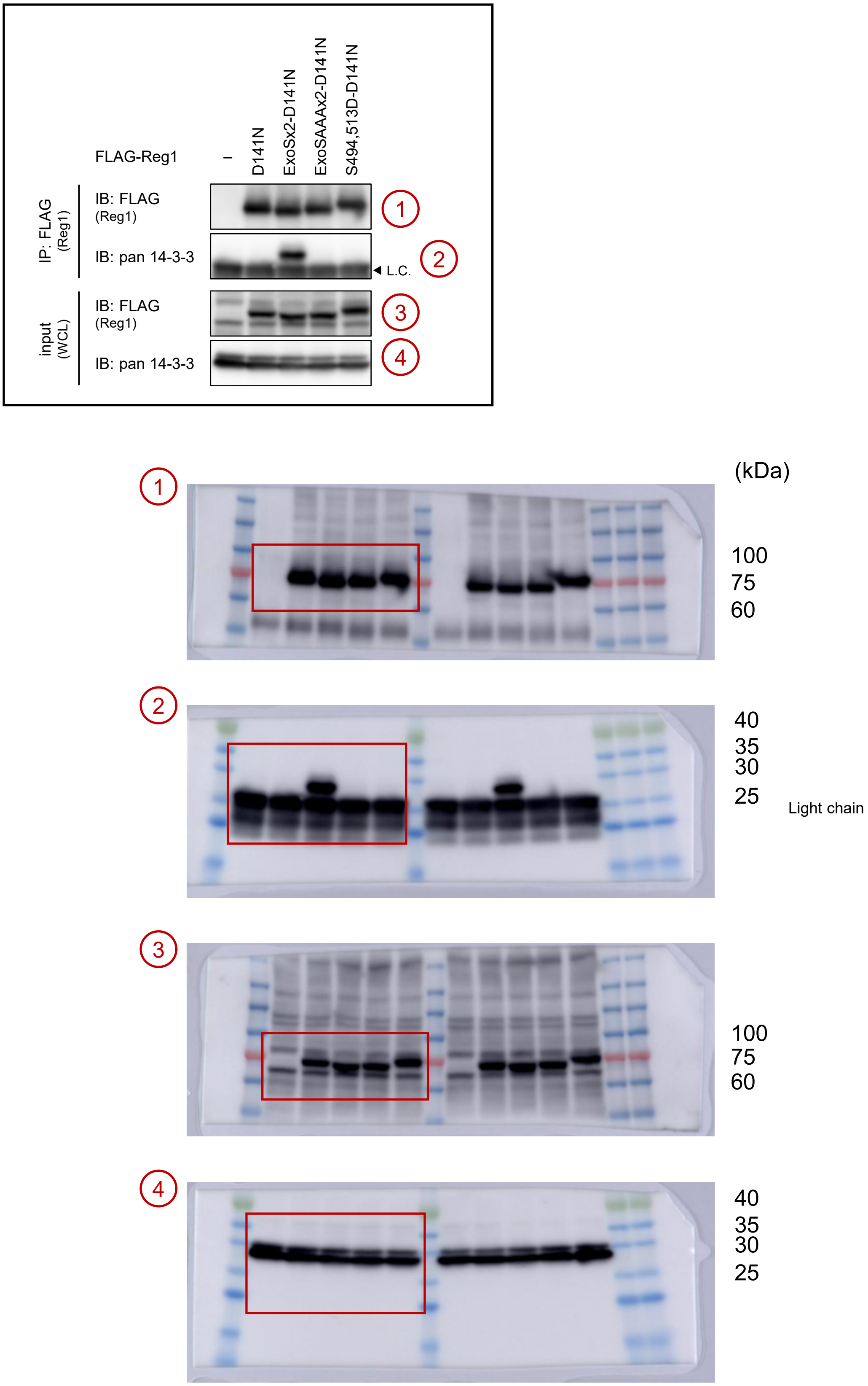

Supplement: Source data 1. [file elife-71966-data1.zip › Source Data Files/Blots (Figure Supplement).pdf]
