# Supplementary material for: IRAK1-dependent Regnase-1-14-3-3 complex formation controls Regnase-1-mediated mRNA decay
Source: eLife. 2021 Oct 12;10:e71966. doi: 10.7554/eLife.71966 (PMC8553338; doi:10.7554/eLife.71966)
Supplement: Source data 1. [file elife-71966-data1.zip › Source Data Files/Blots (Main Figure).pdf]

Figure 1-C

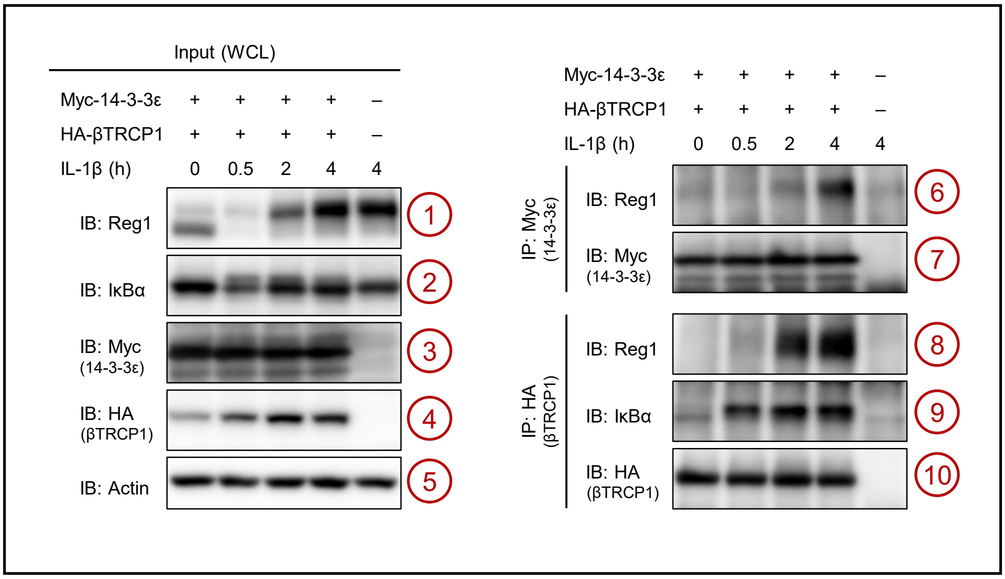
Input (WCL)
Myc-14-3-3ε + + + + –
HA-βTRCP1 + + + + –
IL-1β (h) 0 0.5 2 4 4
IB: Reg1
IB: IκBα
IB: Myc (14-3-3ε)
IB: HA (βTRCP1)
IB: Actin
1
2
3
4
5
Myc-14-3-3ε + + + + –
HA-βTRCP1 + + + + –
IL-1β (h) 0 0.5 2 4 4
IP: Myc (14-3-3ε)
IB: Reg1
IB: Myc (14-3-3ε)
IP: HA (βTRCP1)
IB: Reg1
IB: IκBα
IB: HA (βTRCP1)
6
7
8
9
10

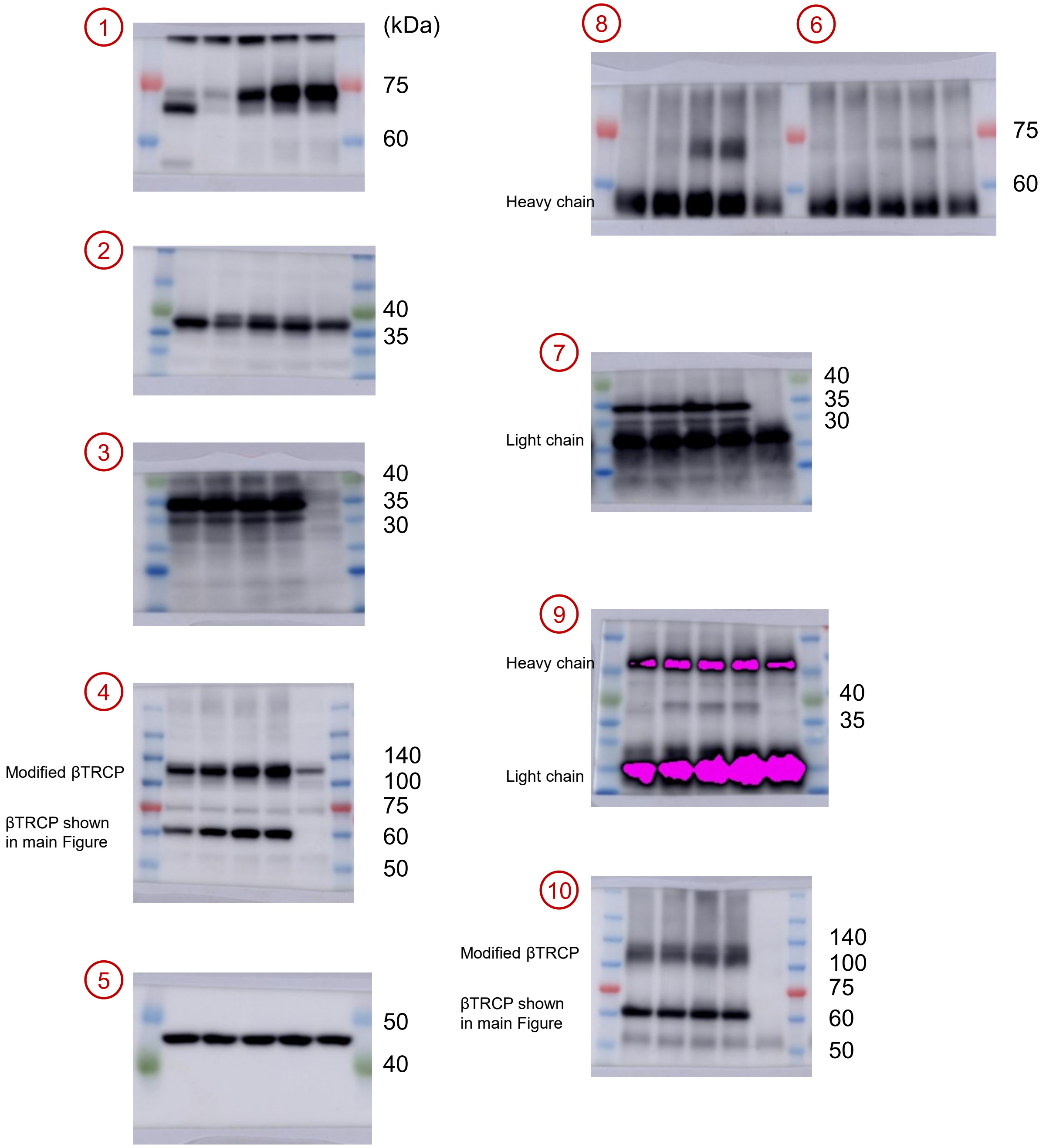
1
(kDa)
75
60
2
40
35
3
40
35
30
4
Modified βTRCP
βTRCP shown in main Figure
140
100
75
60
50
5
50
40
8
6
Heavy chain
75
60
7
Light chain
40
35
30
9
Heavy chain
Light chain
40
35
10
Modified βTRCP
βTRCP shown in main Figure
140
100
75
60
50

Figure 1-D

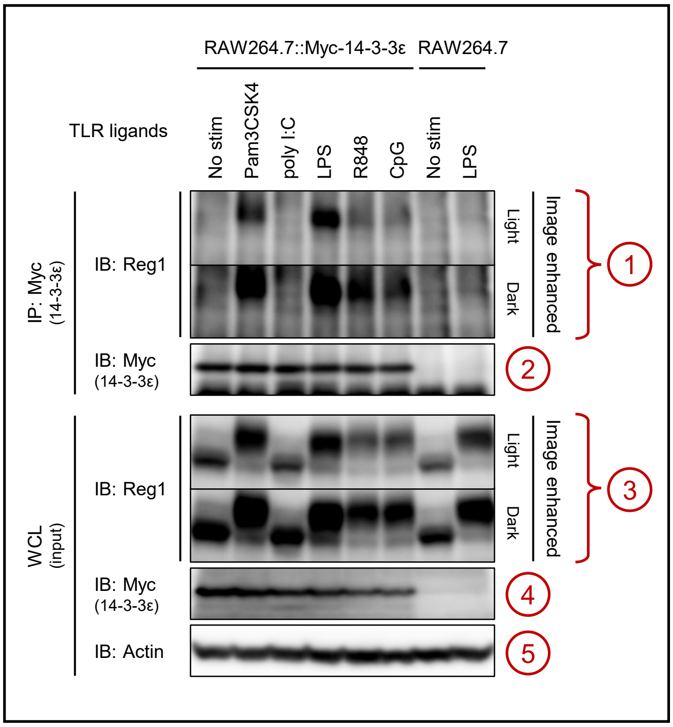

RAW264.7::Myc-14-3-3ε
RAW264.7
TLR ligands
No stim
Pam3CSK4
poly I:C
LPS
R848
CpG
No stim
LPS
IP: Myc (14-3-3ε)
IB: Reg1
IB: Myc (14-3-3ε)
WCL (input)
IB: Reg1
IB: Myc (14-3-3ε)
IB: Actin
Light
Dark
Image enhanced
1
2
3
4
5

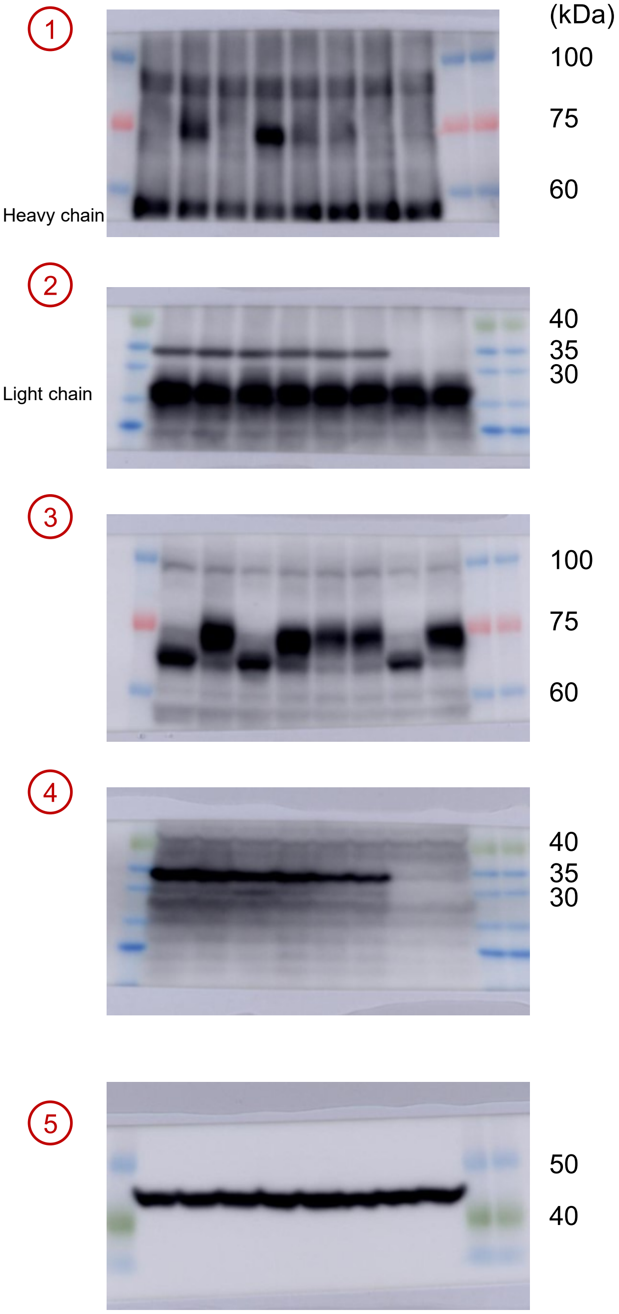

1
(kDa)
100
75
60
Heavy chain
2
40
35
30
Light chain
3
100
75
60
4
40
35
30
5
50
40

Figure 2-A

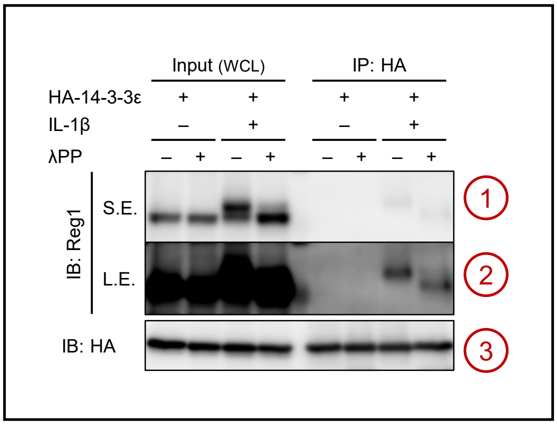
Input (WCL)
IP: HA
HA-14-3-3ε
IL-1β
λPP
IB: Reg1
S.E.
L.E.
IB: HA
1
2
3

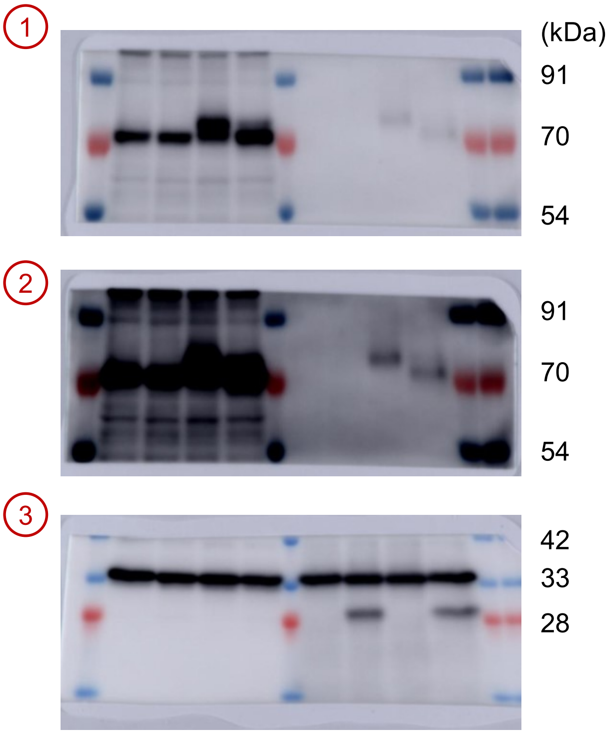
1
2
3
(kDa)
91
70
54
91
70
54
42
33
28

Figure 2-D

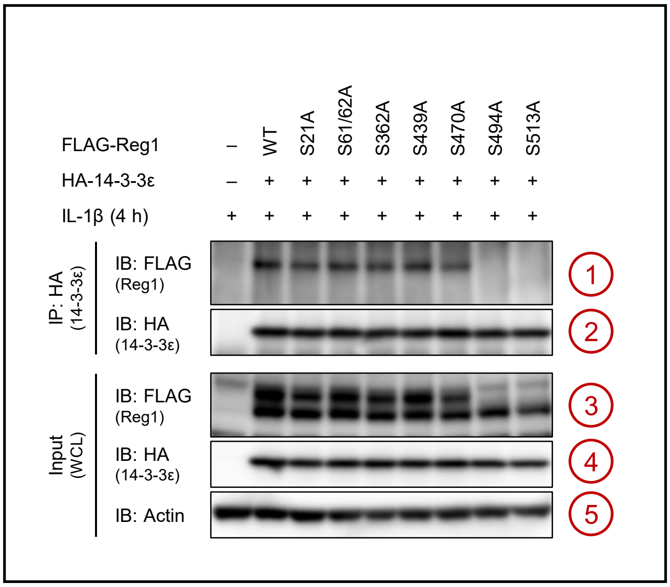
FLAG-Reg1
–
WT
S21A
S61/62A
S362A
S439A
S470A
S494A
S513A
HA-14-3-3ε
–
+
IL-1β (4 h)
+
IP: HA
(14-3-3ε)
IB: FLAG
(Reg1)
IB: HA
(14-3-3ε)
Input
(WCL)
IB: FLAG
(Reg1)
IB: HA
(14-3-3ε)
IB: Actin
1
2
3
4
5

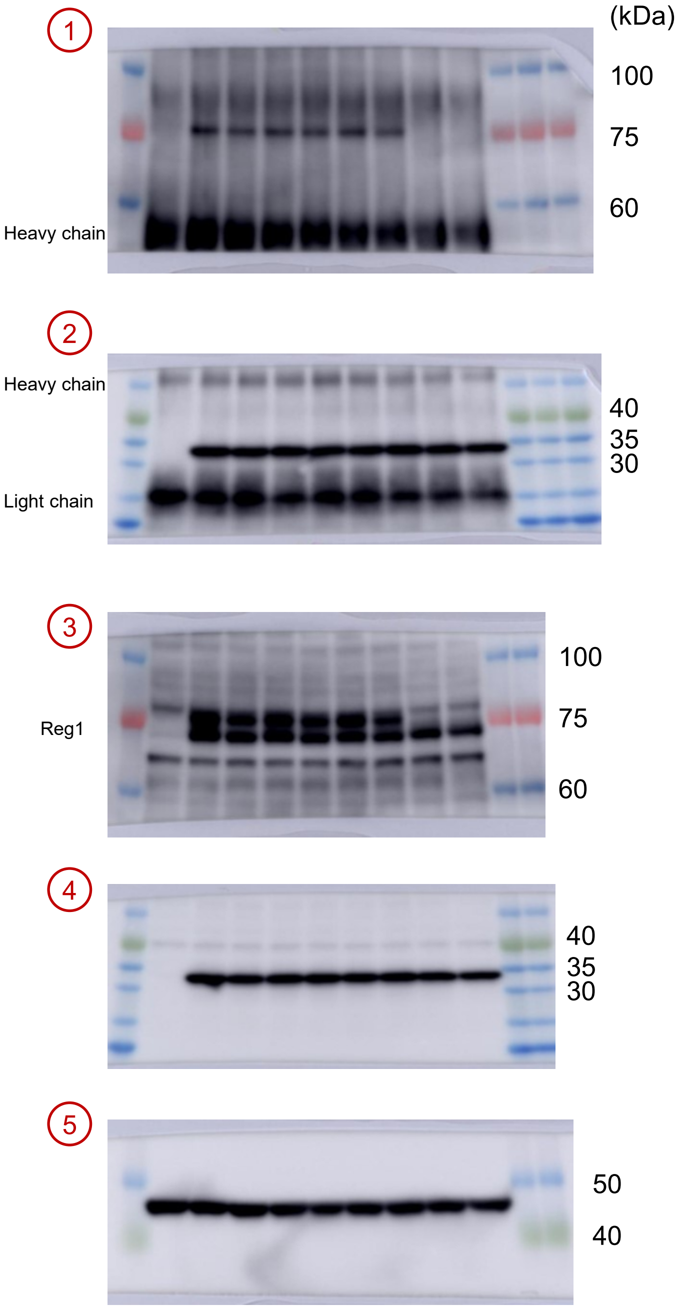
1
(kDa)
100
75
60
Heavy chain
2
Heavy chain
Light chain
40
35
30
3
Reg1
100
75
60
4
40
35
30
5
50
40

Figure 2-G

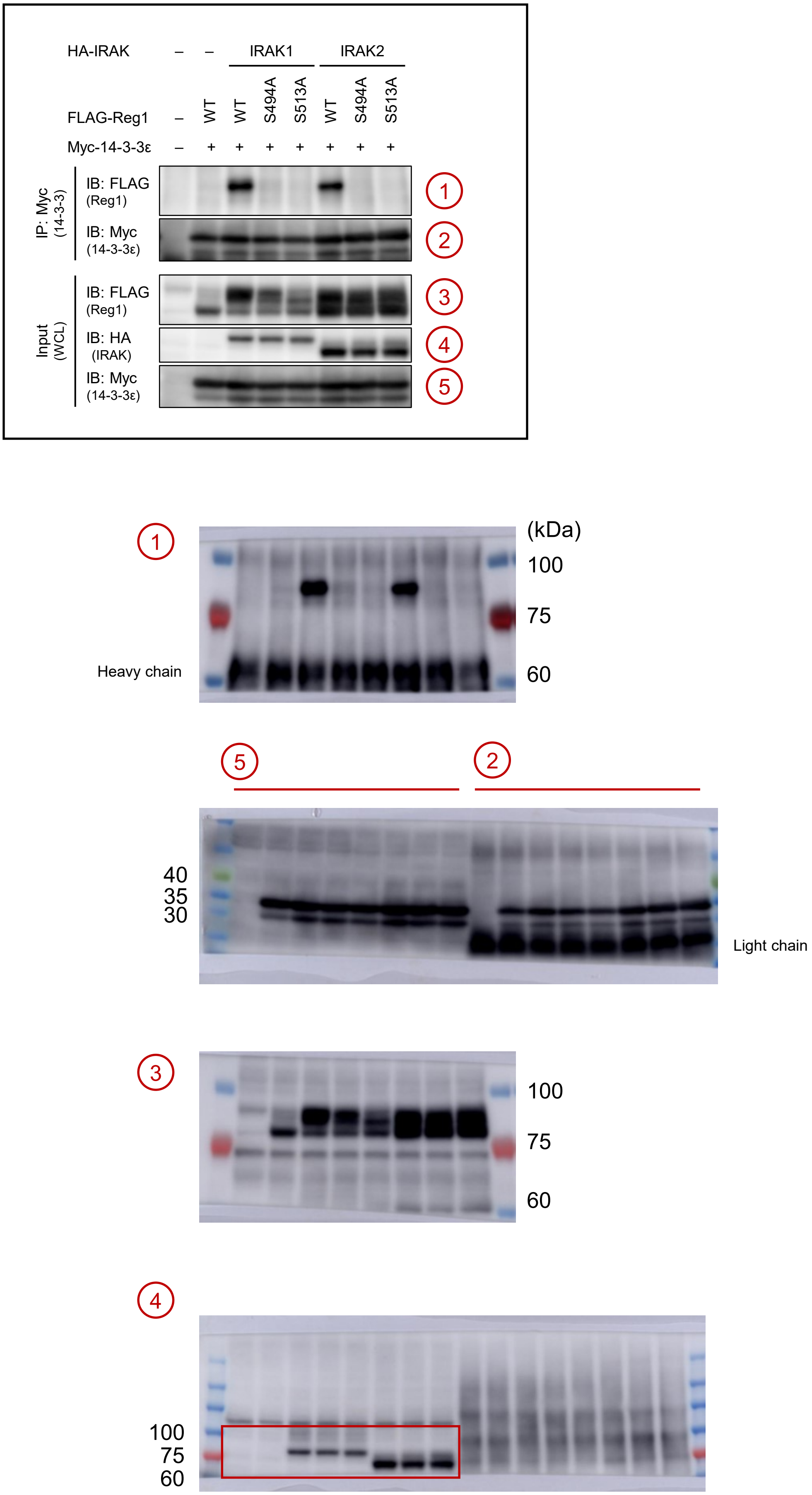

HA-IRAK – – IRAK1 IRAK2
FLAG-Reg1 – WT WT S494A S513A WT S494A S513A
Myc-14-3-3ε – + + + + + + +
IP: Myc (14-3-3)
IB: FLAG (Reg1)
IB: Myc (14-3-3ε)
Input (WCL)
IB: FLAG (Reg1)
IB: HA (IRAK)
IB: Myc (14-3-3ε)
1
2
3
4
5
(kDa)
100
75
60
Heavy chain
40
35
30
Light chain

Figure 2-I

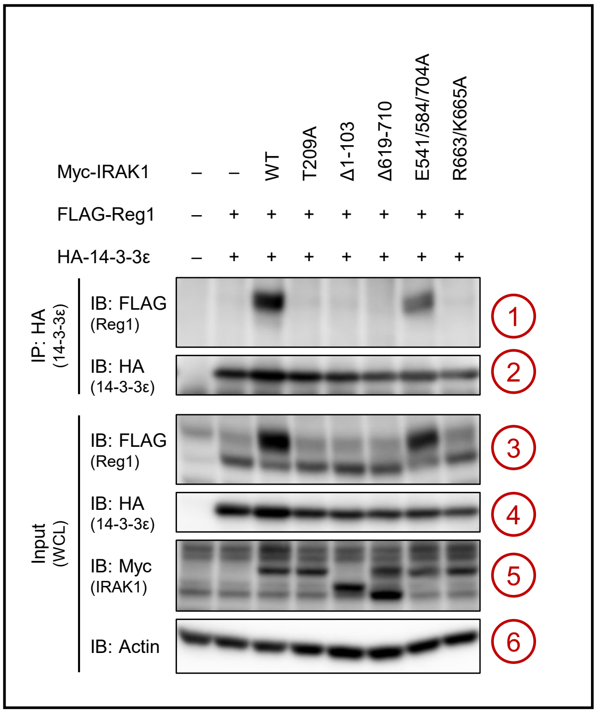

Myc-IRAK1
–
–
WT
T209A
Δ1-103
Δ619-710
E541/584/704A
R663/K665A
FLAG-Reg1
HA-14-3-3ε
IP: HA (14-3-3ε)
IB: FLAG (Reg1)
IB: HA (14-3-3ε)
Input (WCL)
IB: FLAG (Reg1)
IB: HA (14-3-3ε)
IB: Myc (IRAK1)
IB: Actin
1
2
3
4
5
6

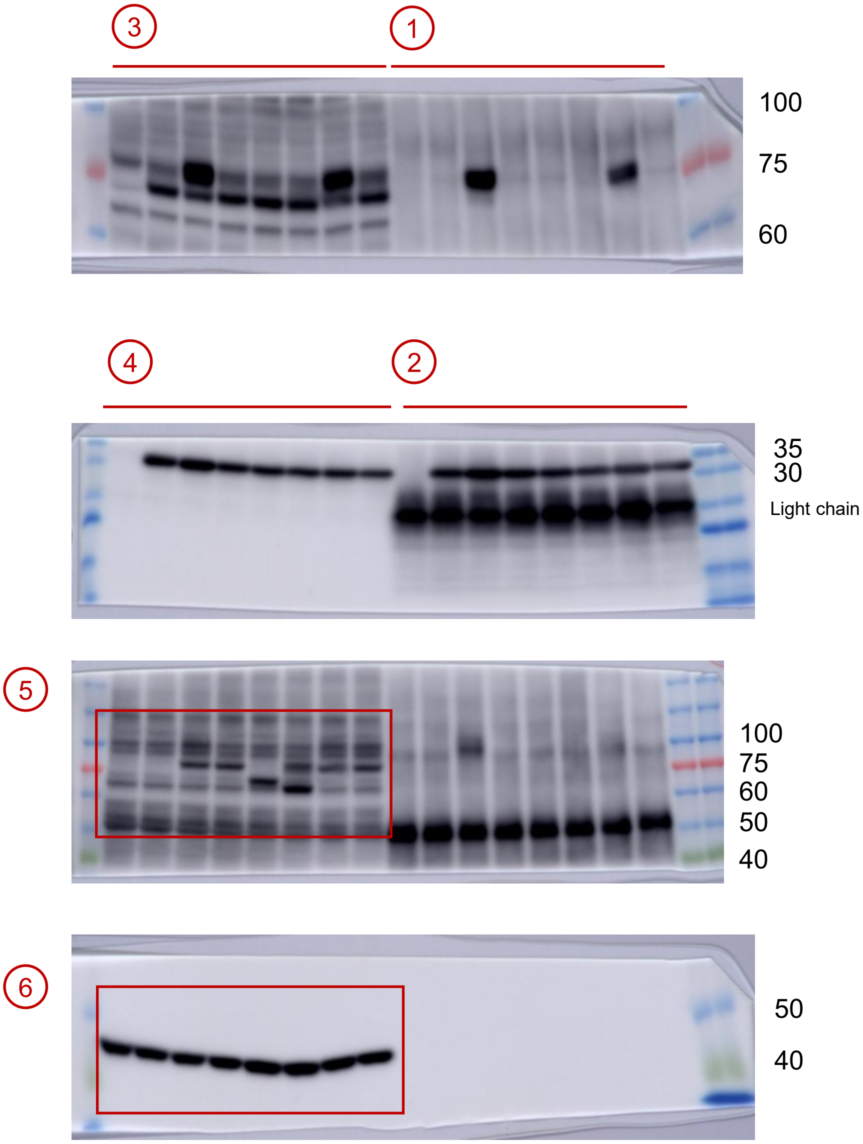

3
1
100
75
60
4
2
35
30
Light chain
5
100
75
60
50
40
6
50
40

Figure 3-A

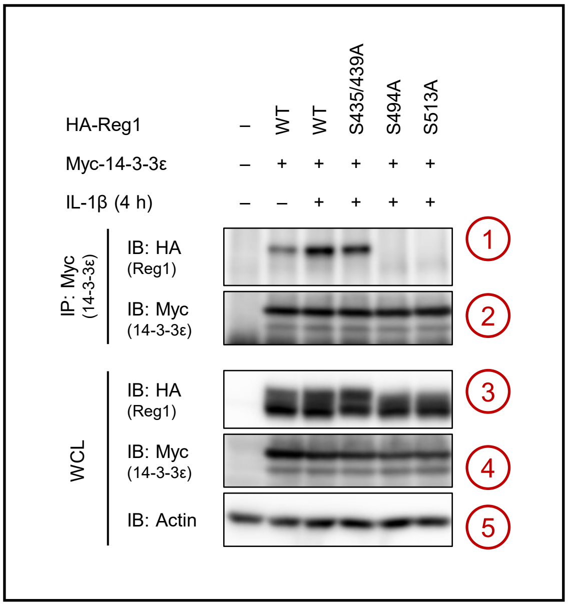
HA-Reg1
–
WT
WT
S435/439A
S494A
S513A
Myc-14-3-3ε
– + + + + +
IL-1β (4 h)
– – + + + +
IP: Myc
(14-3-3ε)
IB: HA
(Reg1)
1
IB: Myc
(14-3-3ε)
2
WCL
IB: HA
(Reg1)
3
IB: Myc
(14-3-3ε)
4
IB: Actin
5

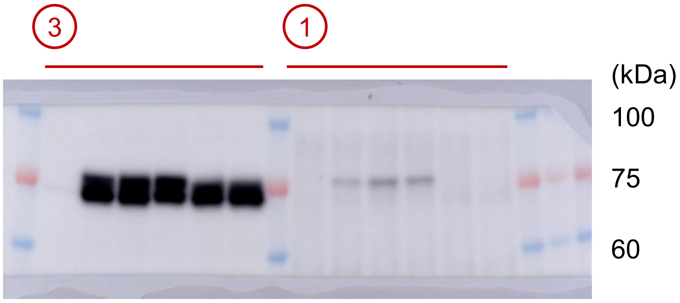
3
1
(kDa)
100
75
60

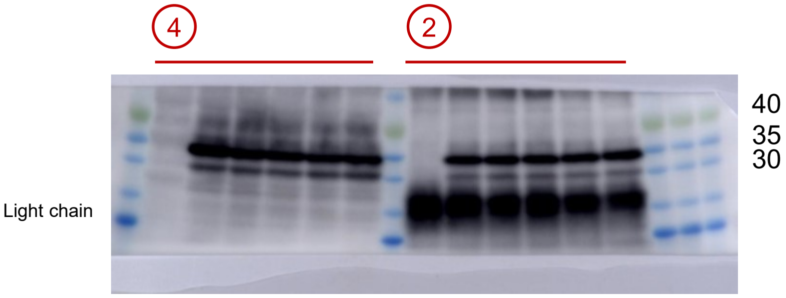
4
2
Light chain
40
35
30

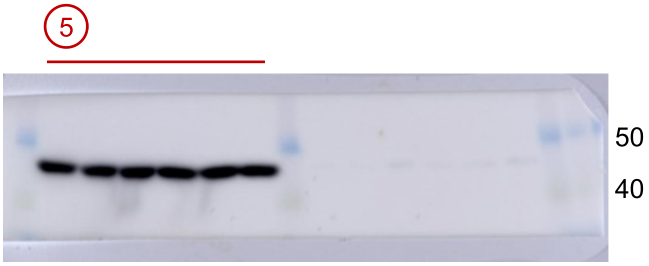
5
50
40

Figure 3-B

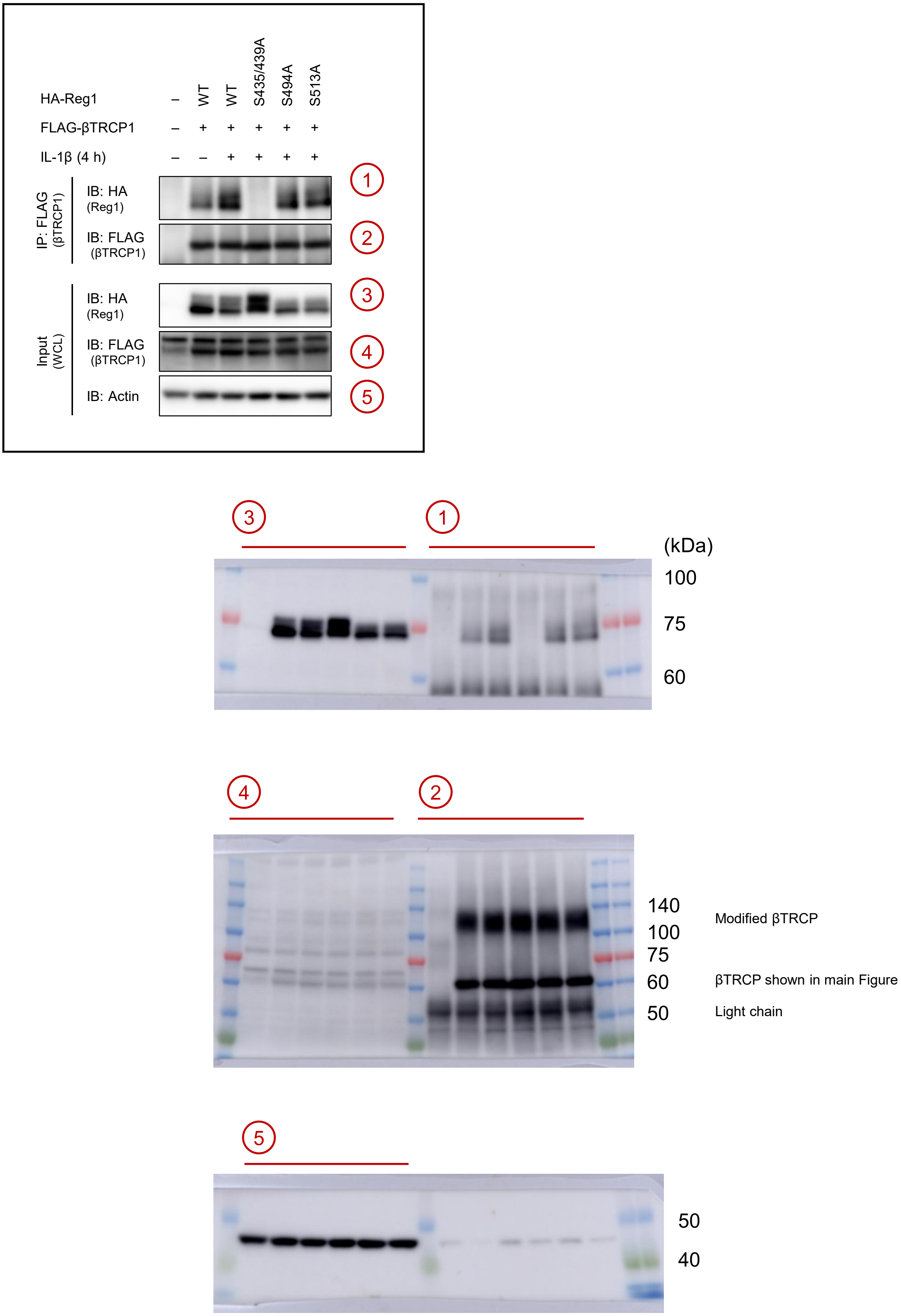
HA-Reg1 – WT WT S435/439A S494A S513A
FLAG-βTRCP1 – + + + + +
IL-1β (4 h) – – + + + +
IP: FLAG (βTRCP1)
IB: HA (Reg1)
IB: FLAG (βTRCP1)
Input (WCL)
IB: HA (Reg1)
IB: FLAG (βTRCP1)
IB: Actin
1
2
3
4
5
(kDa)
100
75
60
140
50
40
Modified βTRCP
βTRCP shown in main Figure
Light chain

Figure 3-C

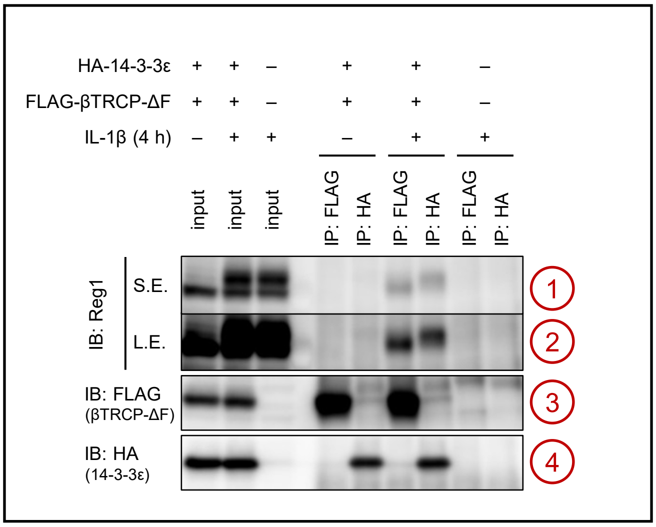
HA-14-3-3ε + + – + + –
FLAG-βTRCP-ΔF + + – + + –
IL-1β (4 h) – + + – + +
input
input
input
IP: FLAG
IP: HA
IP: FLAG
IP: HA
IP: FLAG
IP: HA
IB: Reg1
S.E.
L.E.
IB: FLAG
(βTRCP-ΔF)
IB: HA
(14-3-3ε)
1
2
3
4

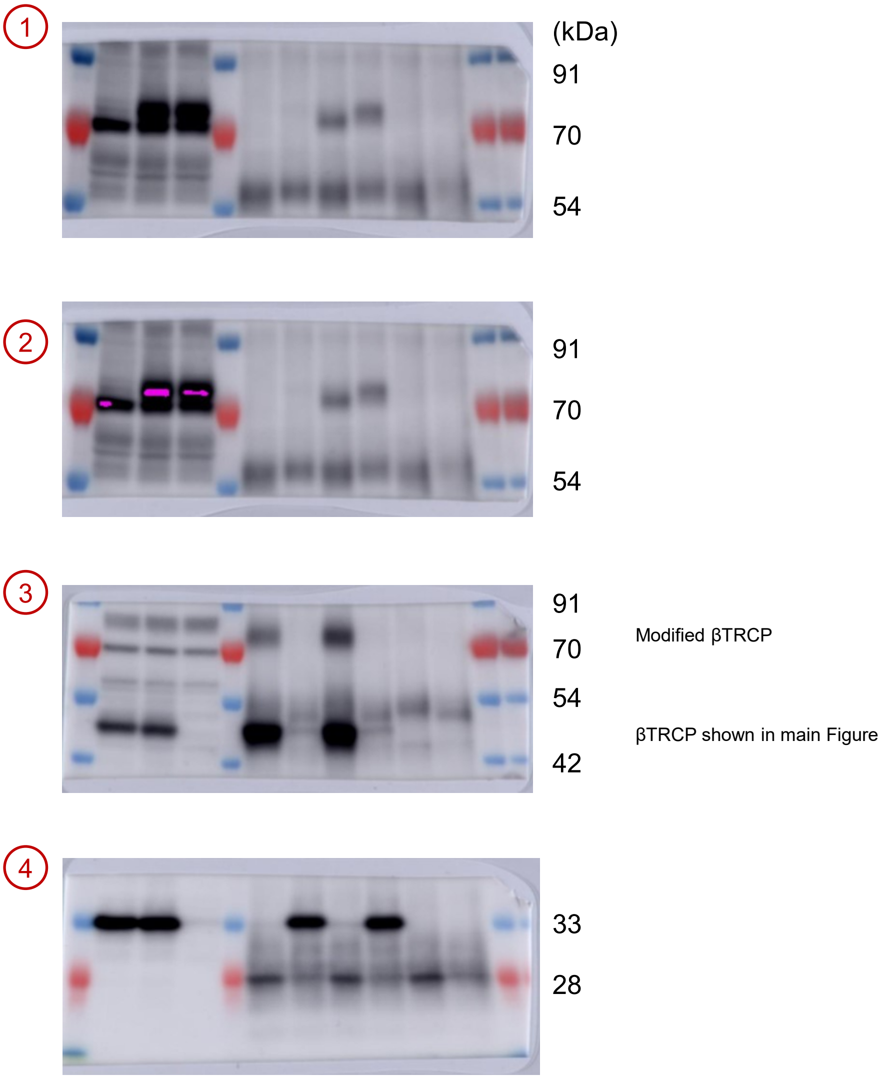
1
(kDa)
91
70
54
2
91
70
54
3
91
70
54
42
Modified βTRCP
βTRCP shown in main Figure
4
33
28

Figure 4-A

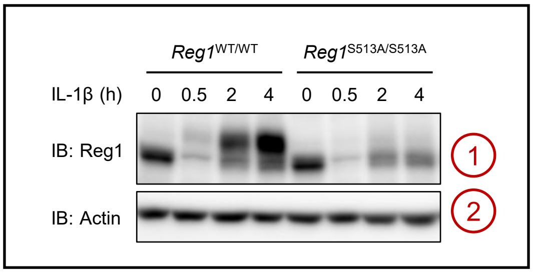
Reg1WT/WT
Reg1S513A/S513A
IL-1β (h)
0 0.5 2 4 0 0.5 2 4
IB: Reg1
IB: Actin
1
2

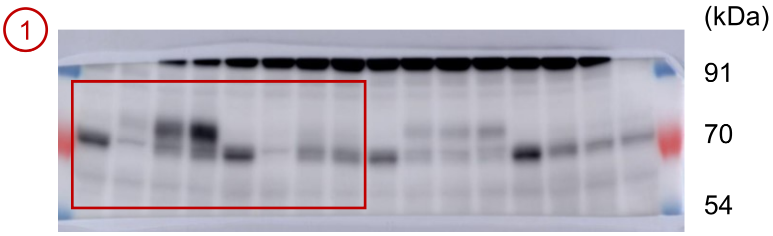
1
(kDa)
91
70
54

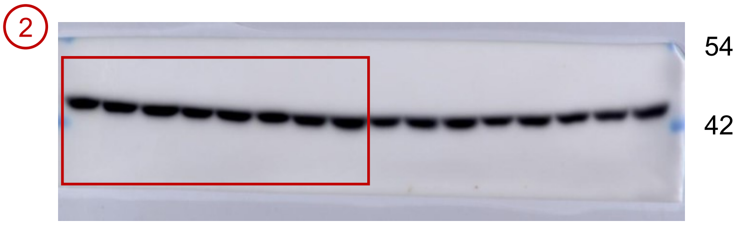
2
54
42

Figure 4-B

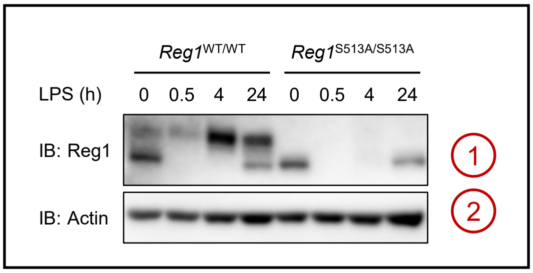
Reg1WT/WT
Reg1S513A/S513A
LPS (h)
0
0.5
4
24
0
0.5
4
24
IB: Reg1
IB: Actin
1
2

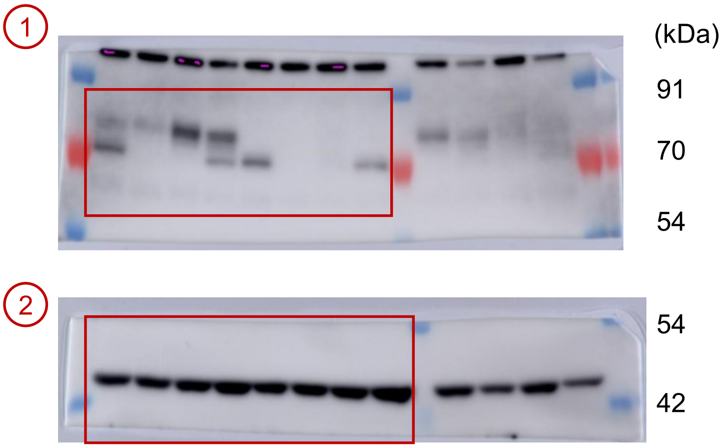
1
2
(kDa)
91
70
54
54
42

Figure 4-C

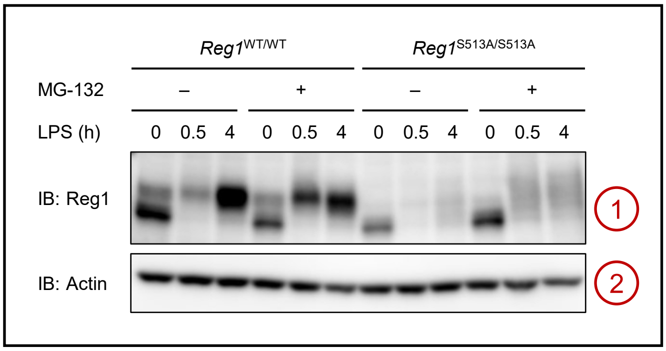

*Reg1*WT/WT
*Reg1*S513A/S513A
MG-132 – + – +
LPS (h) 0 0.5 4 0 0.5 4 0 0.5 4 0 0.5 4
IB: Reg1
IB: Actin
1
2

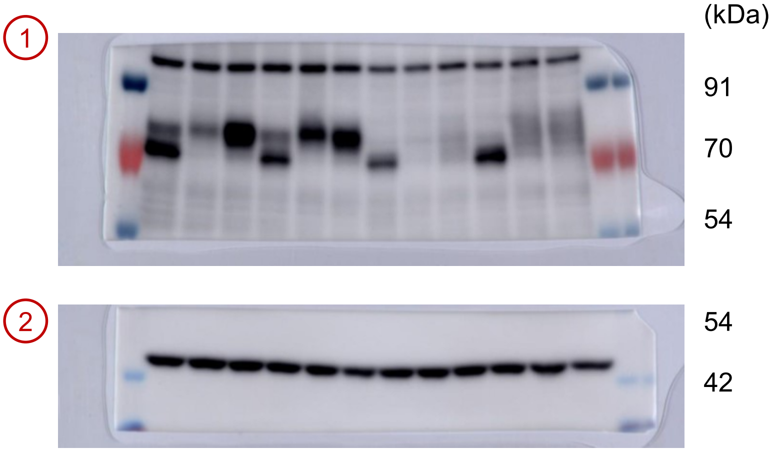

1
2
(kDa)
91
70
54
54
42

Figure 5-A

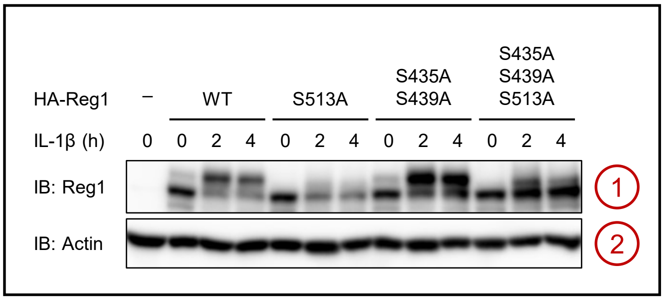
HA-Reg1
–
WT
S513A
S435A
S439A
S435A
S439A
S513A
IL-1β (h)
0 0 2 4 0 2 4 0 2 4 0 2 4
IB: Reg1
IB: Actin
1
2

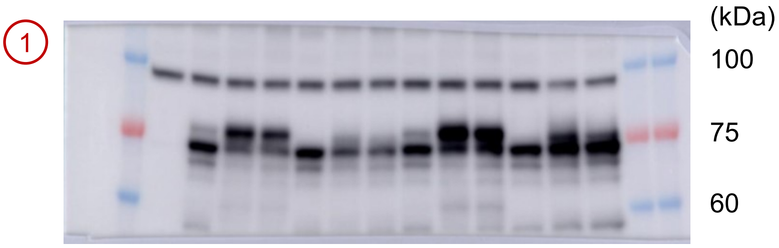
1
(kDa)
100
75
60

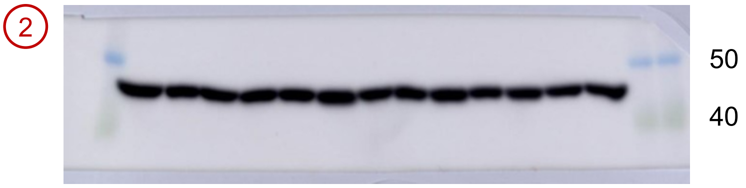
2
50
40

Figure 5-C

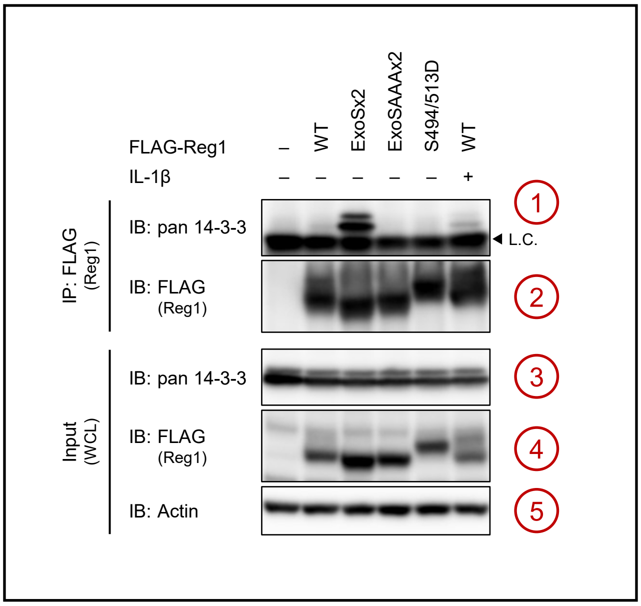

FLAG-Reg1
– WT ExoSx2 ExoSAAAx2 S494/513D WT
IL-1β
– – – – – +
IP: FLAG
(Reg1)
IB: pan 14-3-3
IB: FLAG
(Reg1)
Input
(WCL)
IB: pan 14-3-3
IB: FLAG
(Reg1)
IB: Actin
L.C.
1
2
3
4
5

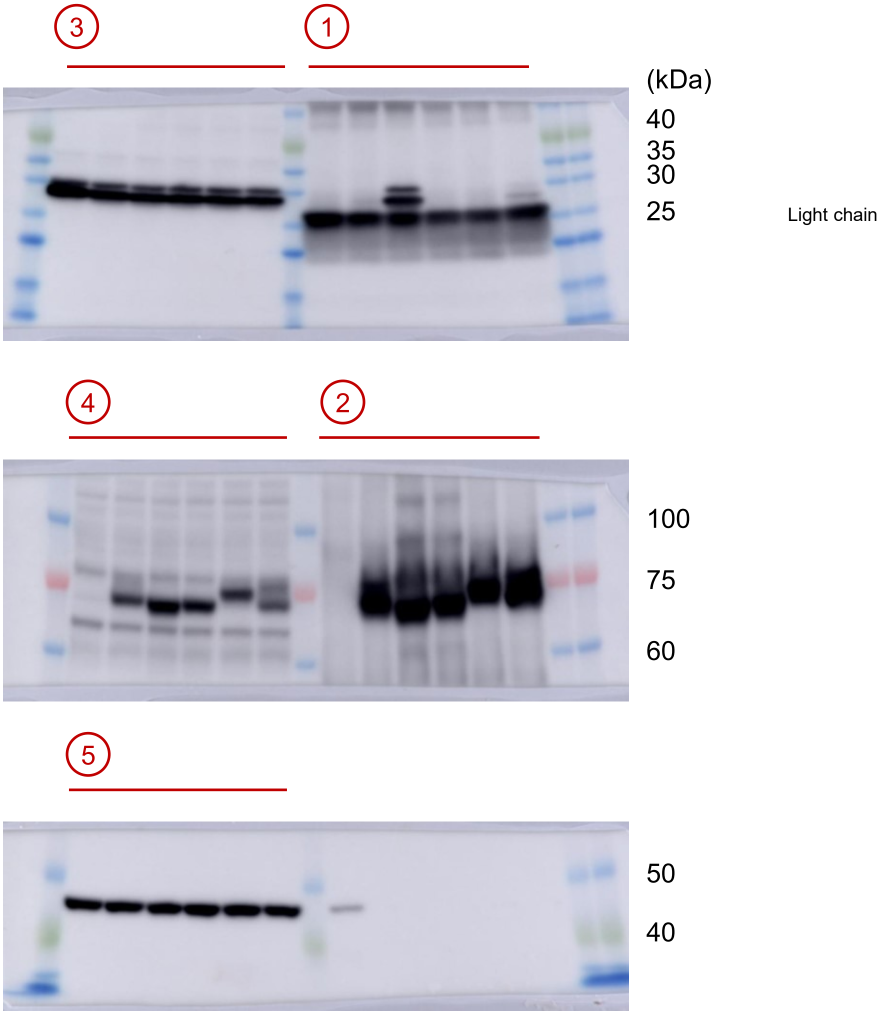

3
1
(kDa)
40
35
30
25
Light chain
4
2
100
75
60
5
50
40
