# Supplementary figures and images for: IRAK1-dependent Regnase-1-14-3-3 complex formation controls Regnase-1-mediated mRNA decay
Source: eLife. 2021 Oct 12;10:e71966. doi: 10.7554/eLife.71966 (PMC8553338; doi:10.7554/eLife.71966)

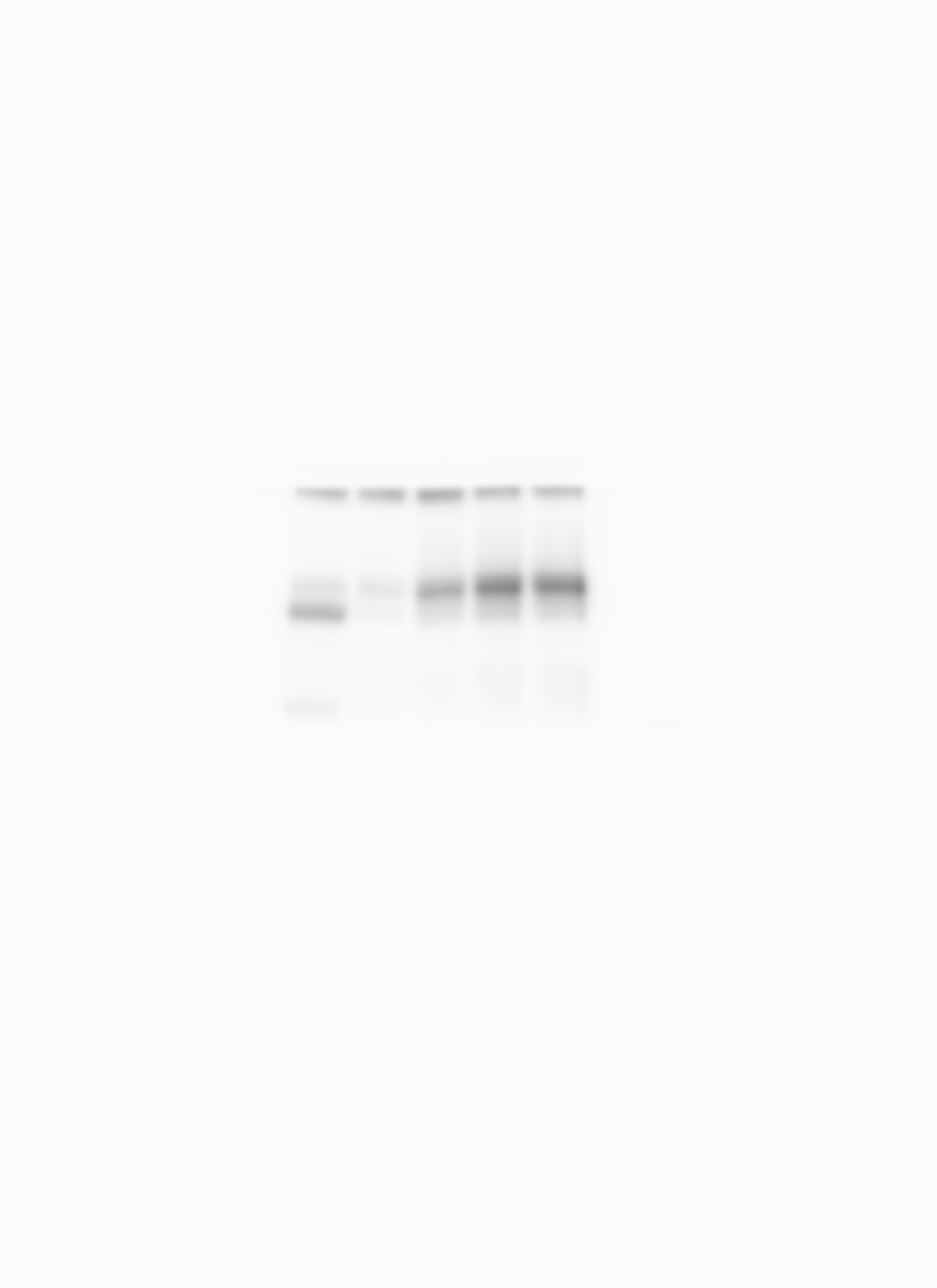

Supplement: Source data 1. [file elife-71966-data1.zip › Source Data Files/Raw Data/Figure 1-C/1, IB-Reg1 (Input).tif]

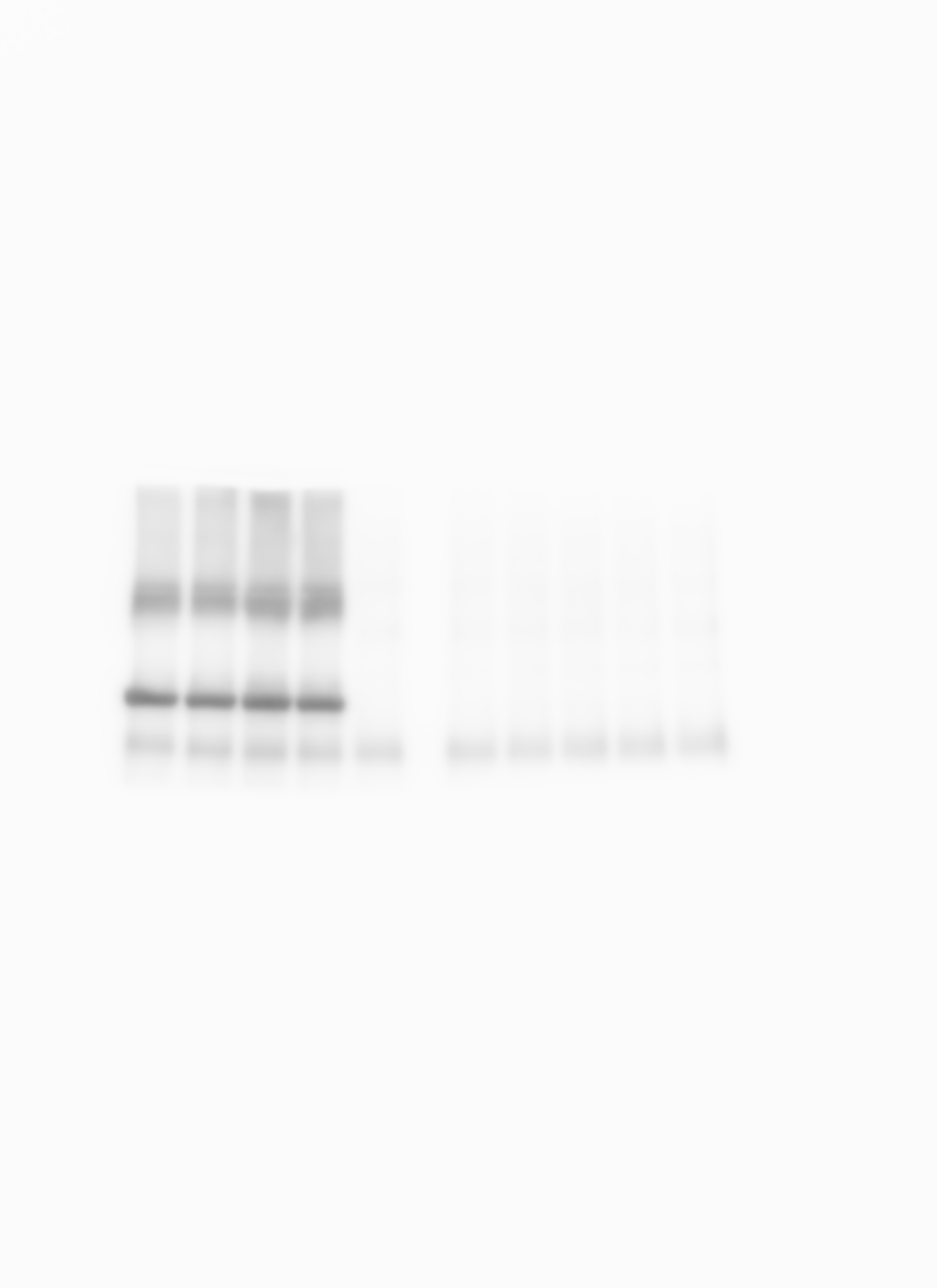

Supplement: Source data 1. [file elife-71966-data1.zip › Source Data Files/Raw Data/Figure 1-C/10, IB-HA (IP-HA).tif]

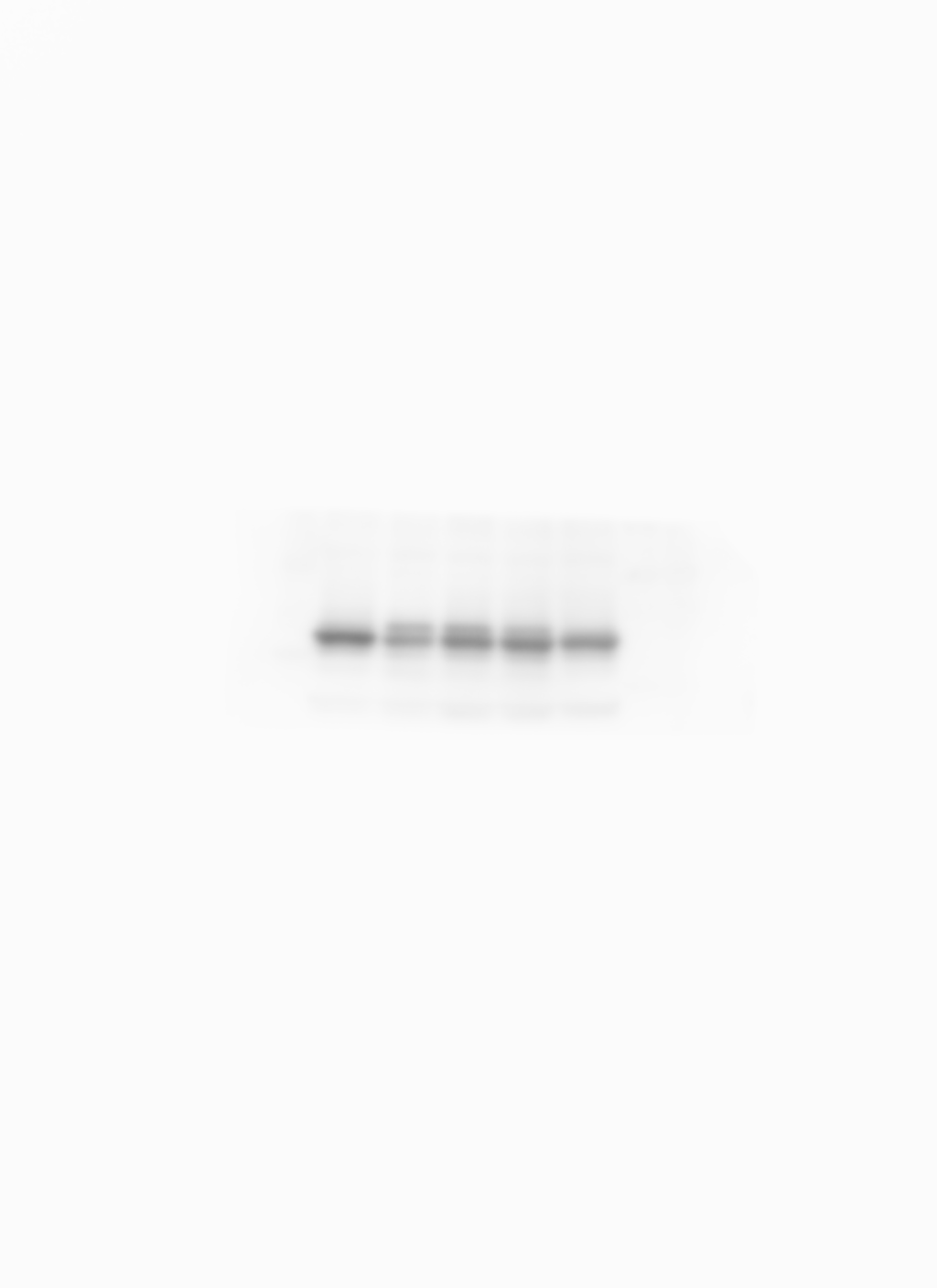

Supplement: Source data 1. [file elife-71966-data1.zip › Source Data Files/Raw Data/Figure 1-C/2, IB-IkBa (Input).tif]

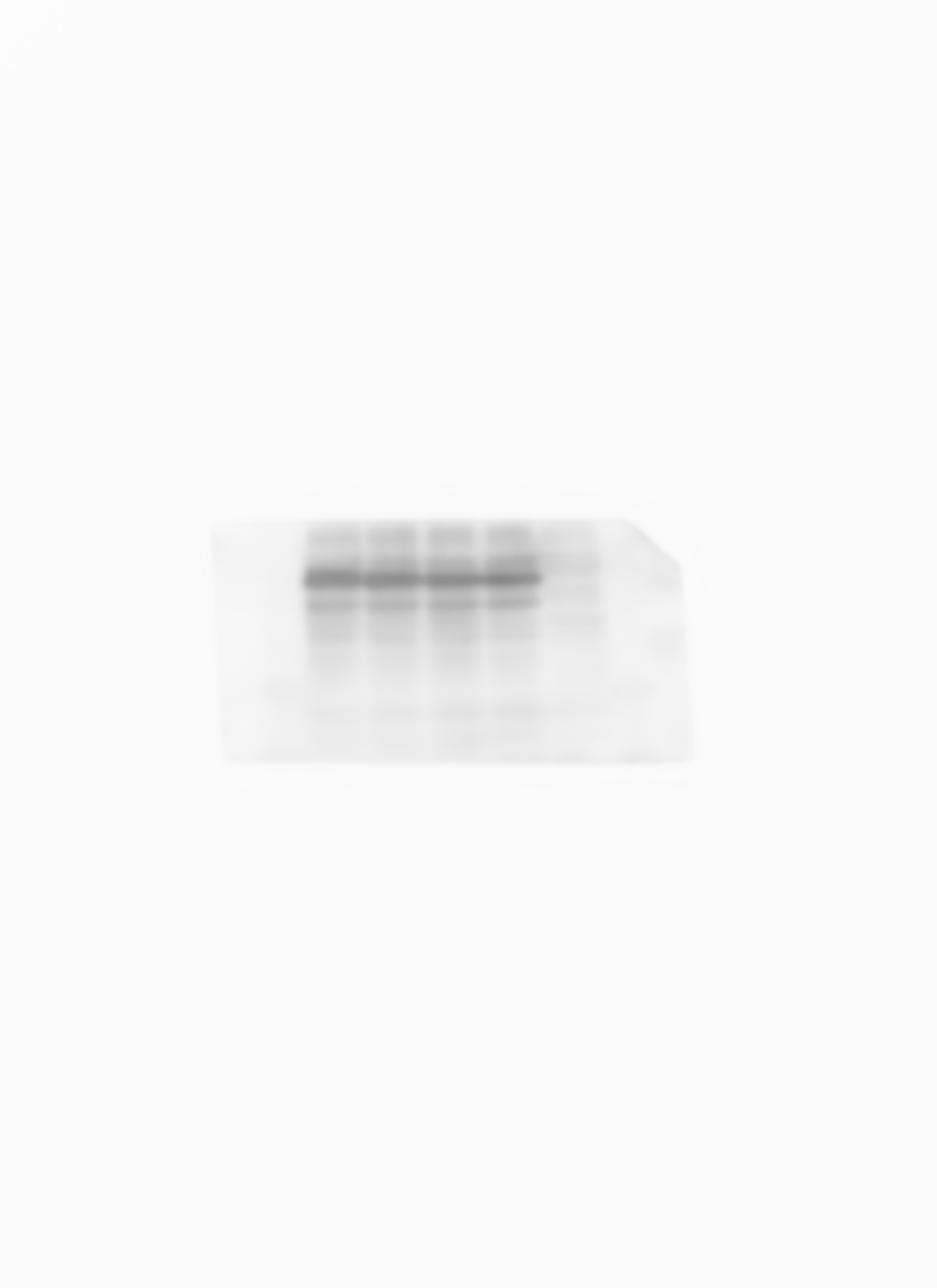

Supplement: Source data 1. [file elife-71966-data1.zip › Source Data Files/Raw Data/Figure 1-C/3, IB-Myc (Input).tif]

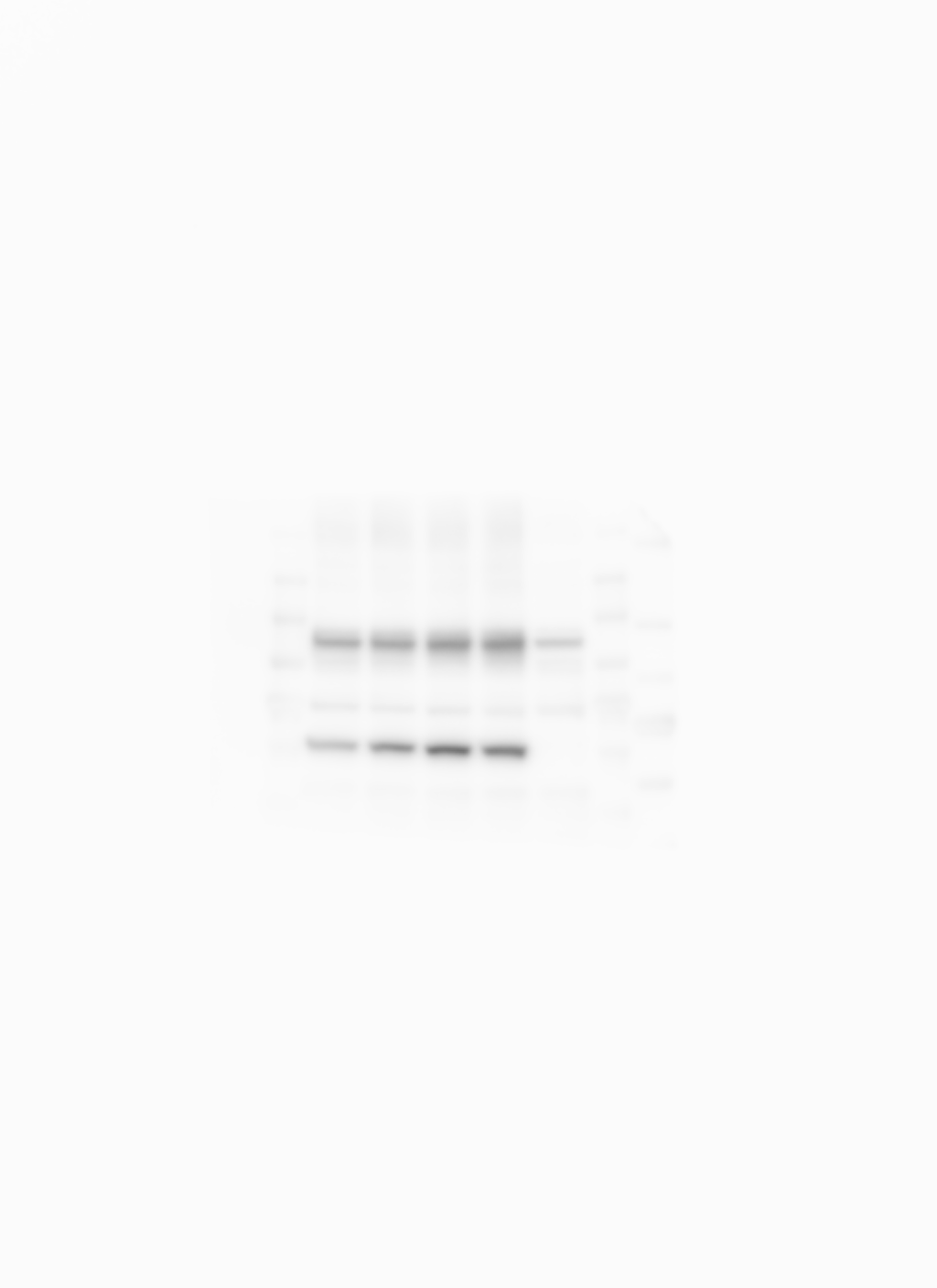

Supplement: Source data 1. [file elife-71966-data1.zip › Source Data Files/Raw Data/Figure 1-C/4, IB-HA (Input).tif]

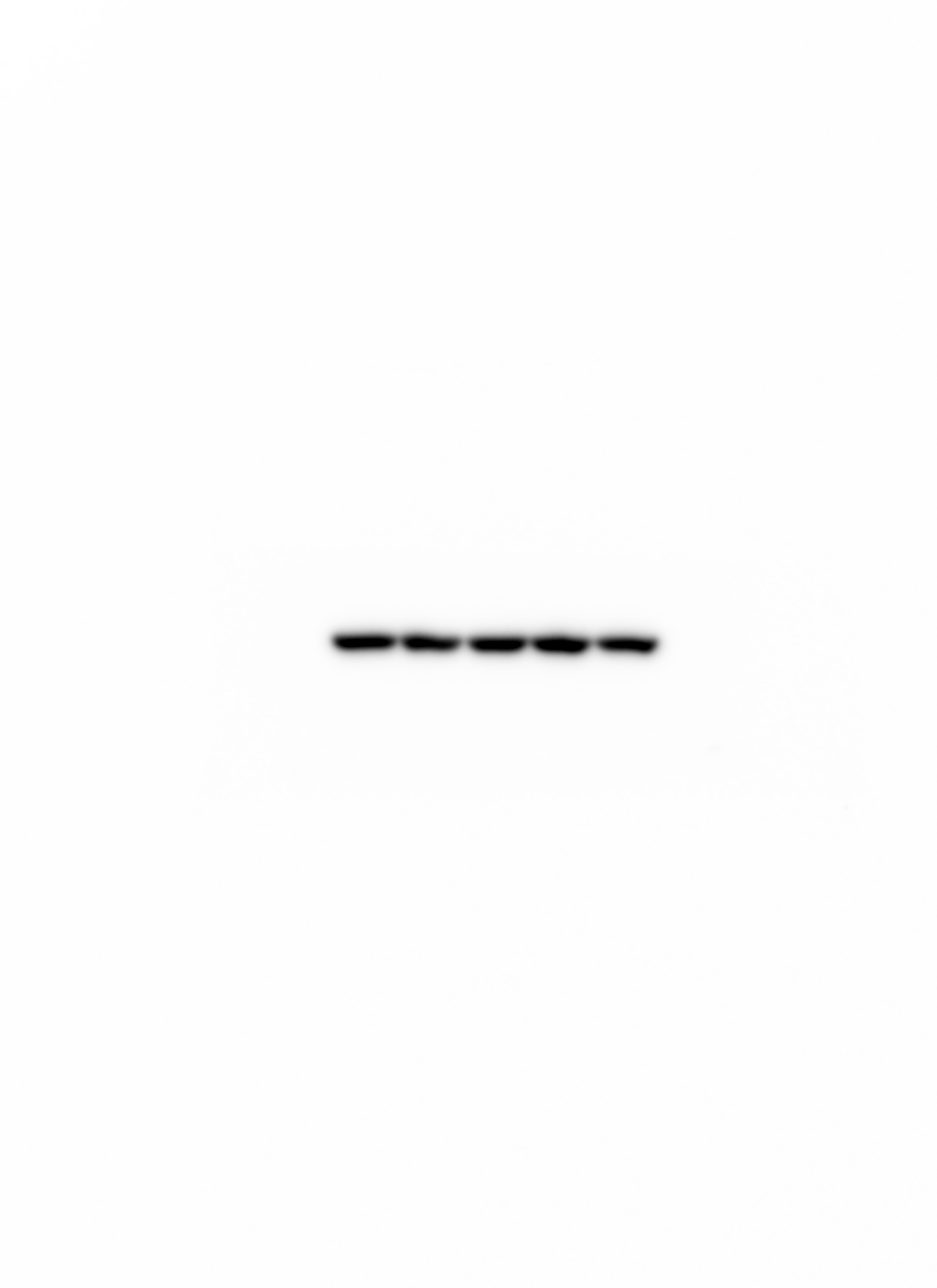

Supplement: Source data 1. [file elife-71966-data1.zip › Source Data Files/Raw Data/Figure 1-C/5, IB-Actin (Input).tif]

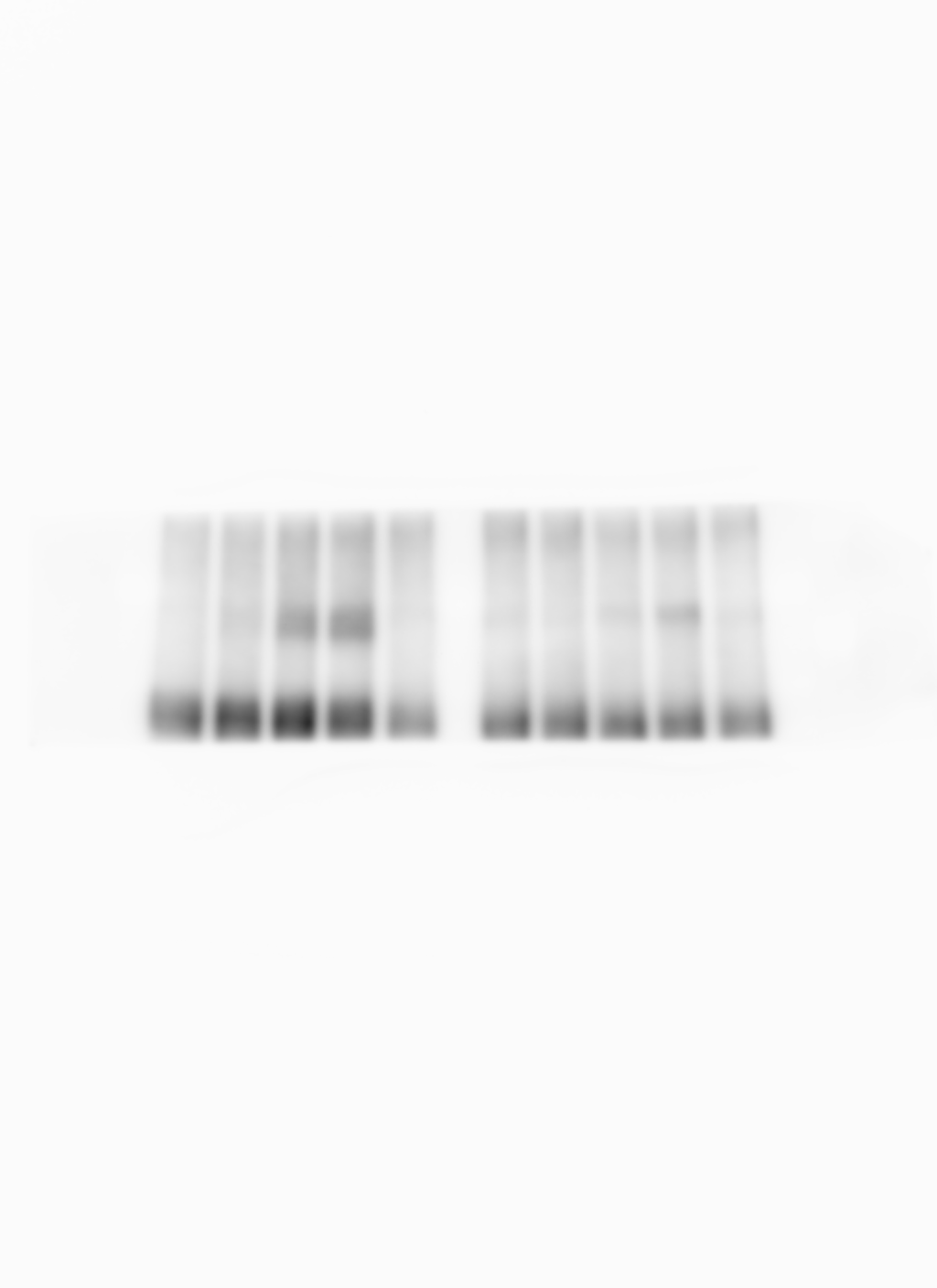

Supplement: Source data 1. [file elife-71966-data1.zip › Source Data Files/Raw Data/Figure 1-C/6 and 8, IB-Reg1 (IP-Myc or IP-HA).tif]

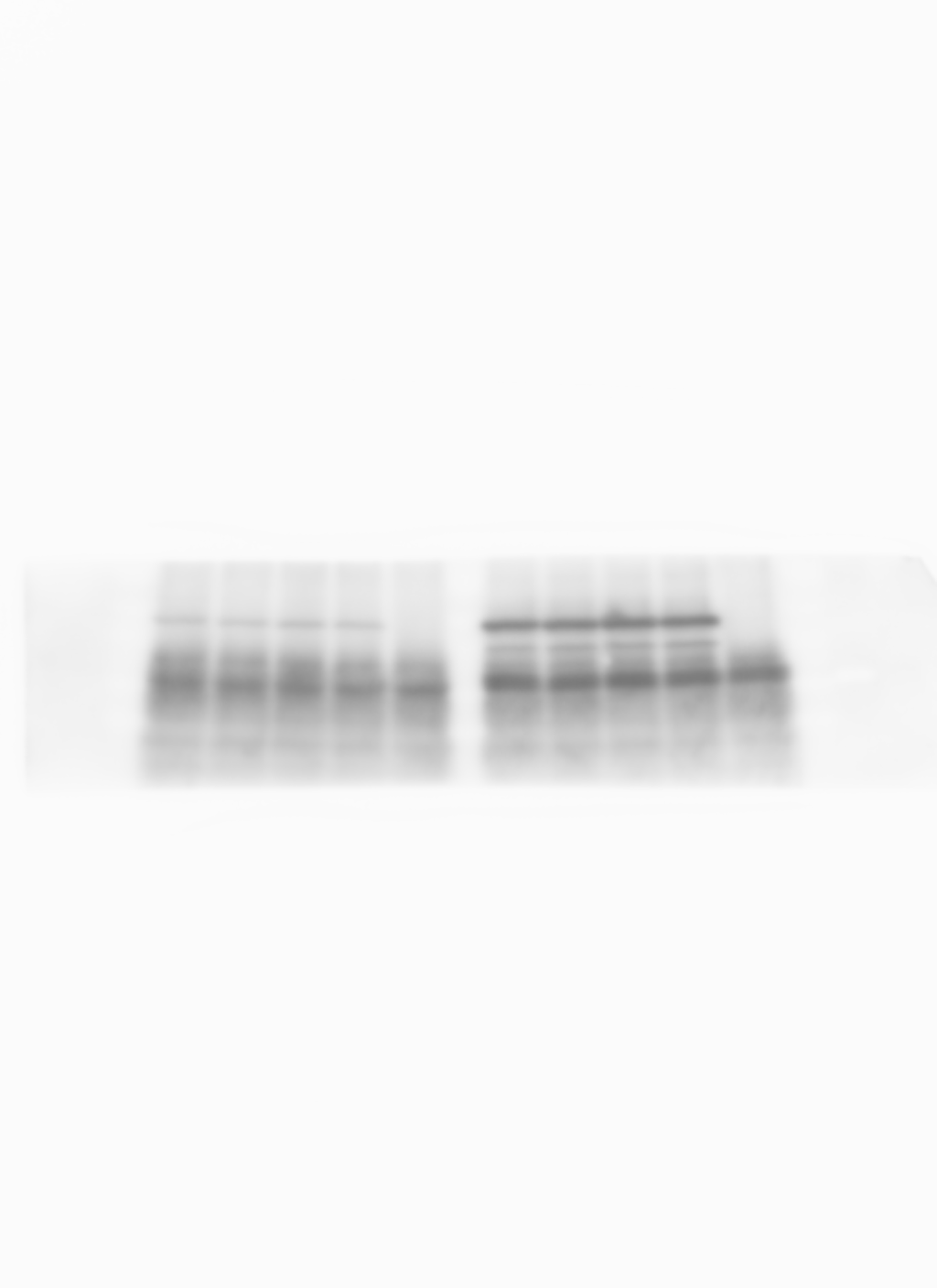

Supplement: Source data 1. [file elife-71966-data1.zip › Source Data Files/Raw Data/Figure 1-C/7, IB-Myc (IP-Myc).tif]

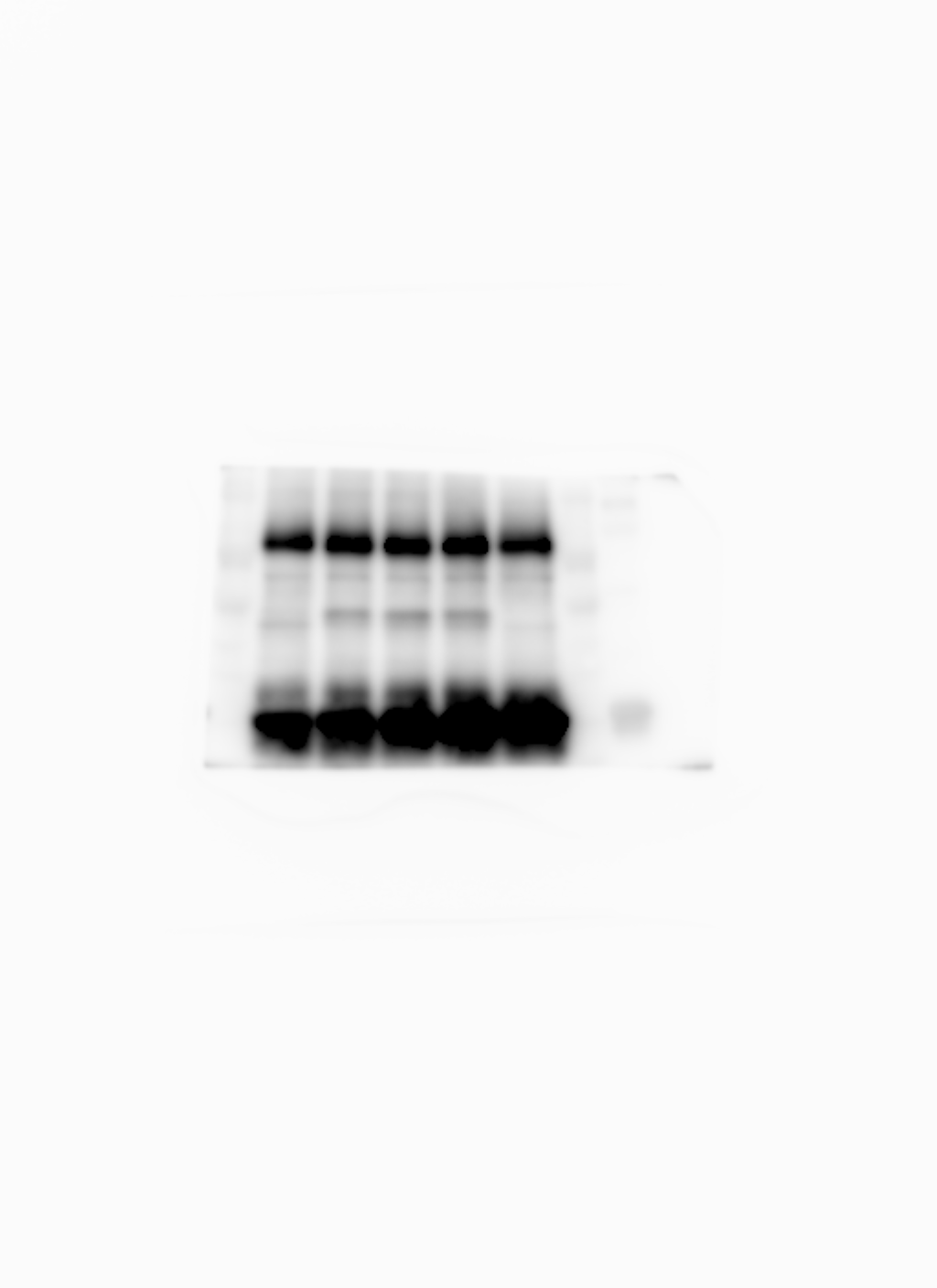

Supplement: Source data 1. [file elife-71966-data1.zip › Source Data Files/Raw Data/Figure 1-C/9, IB-IkBa (IP-HA).tif]

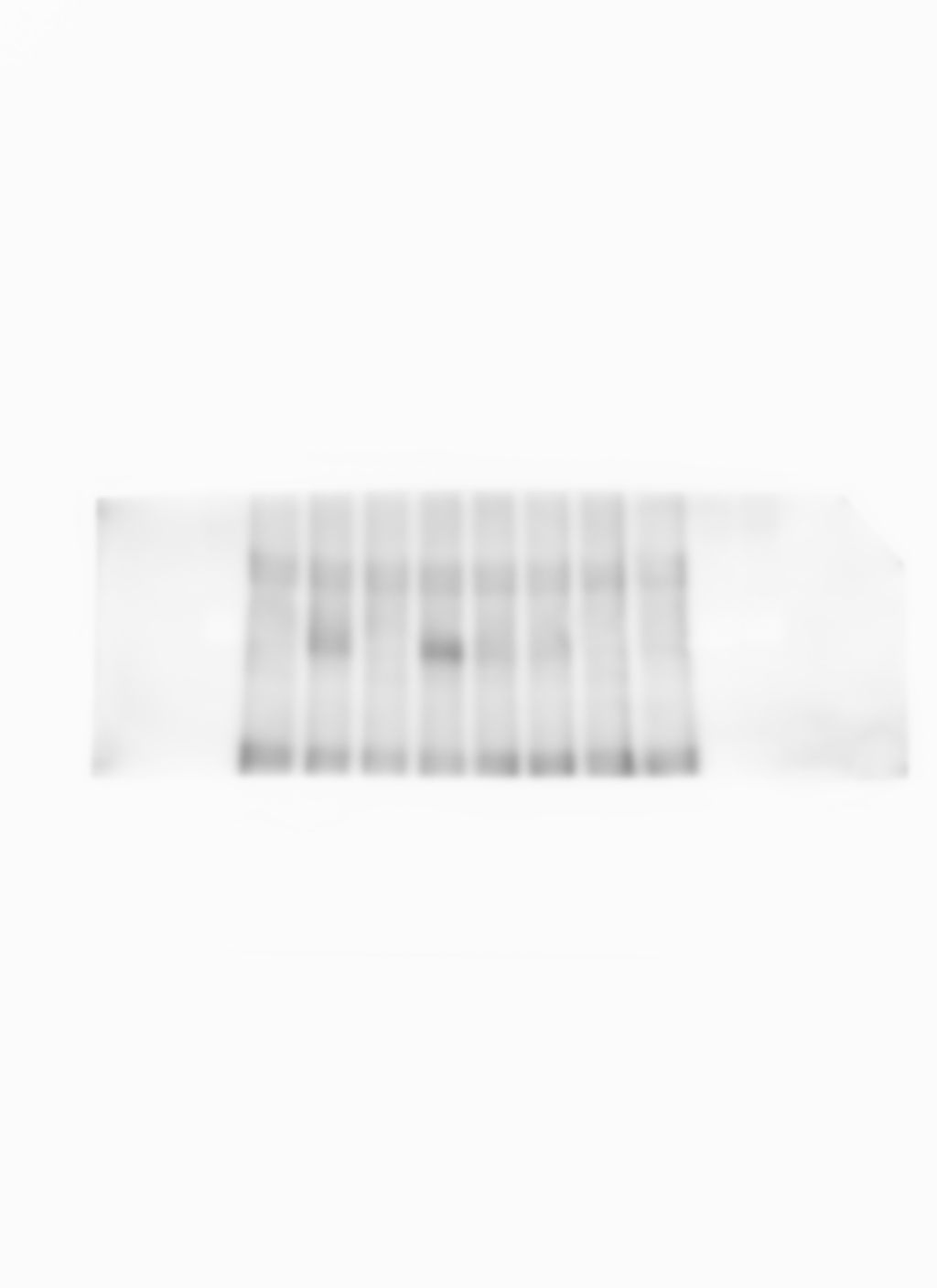

Supplement: Source data 1. [file elife-71966-data1.zip › Source Data Files/Raw Data/Figure 1-D/1, IB-Reg1 (IP).tif]

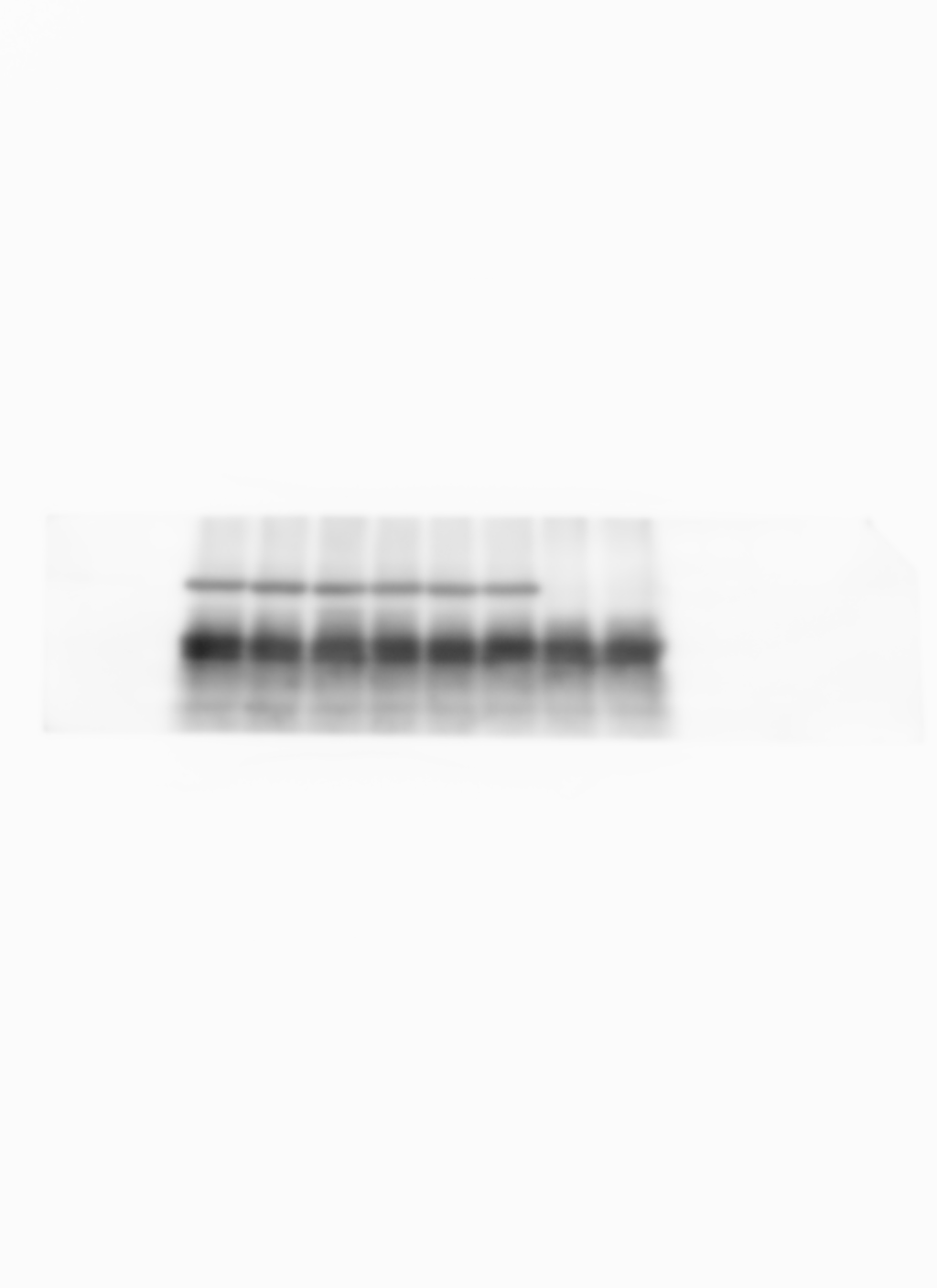

Supplement: Source data 1. [file elife-71966-data1.zip › Source Data Files/Raw Data/Figure 1-D/2, IB-Myc (IP).tif]

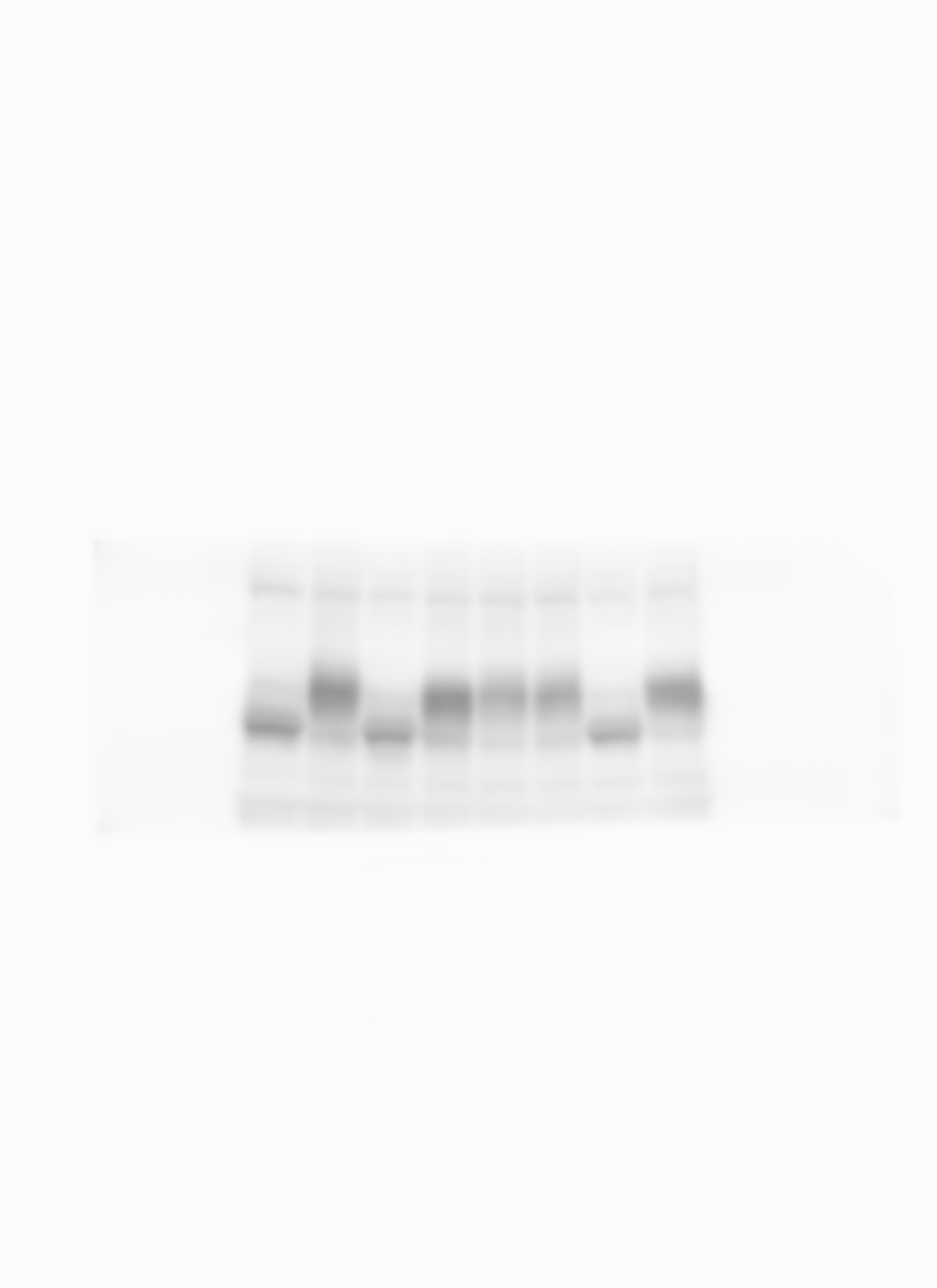

Supplement: Source data 1. [file elife-71966-data1.zip › Source Data Files/Raw Data/Figure 1-D/3, IB-Reg1 (Input).tif]

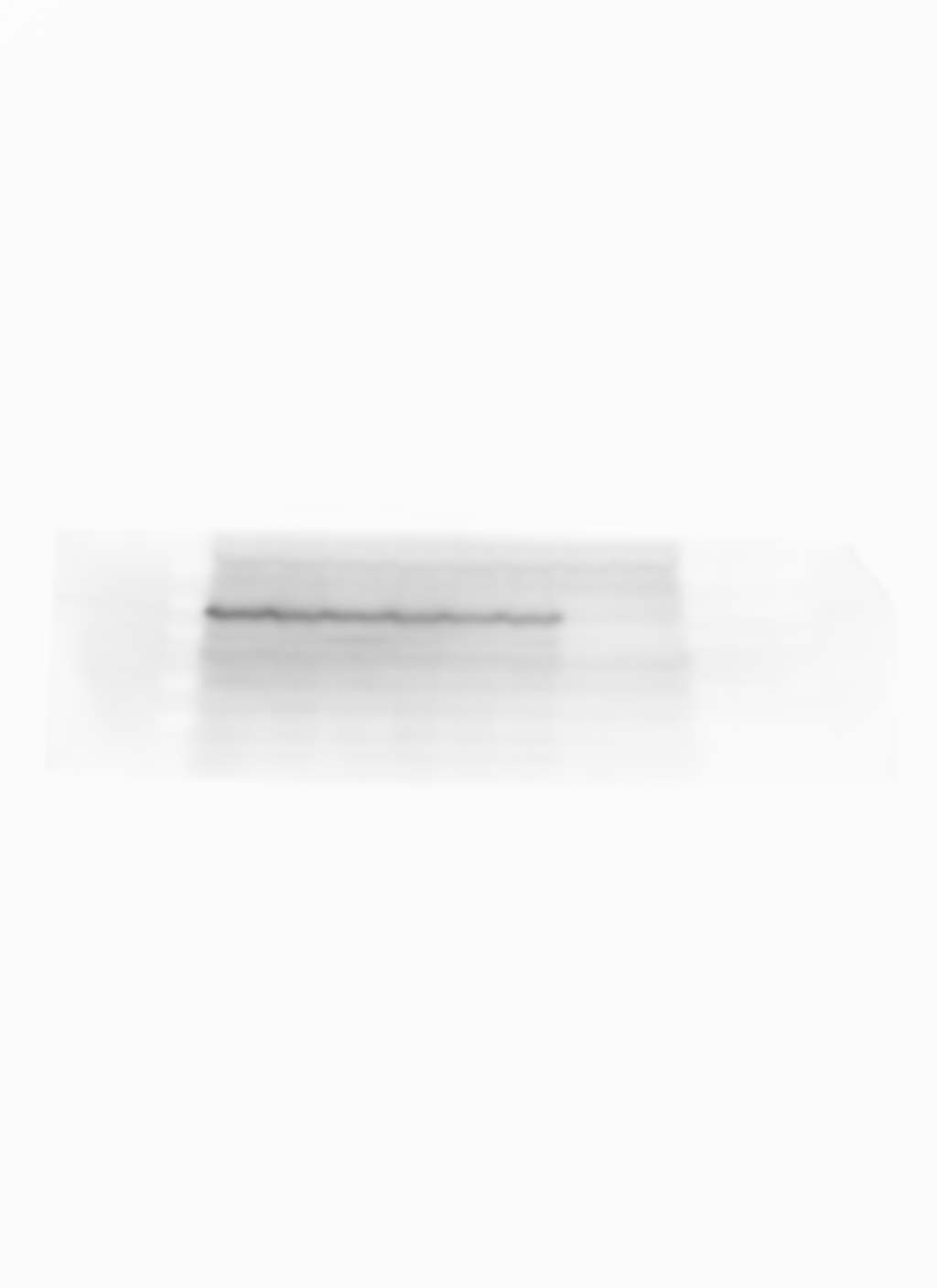

Supplement: Source data 1. [file elife-71966-data1.zip › Source Data Files/Raw Data/Figure 1-D/4, IB-Myc (Input).tif]

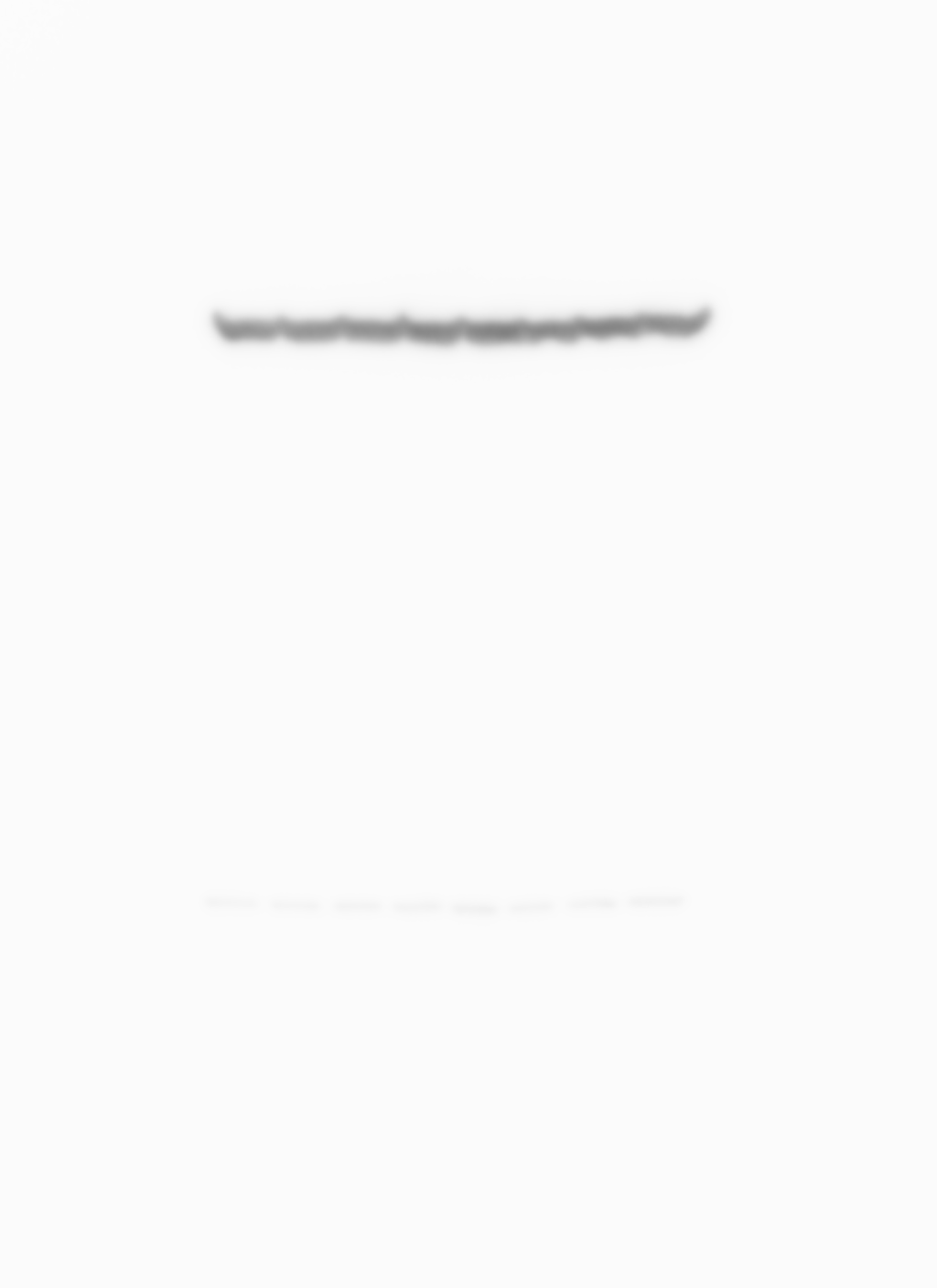

Supplement: Source data 1. [file elife-71966-data1.zip › Source Data Files/Raw Data/Figure 1-D/5, IB-Actin (Input).tif]

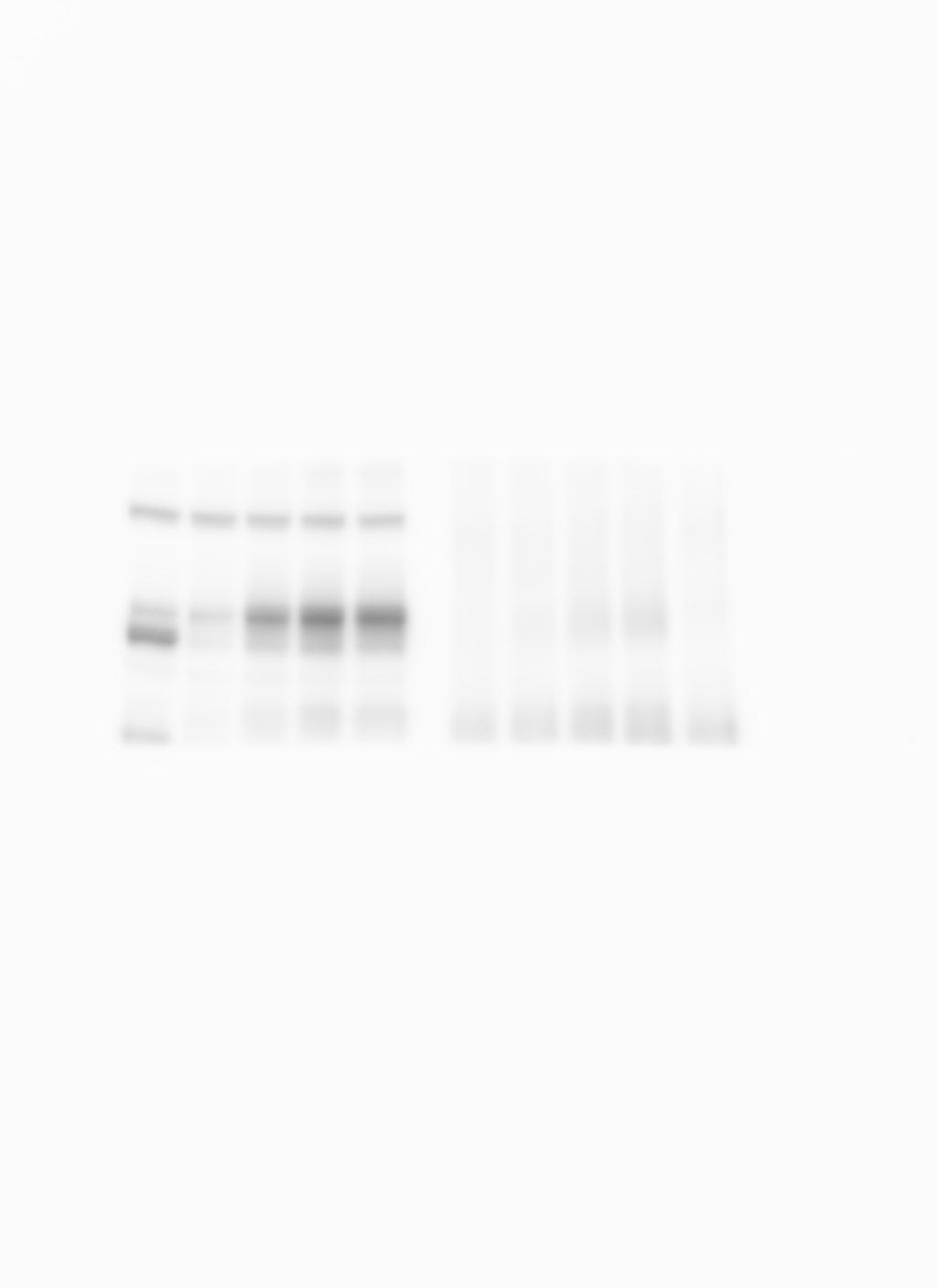

Supplement: Source data 1. [file elife-71966-data1.zip › Source Data Files/Raw Data/Figure 1-figure supplement 1/1, IB-Reg1 (SE).tif]

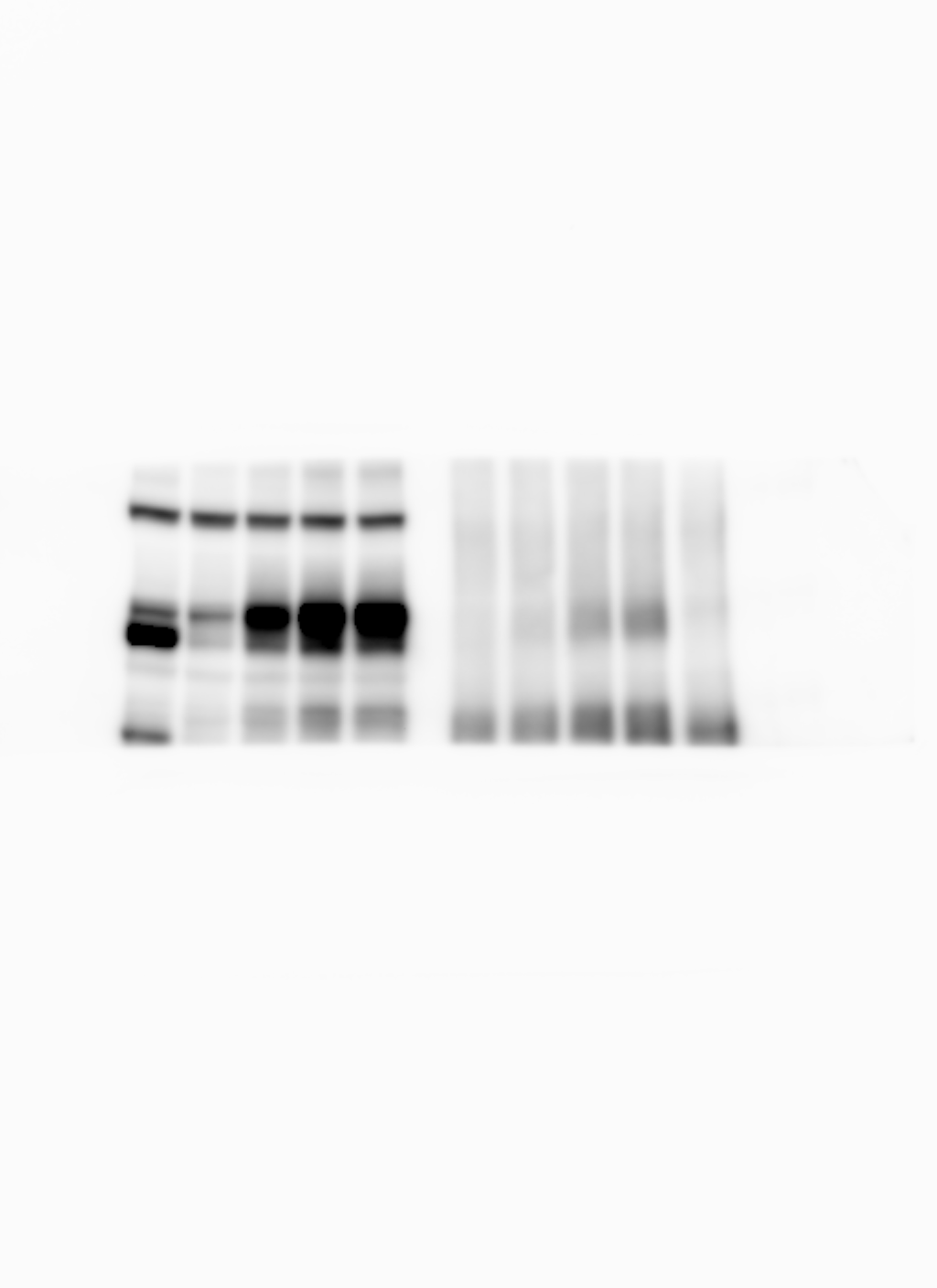

Supplement: Source data 1. [file elife-71966-data1.zip › Source Data Files/Raw Data/Figure 1-figure supplement 1/2, IB-Reg1 (LE).tif]

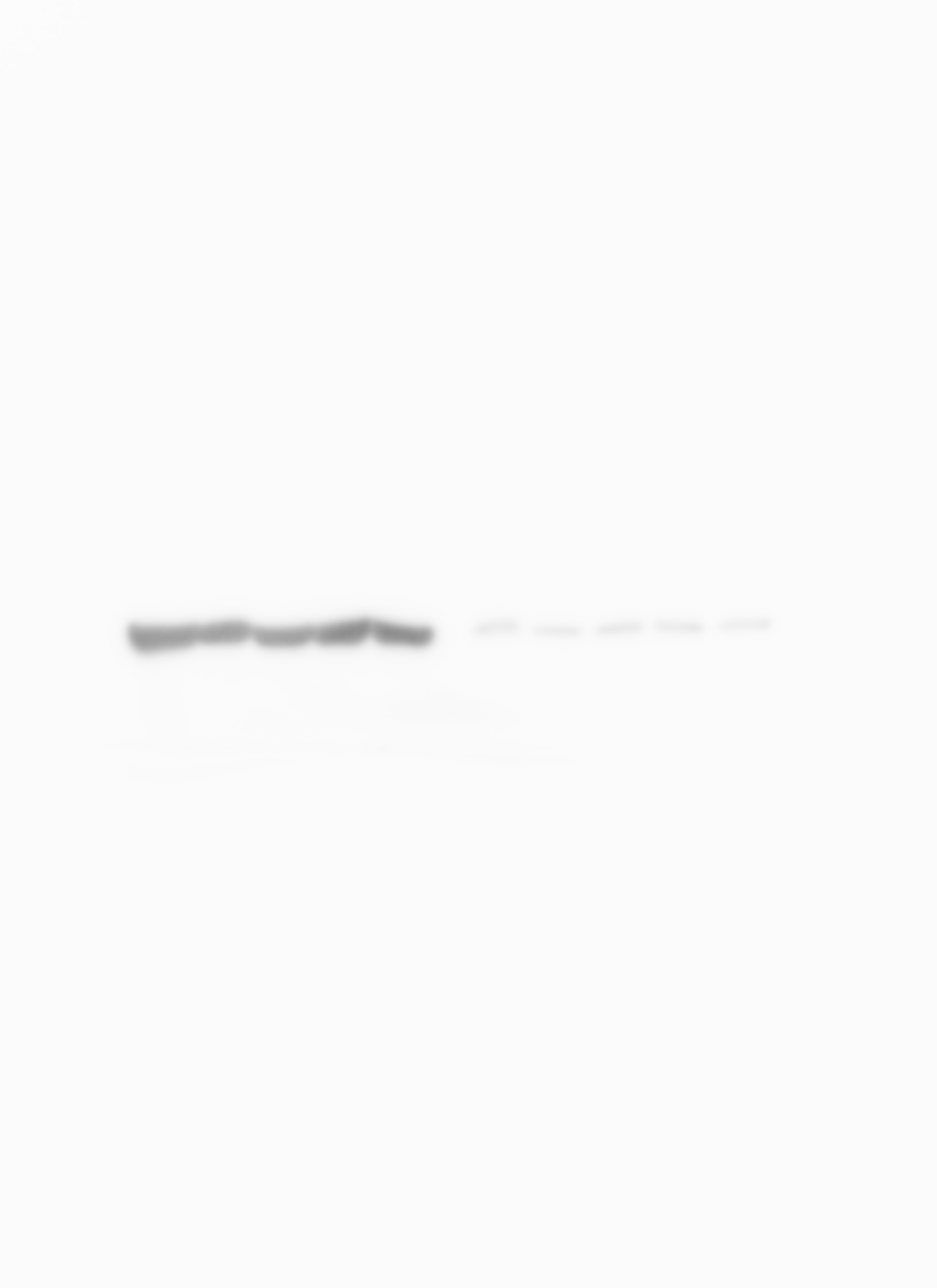

Supplement: Source data 1. [file elife-71966-data1.zip › Source Data Files/Raw Data/Figure 1-figure supplement 1/3, IB-Actin (SE).tif]

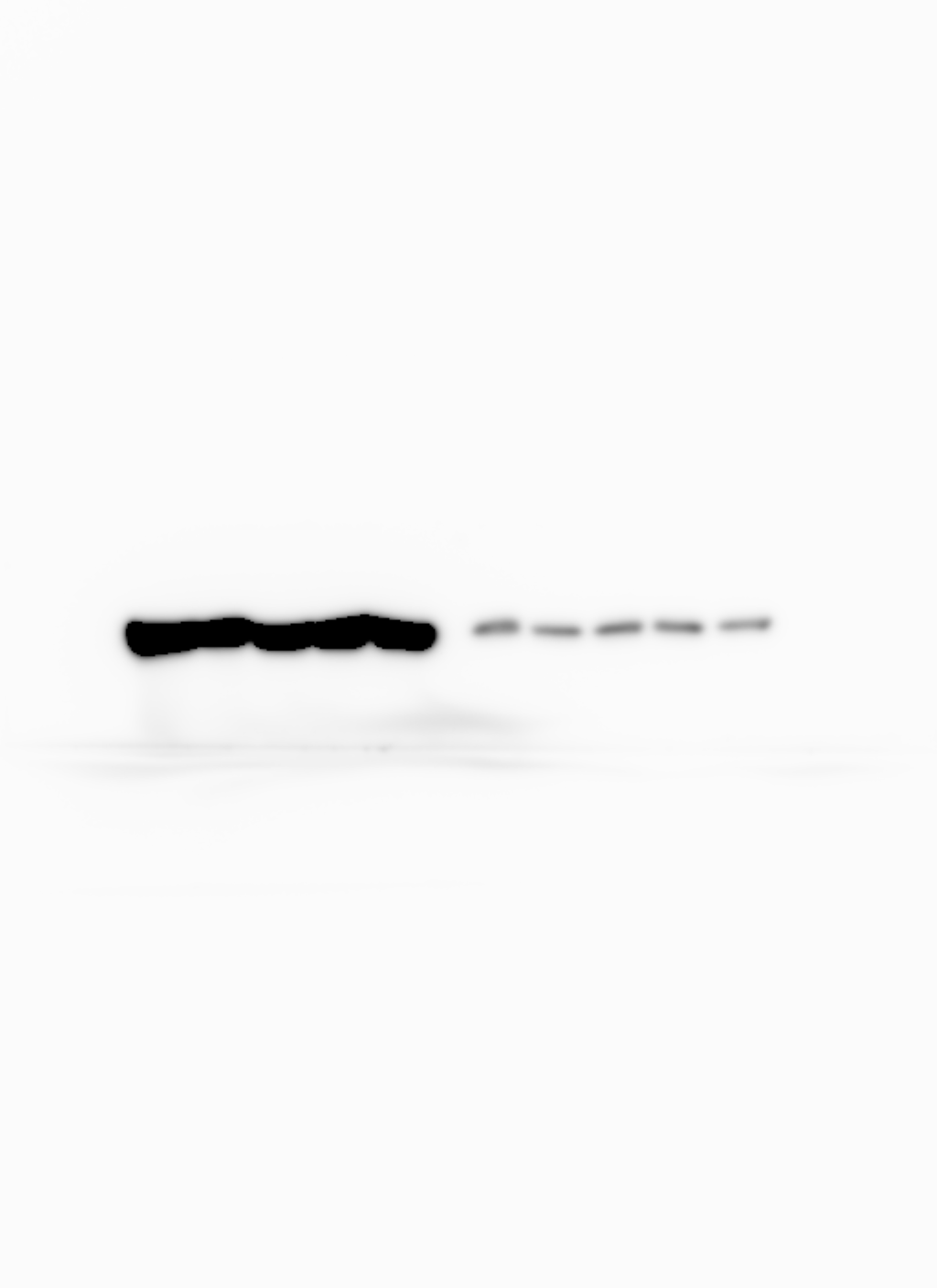

Supplement: Source data 1. [file elife-71966-data1.zip › Source Data Files/Raw Data/Figure 1-figure supplement 1/4, IB-Actin (LE).tif]

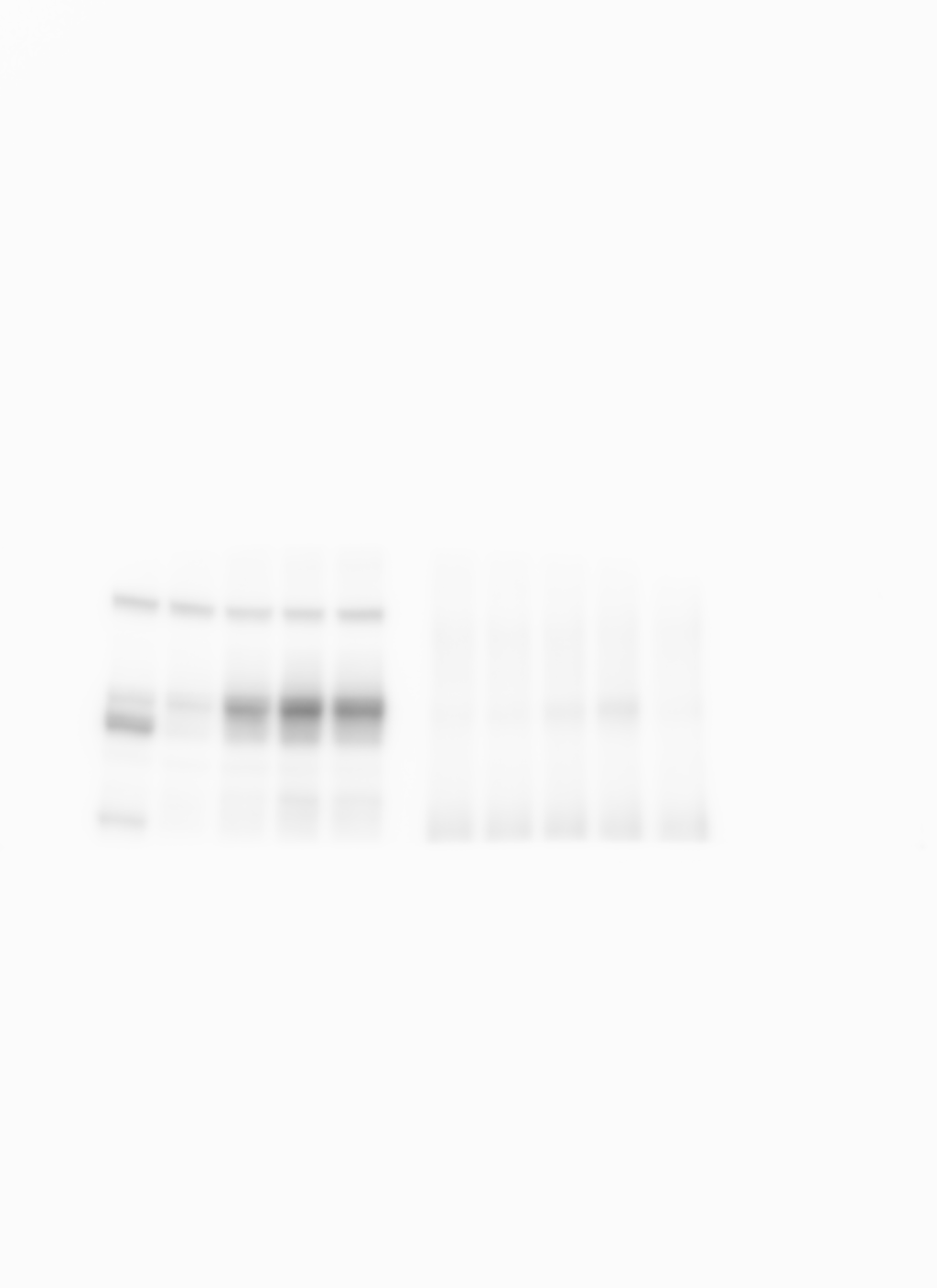

Supplement: Source data 1. [file elife-71966-data1.zip › Source Data Files/Raw Data/Figure 1-figure supplement 1/5, IB-Reg1 (SE).tif]

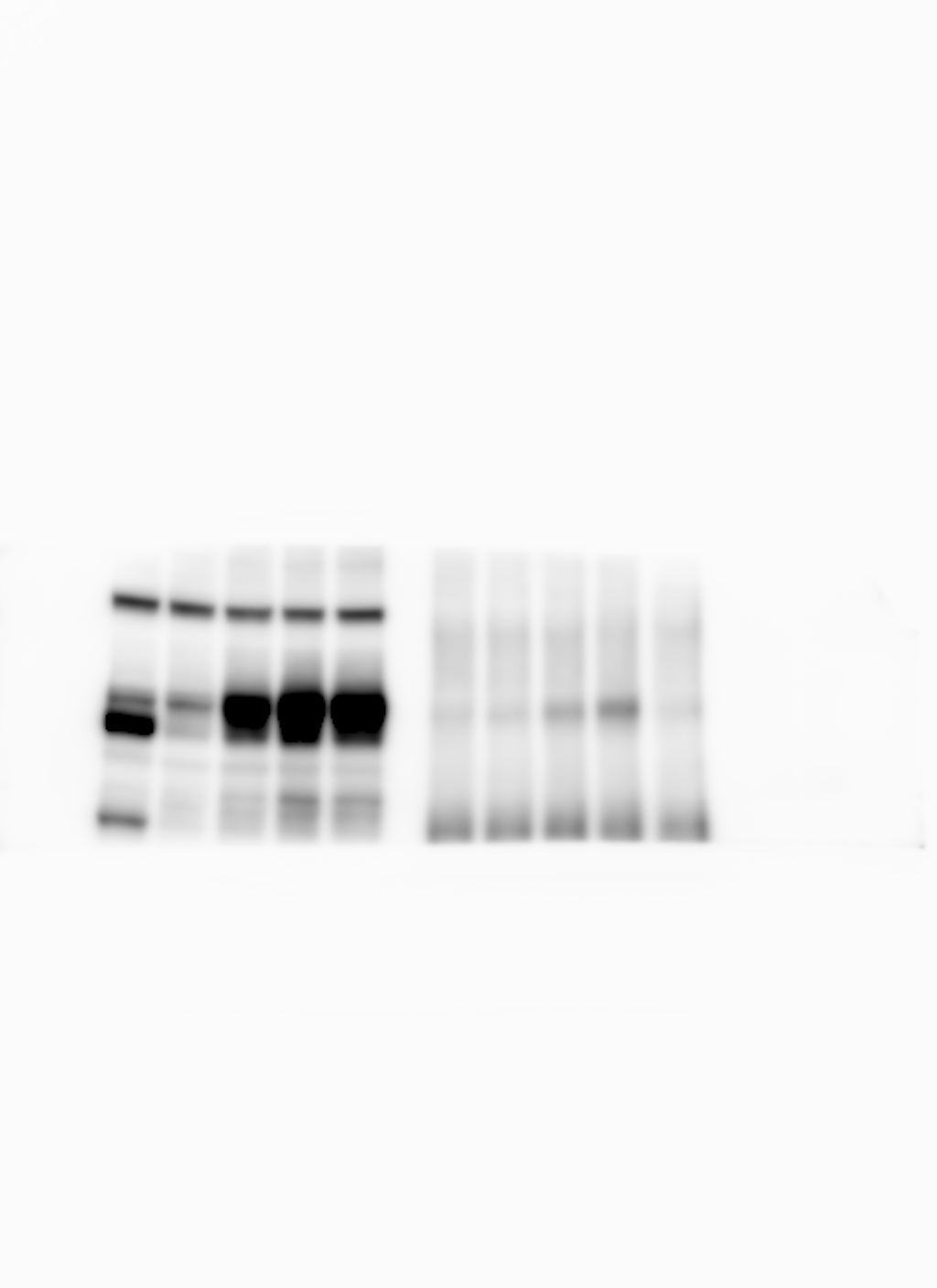

Supplement: Source data 1. [file elife-71966-data1.zip › Source Data Files/Raw Data/Figure 1-figure supplement 1/6, IB-Reg1 (LE).tif]

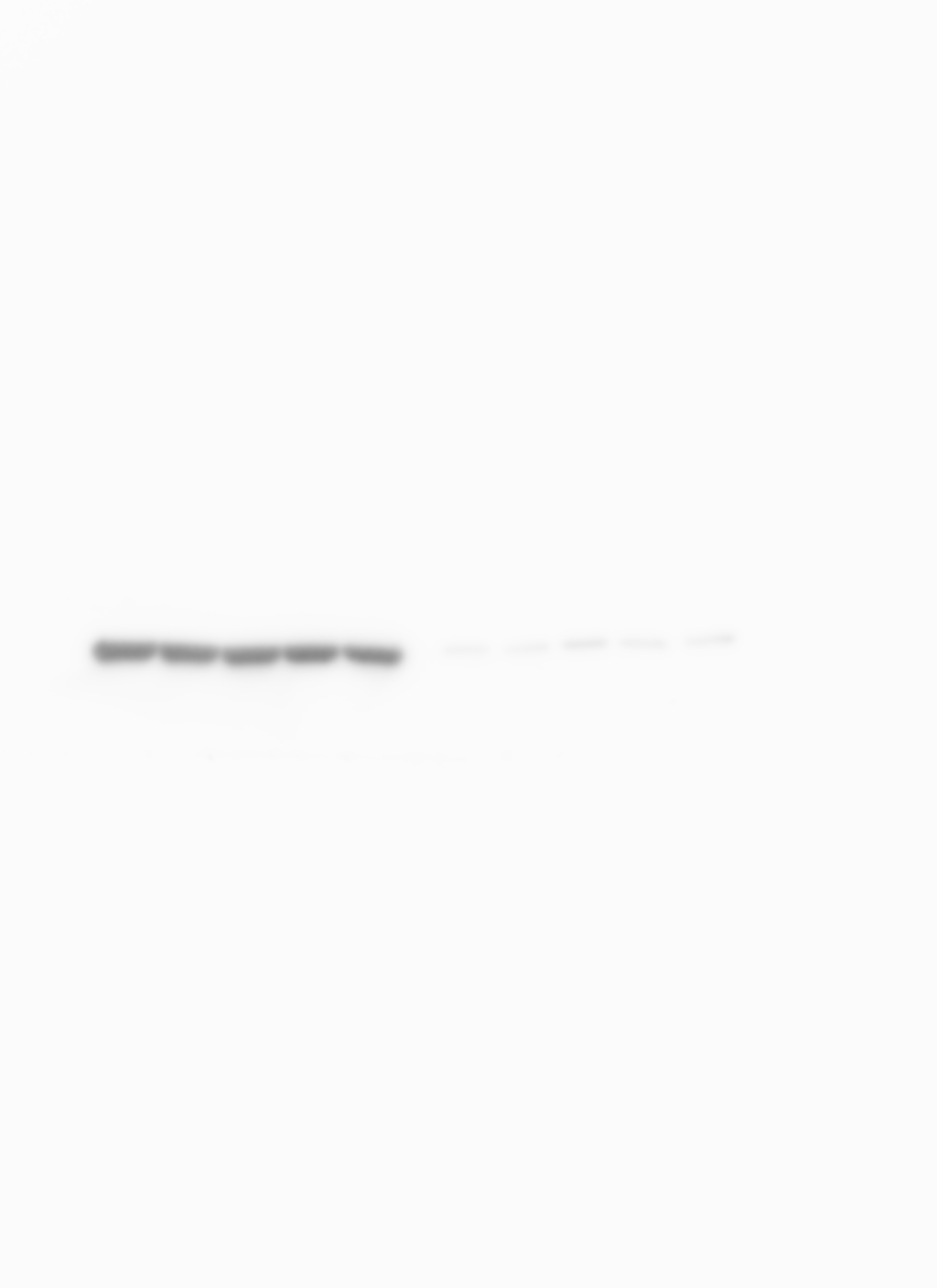

Supplement: Source data 1. [file elife-71966-data1.zip › Source Data Files/Raw Data/Figure 1-figure supplement 1/7, IB-Actin (SE).tif]

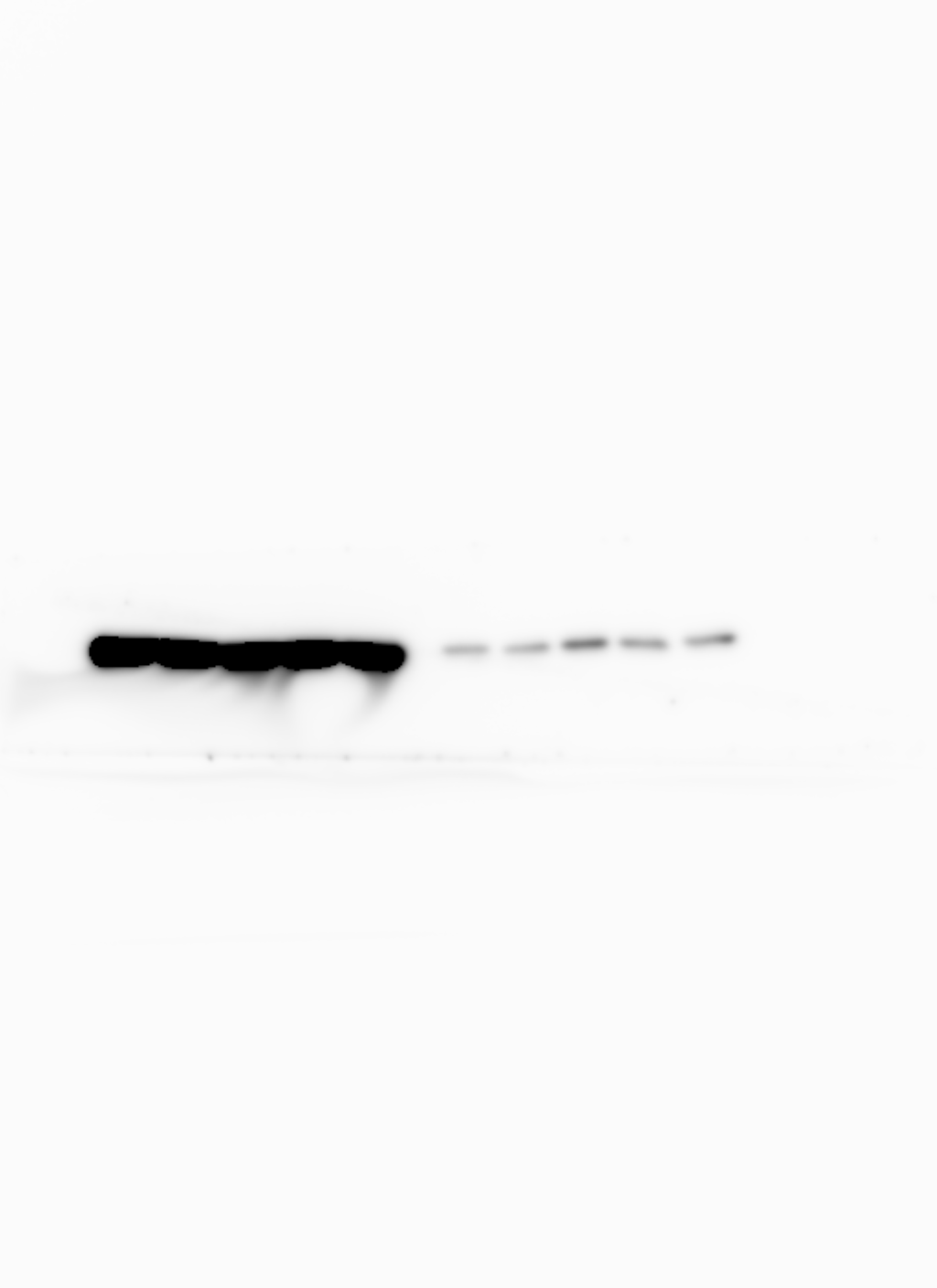

Supplement: Source data 1. [file elife-71966-data1.zip › Source Data Files/Raw Data/Figure 1-figure supplement 1/8, IB-Actin (LE).tif]

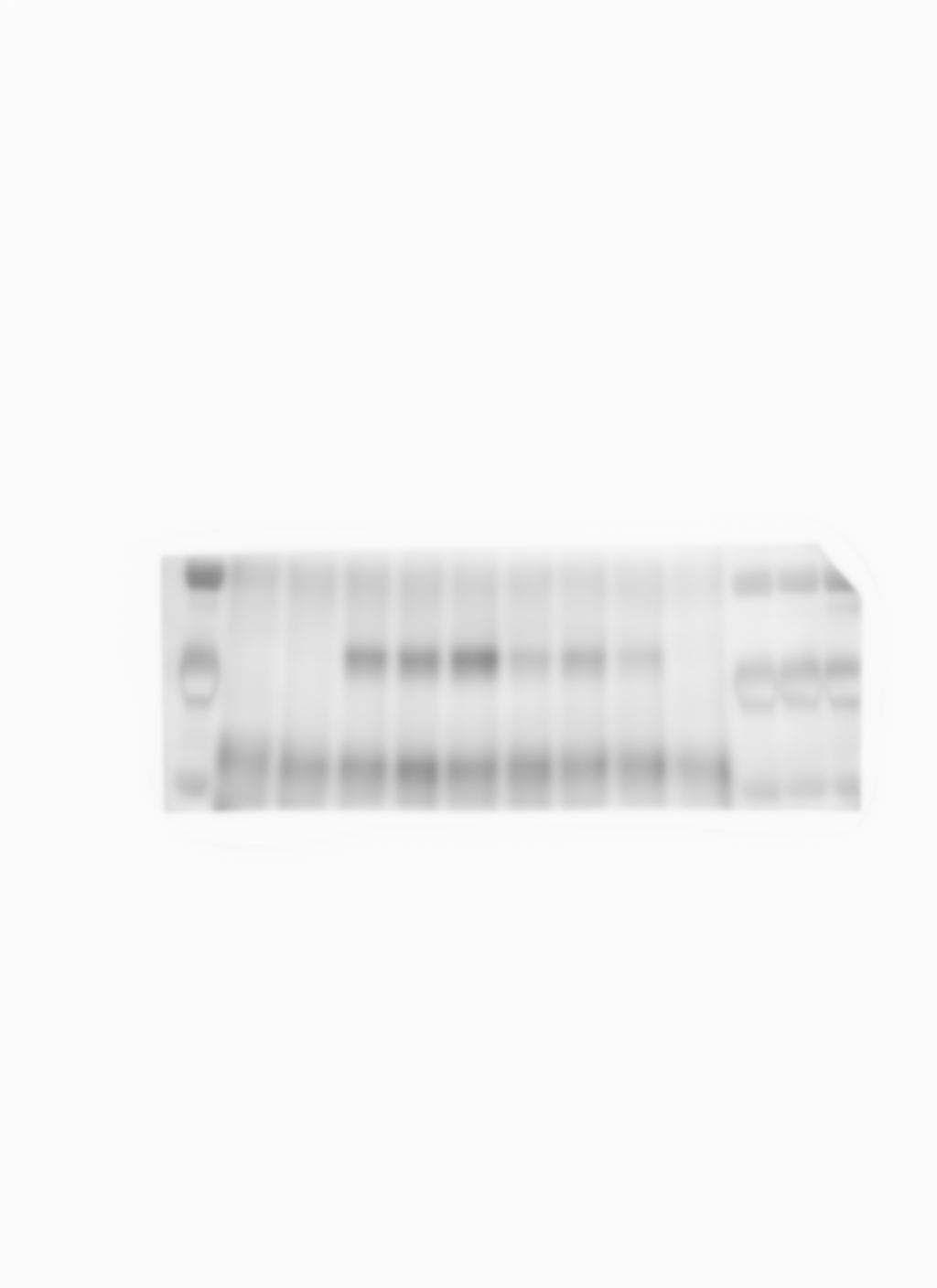

Supplement: Source data 1. [file elife-71966-data1.zip › Source Data Files/Raw Data/Figure 1-figure supplement 2/1, IB-Reg1 (IP).tif]

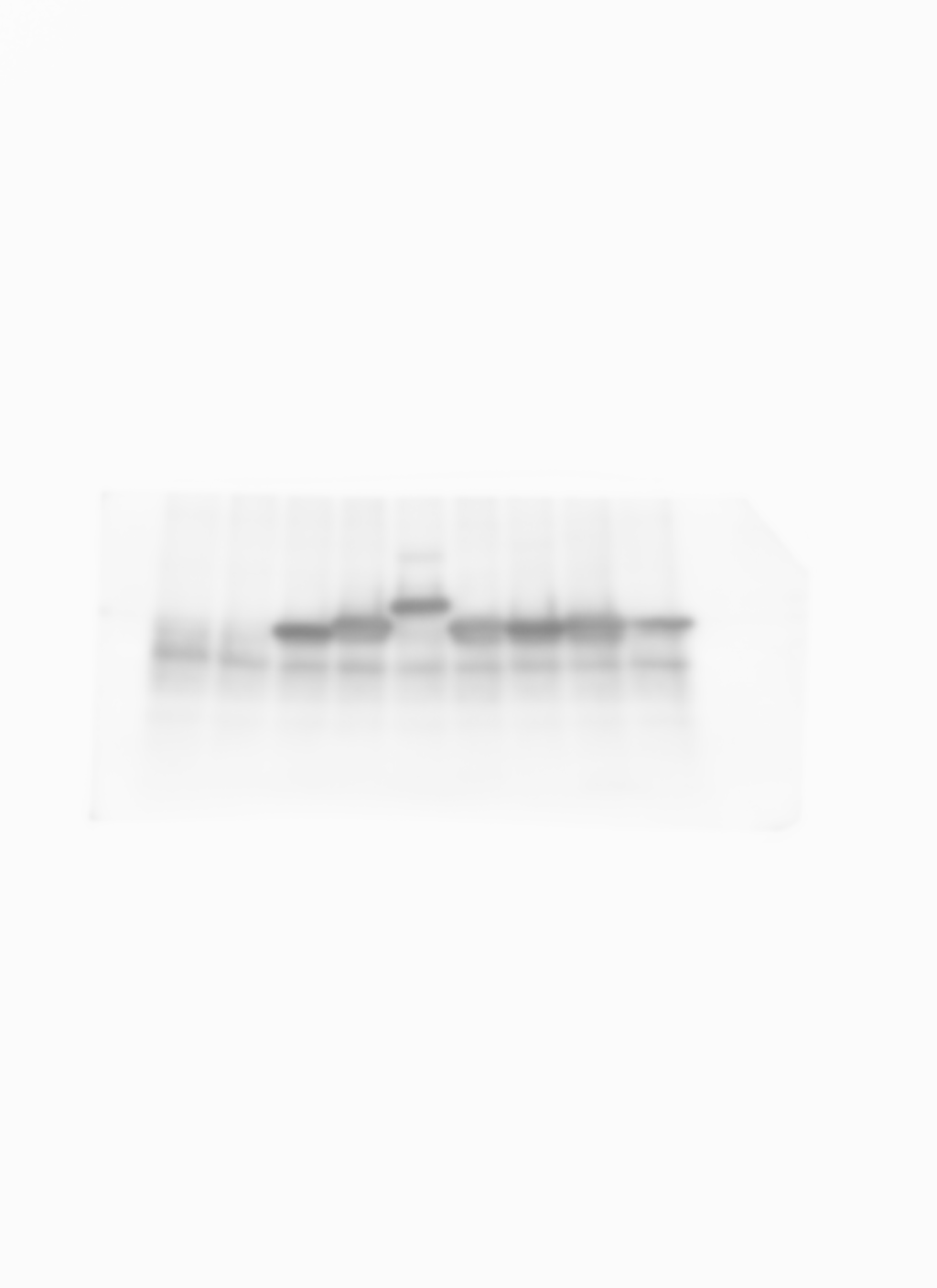

Supplement: Source data 1. [file elife-71966-data1.zip › Source Data Files/Raw Data/Figure 1-figure supplement 2/2, IB-HA (IP).tif]

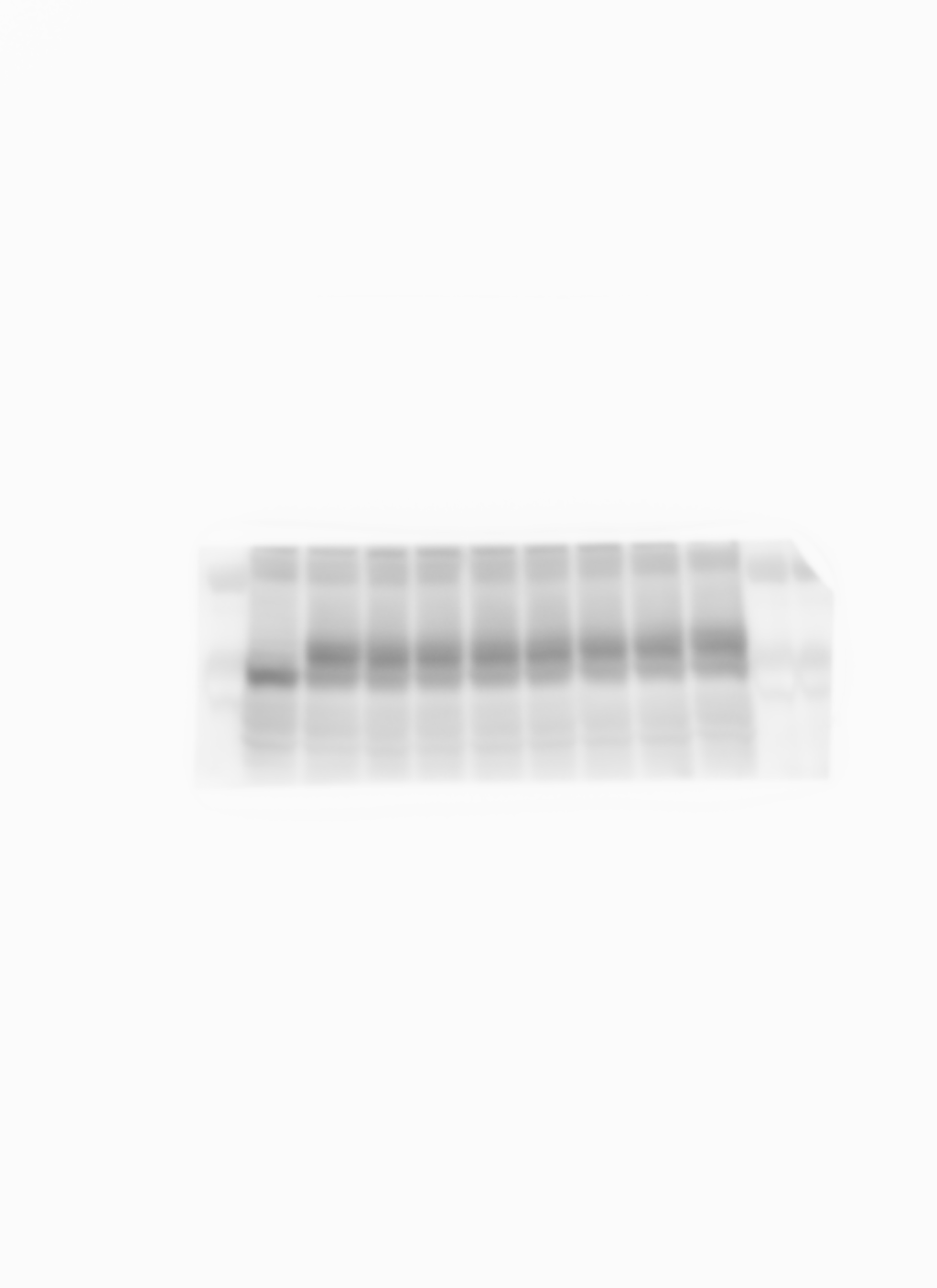

Supplement: Source data 1. [file elife-71966-data1.zip › Source Data Files/Raw Data/Figure 1-figure supplement 2/3, IB-Reg1 (Input).tif]

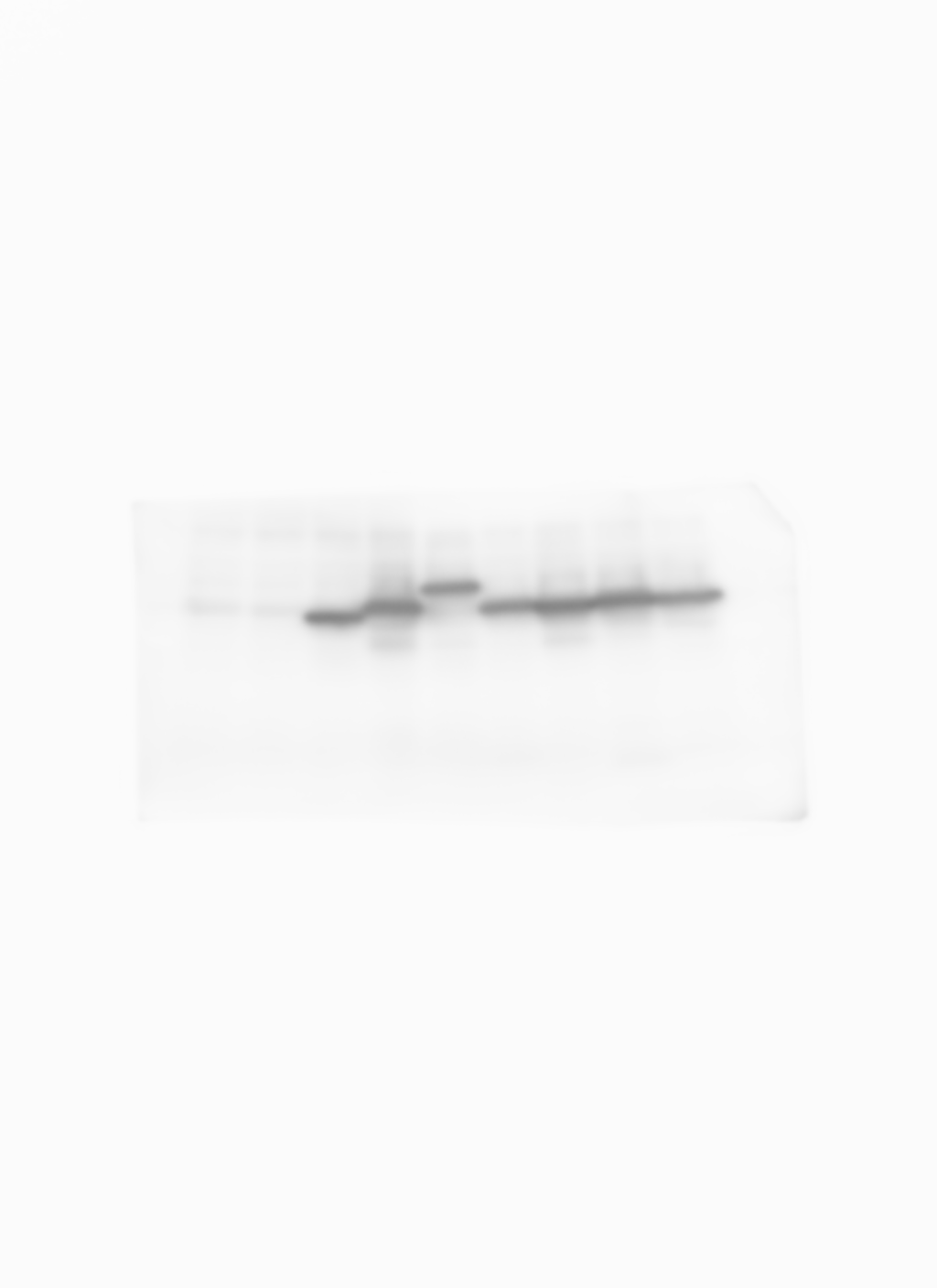

Supplement: Source data 1. [file elife-71966-data1.zip › Source Data Files/Raw Data/Figure 1-figure supplement 2/4, IB-HA (Input).tif]

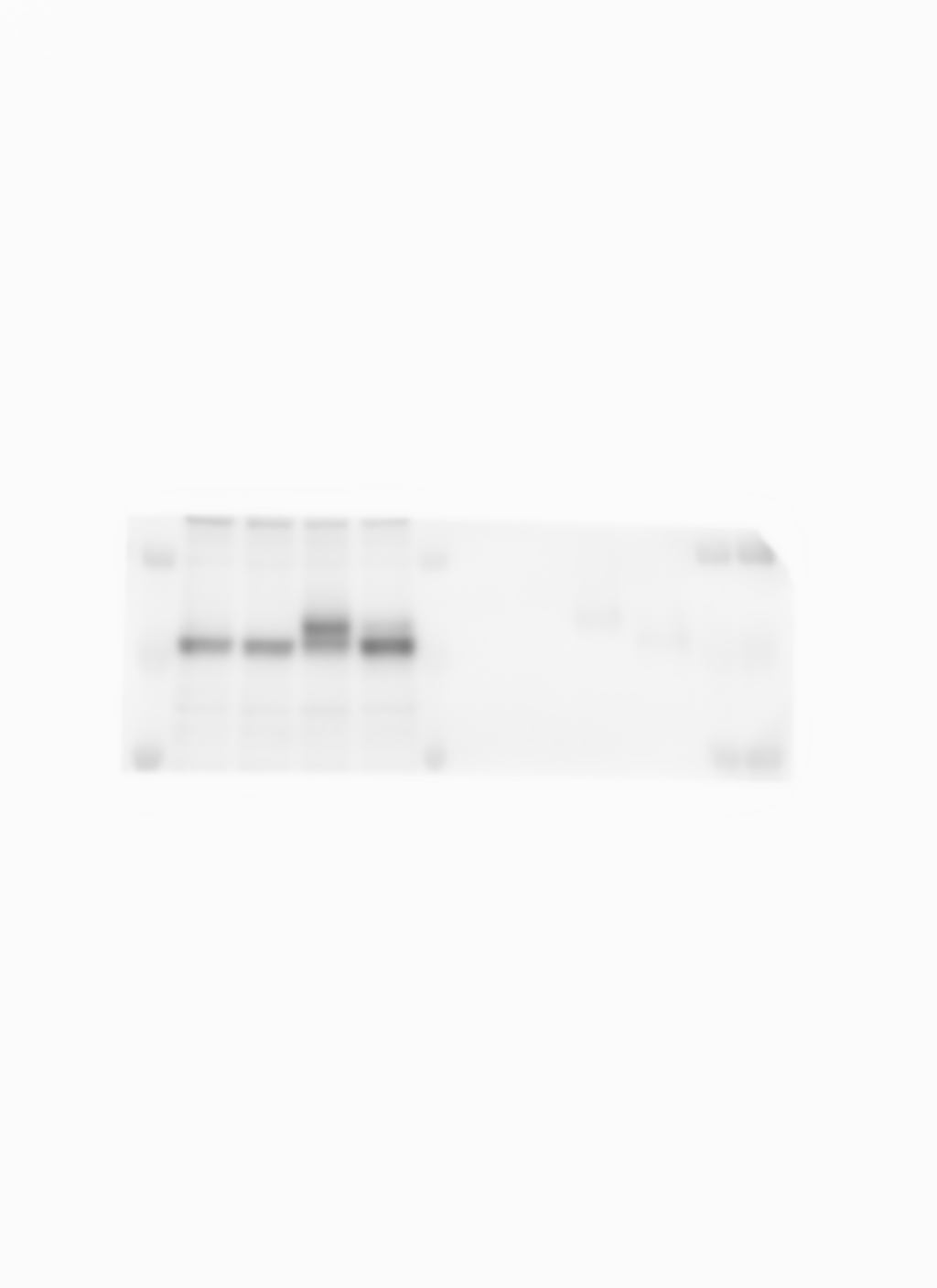

Supplement: Source data 1. [file elife-71966-data1.zip › Source Data Files/Raw Data/Figure 2-A/1, IB-Reg1.tif]

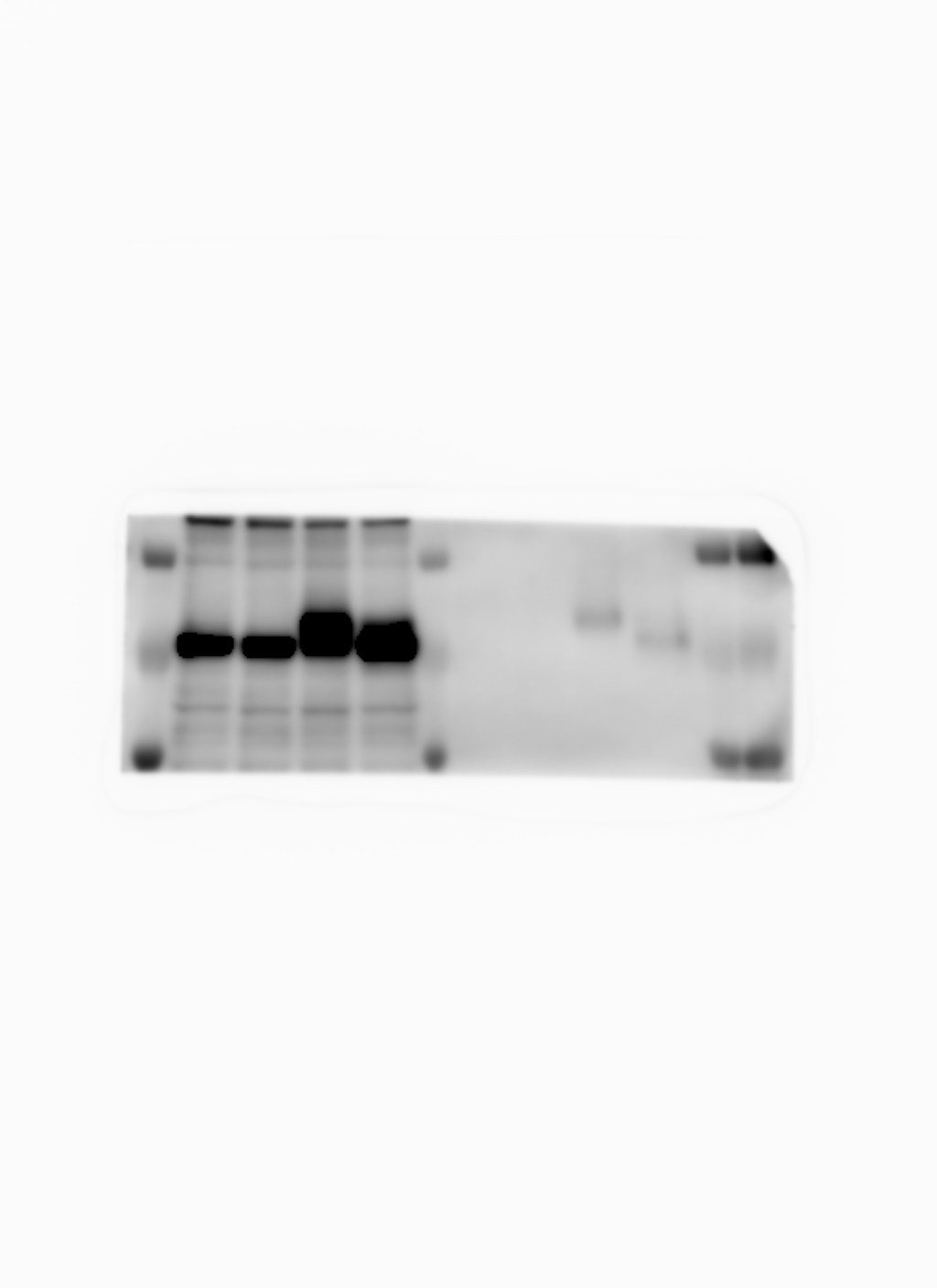

Supplement: Source data 1. [file elife-71966-data1.zip › Source Data Files/Raw Data/Figure 2-A/2, IB-Reg1 (Long exposure).tif]

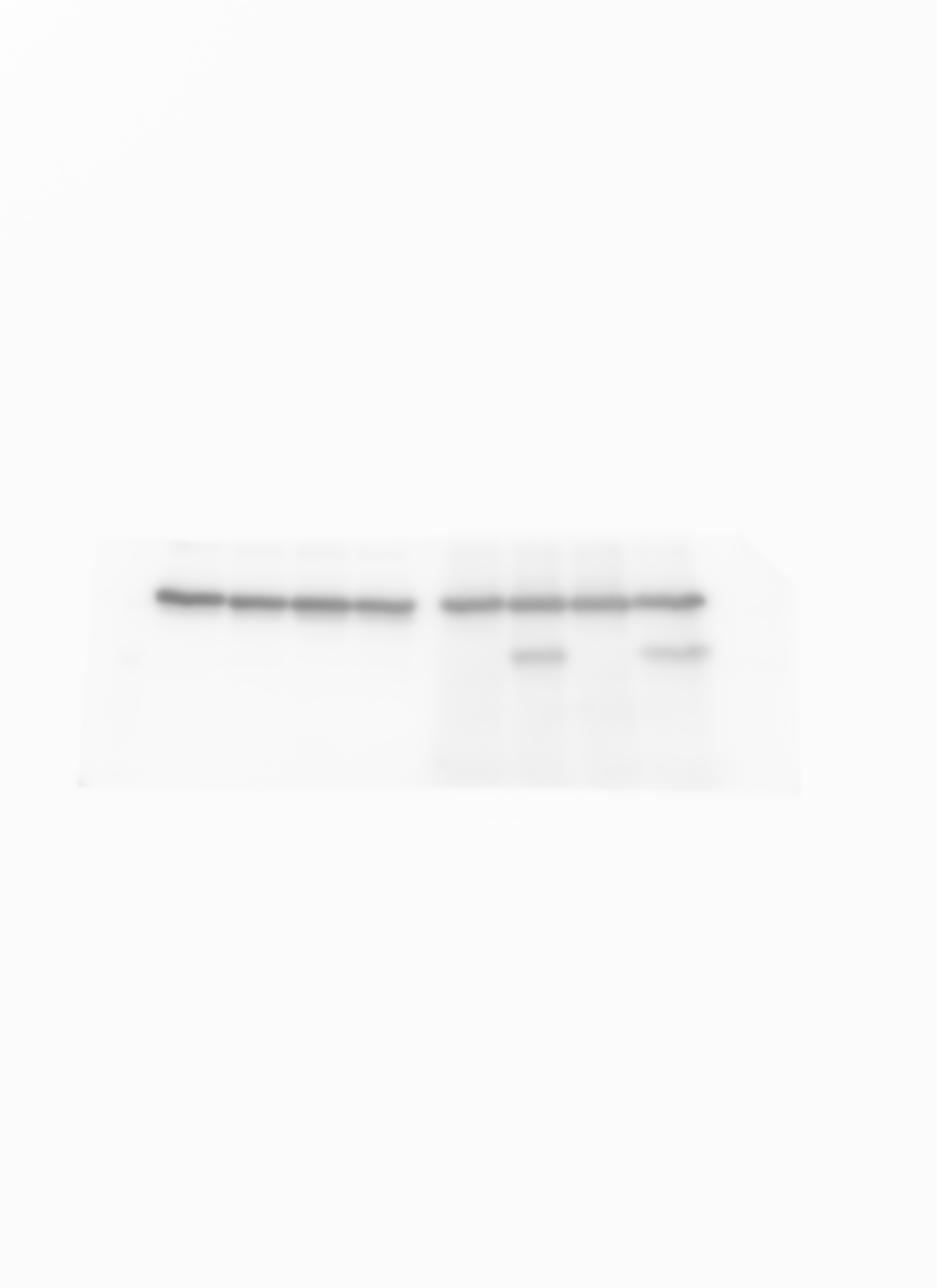

Supplement: Source data 1. [file elife-71966-data1.zip › Source Data Files/Raw Data/Figure 2-A/3, IB-HA.tif]

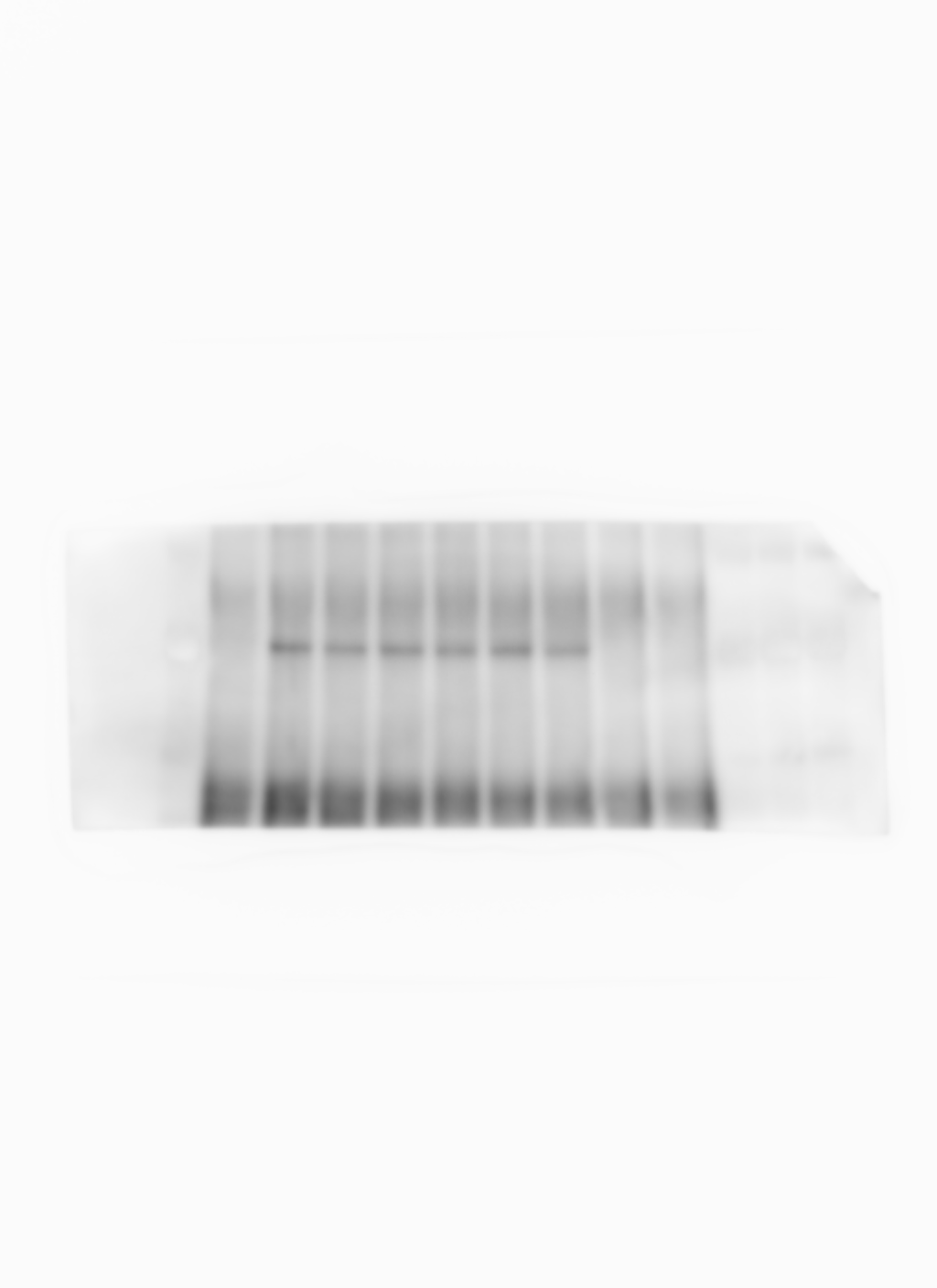

Supplement: Source data 1. [file elife-71966-data1.zip › Source Data Files/Raw Data/Figure 2-D/1, IB-FLAG (IP).tif]

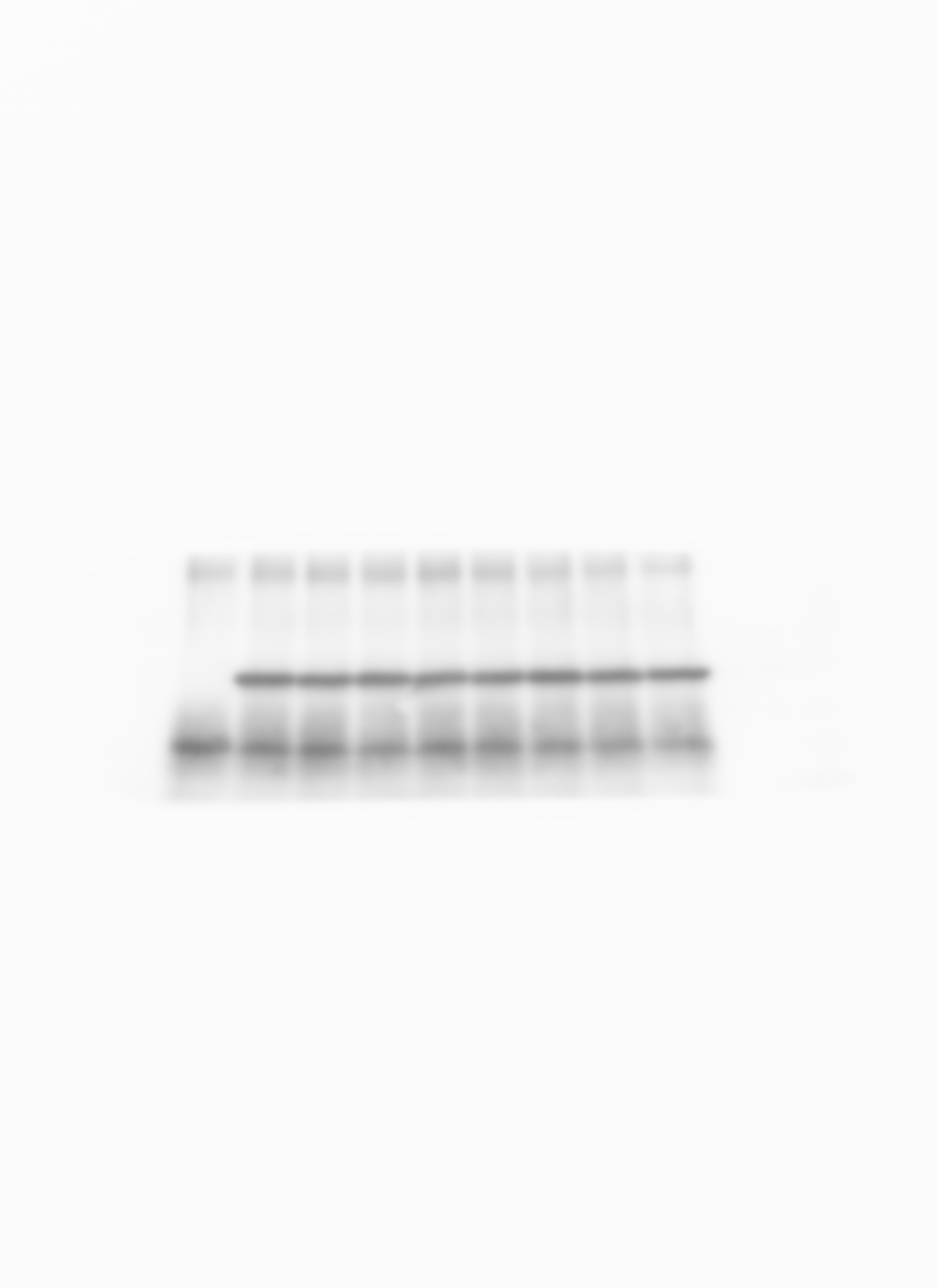

Supplement: Source data 1. [file elife-71966-data1.zip › Source Data Files/Raw Data/Figure 2-D/2, IB-HA (IP).tif]

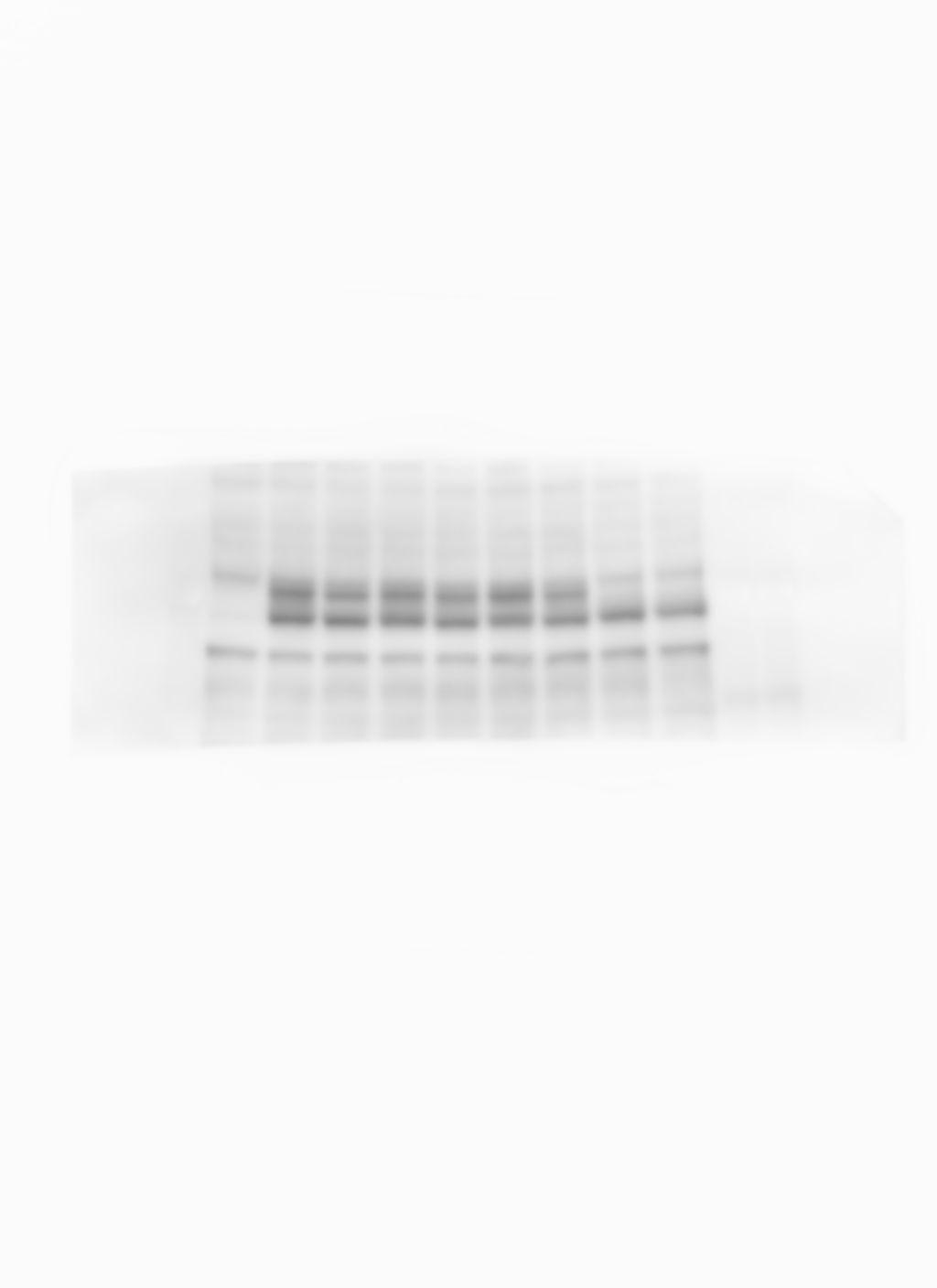

Supplement: Source data 1. [file elife-71966-data1.zip › Source Data Files/Raw Data/Figure 2-D/3, IB-FLAG (Input).tif]

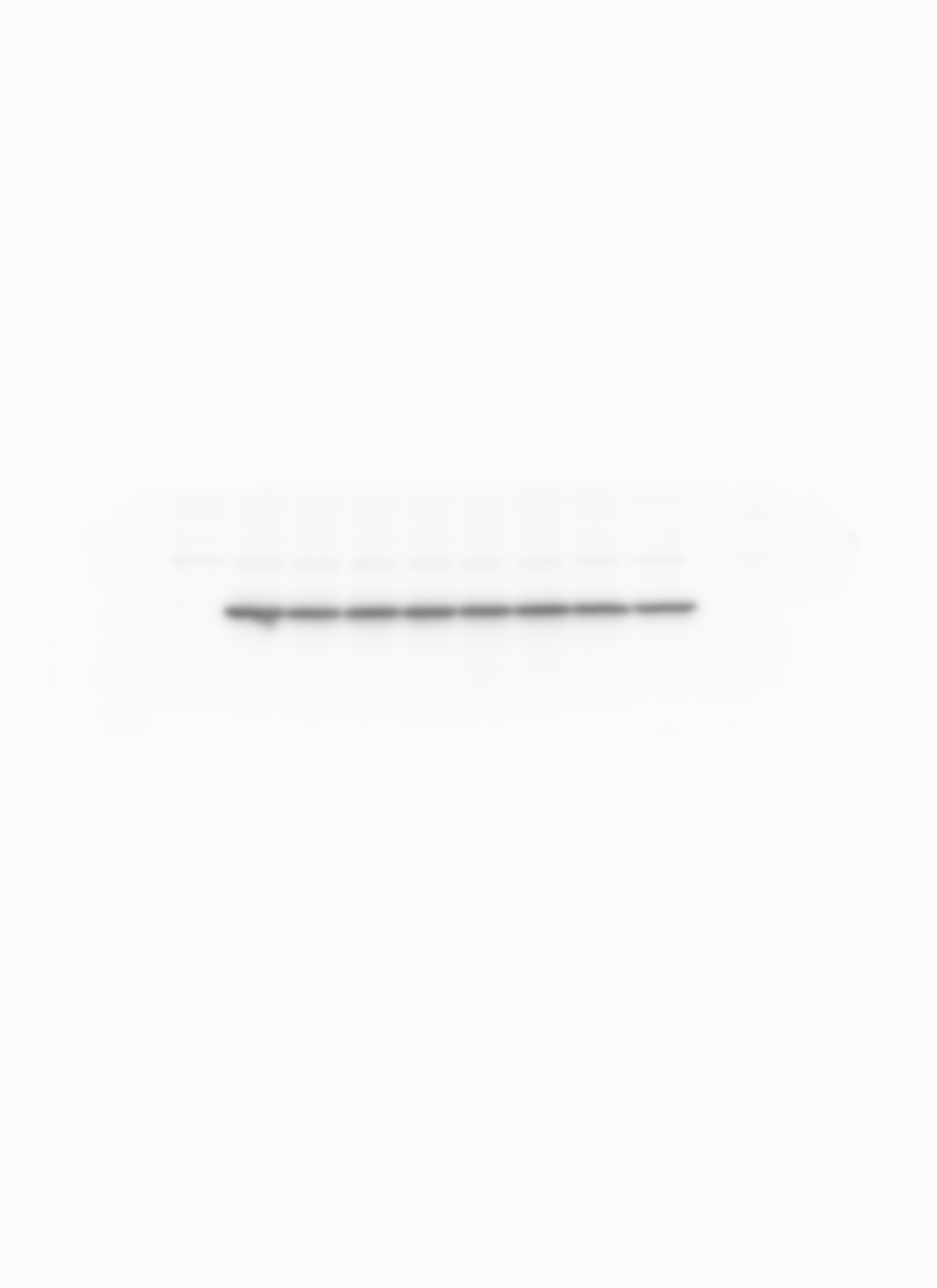

Supplement: Source data 1. [file elife-71966-data1.zip › Source Data Files/Raw Data/Figure 2-D/4, IB-HA (Input).tif]

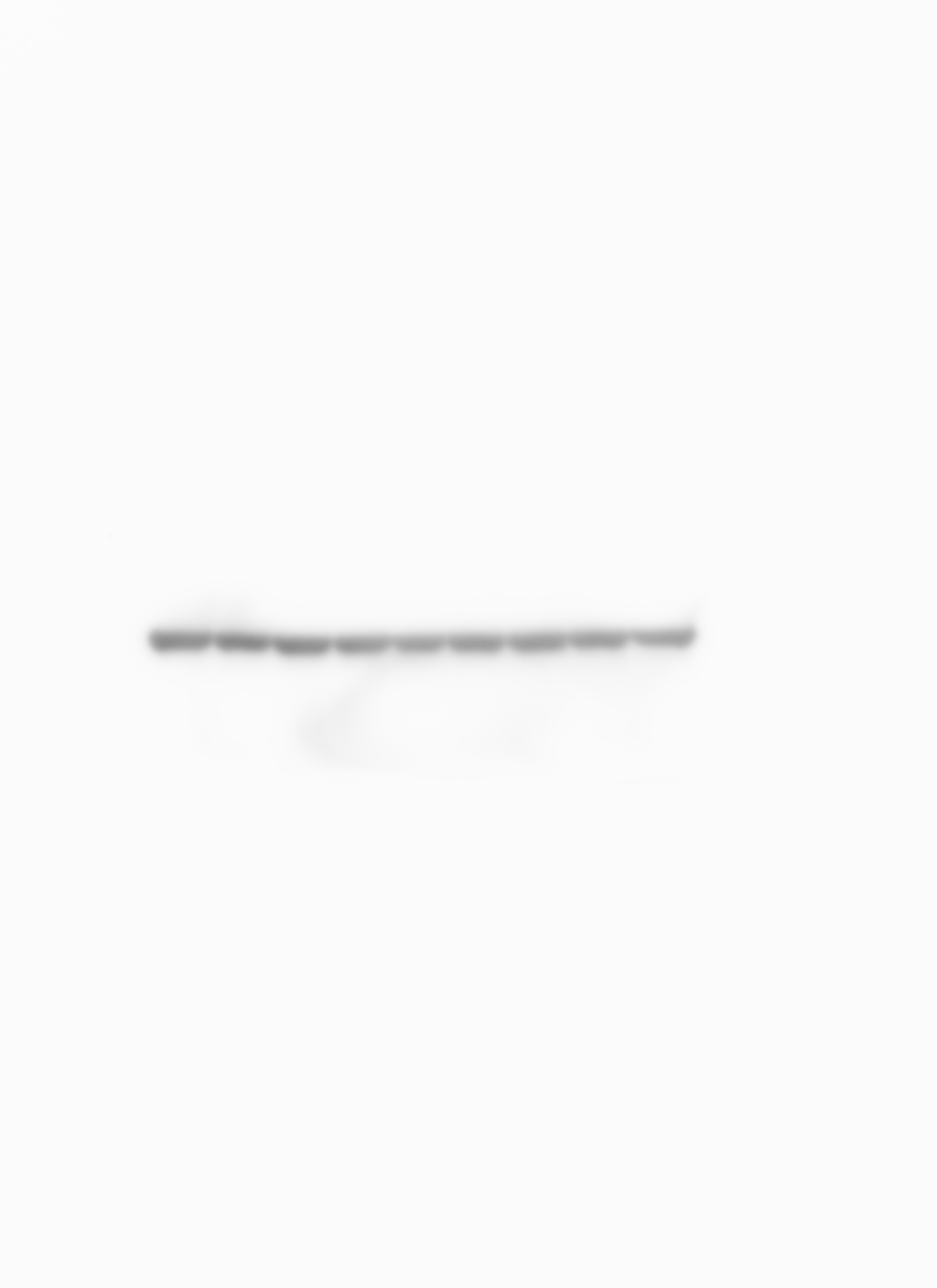

Supplement: Source data 1. [file elife-71966-data1.zip › Source Data Files/Raw Data/Figure 2-D/5, IB-Actin (Input).tif]

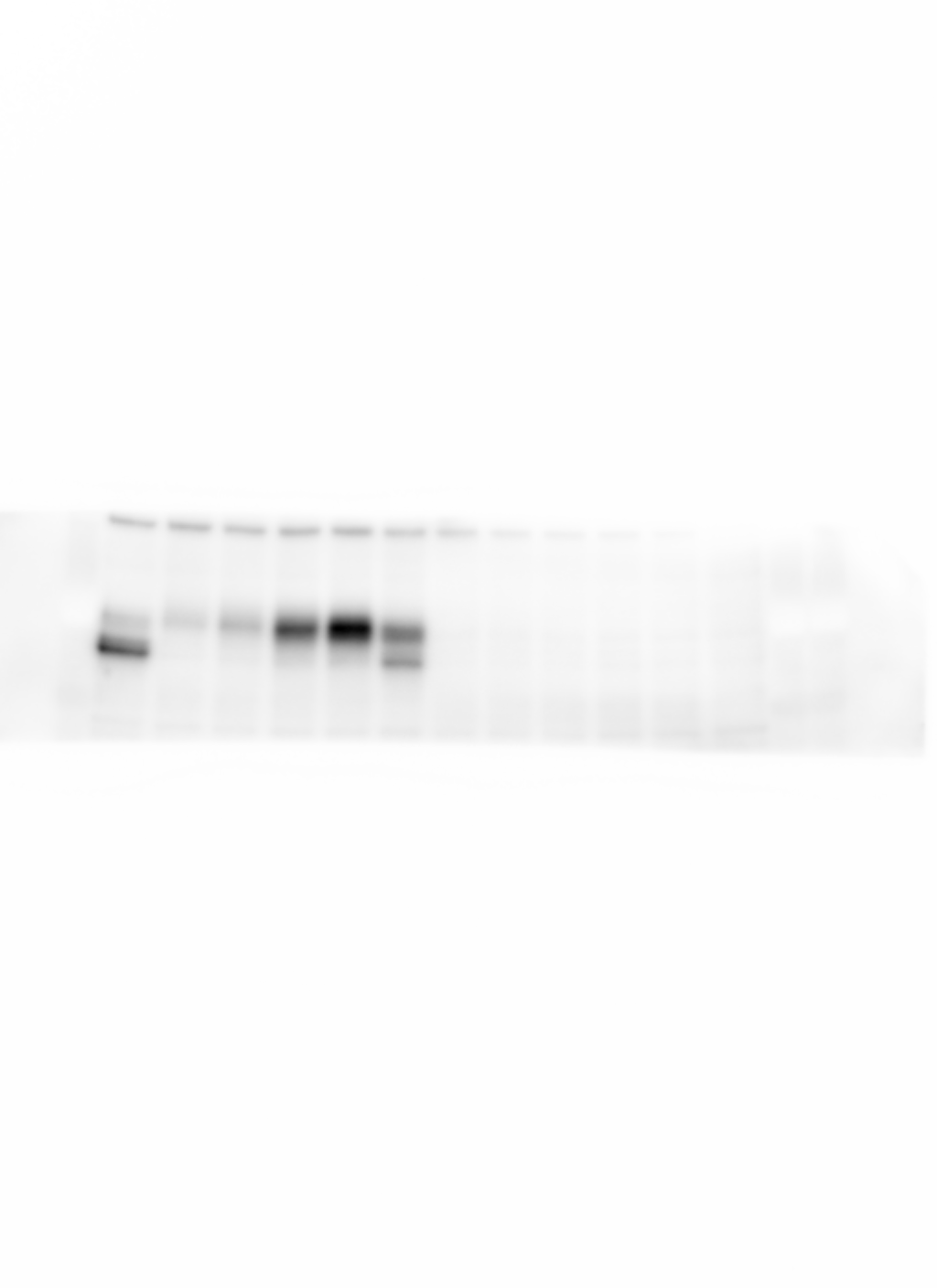

Supplement: Source data 1. [file elife-71966-data1.zip › Source Data Files/Raw Data/Figure 2-figure supplement 1/1, IB-Reg1.tif]

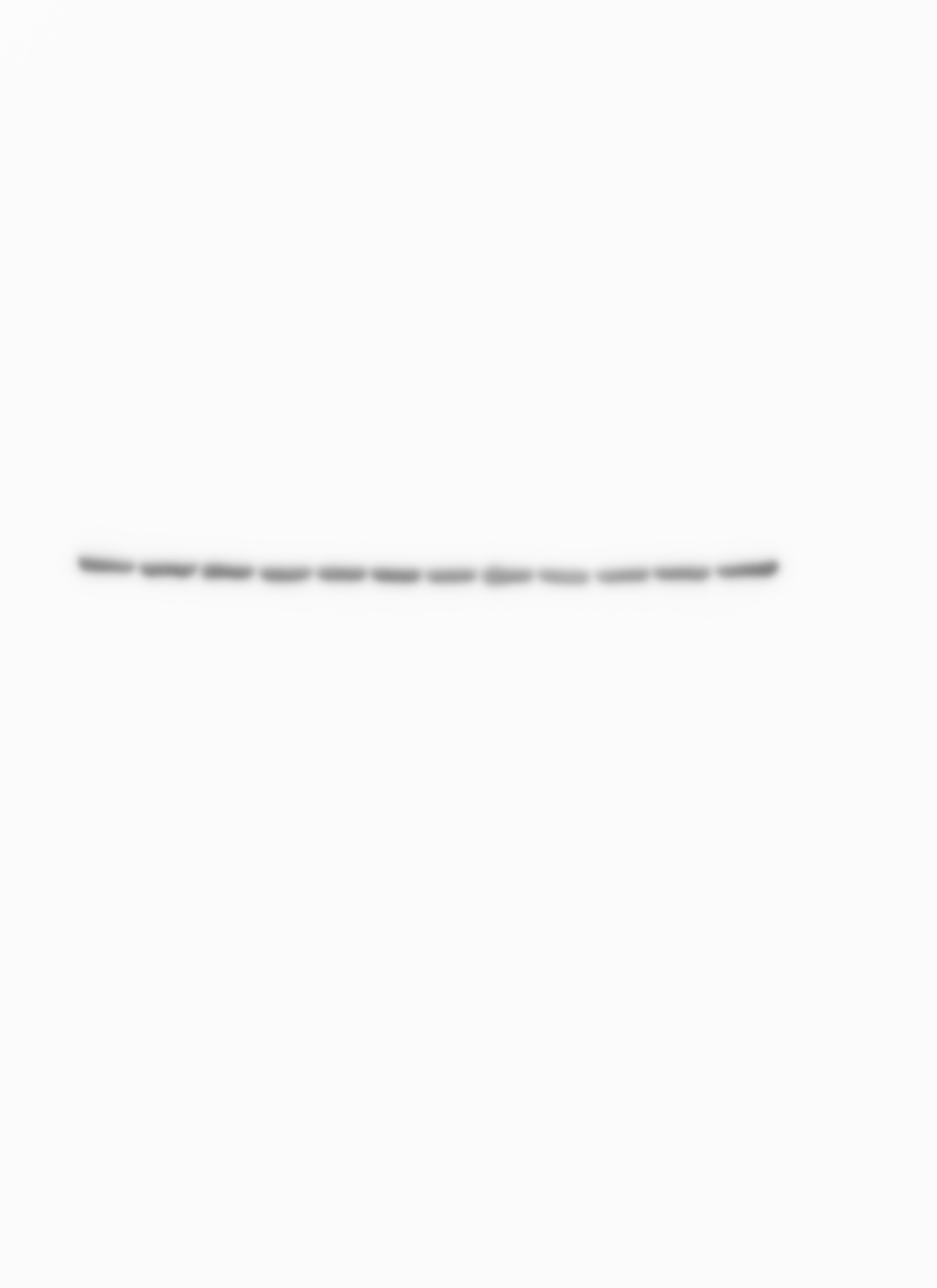

Supplement: Source data 1. [file elife-71966-data1.zip › Source Data Files/Raw Data/Figure 2-figure supplement 1/2, IB-Actin.tif]

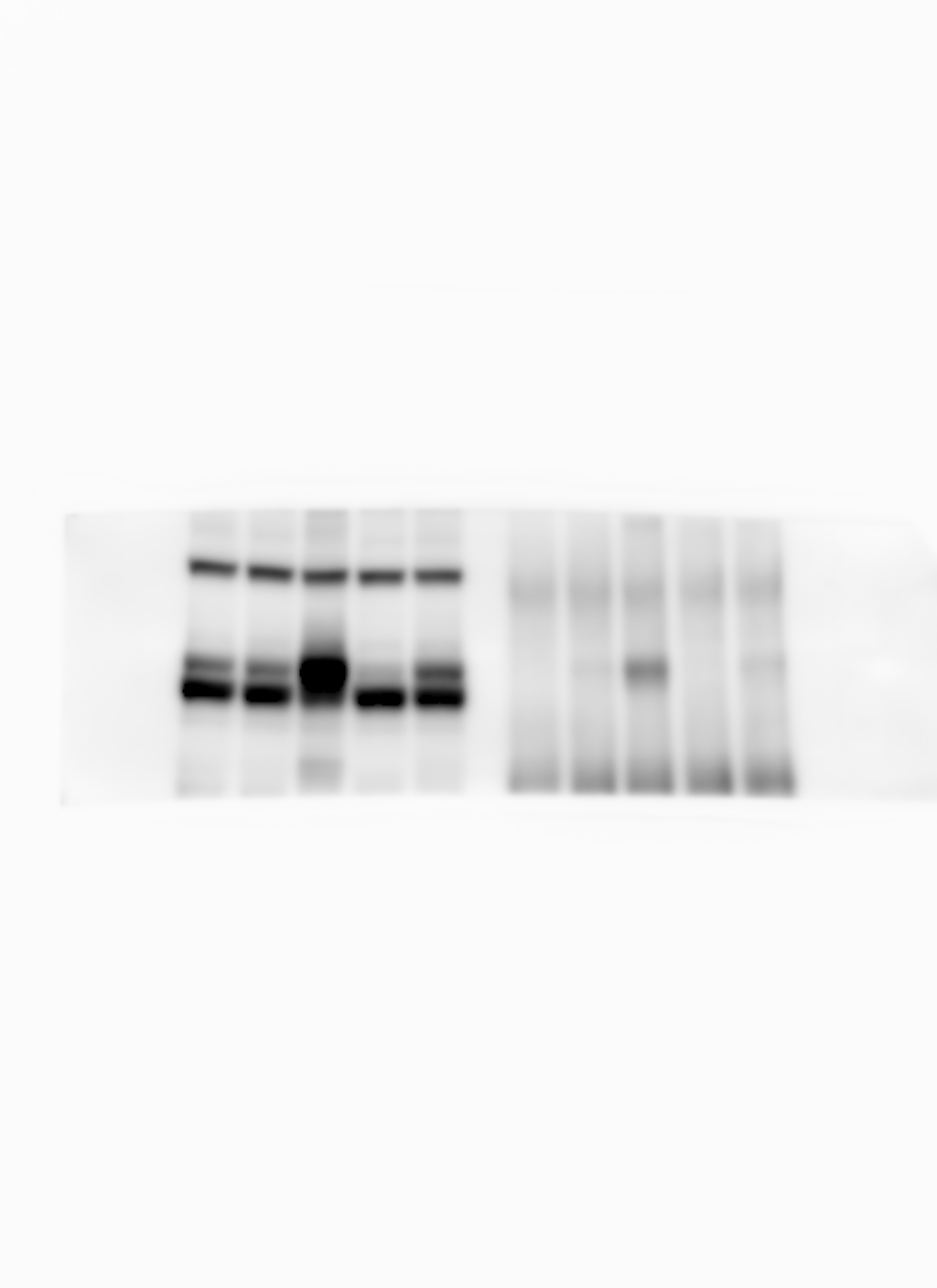

Supplement: Source data 1. [file elife-71966-data1.zip › Source Data Files/Raw Data/Figure 2-figure supplement 3/1, IB-Reg1 (LE).tif]

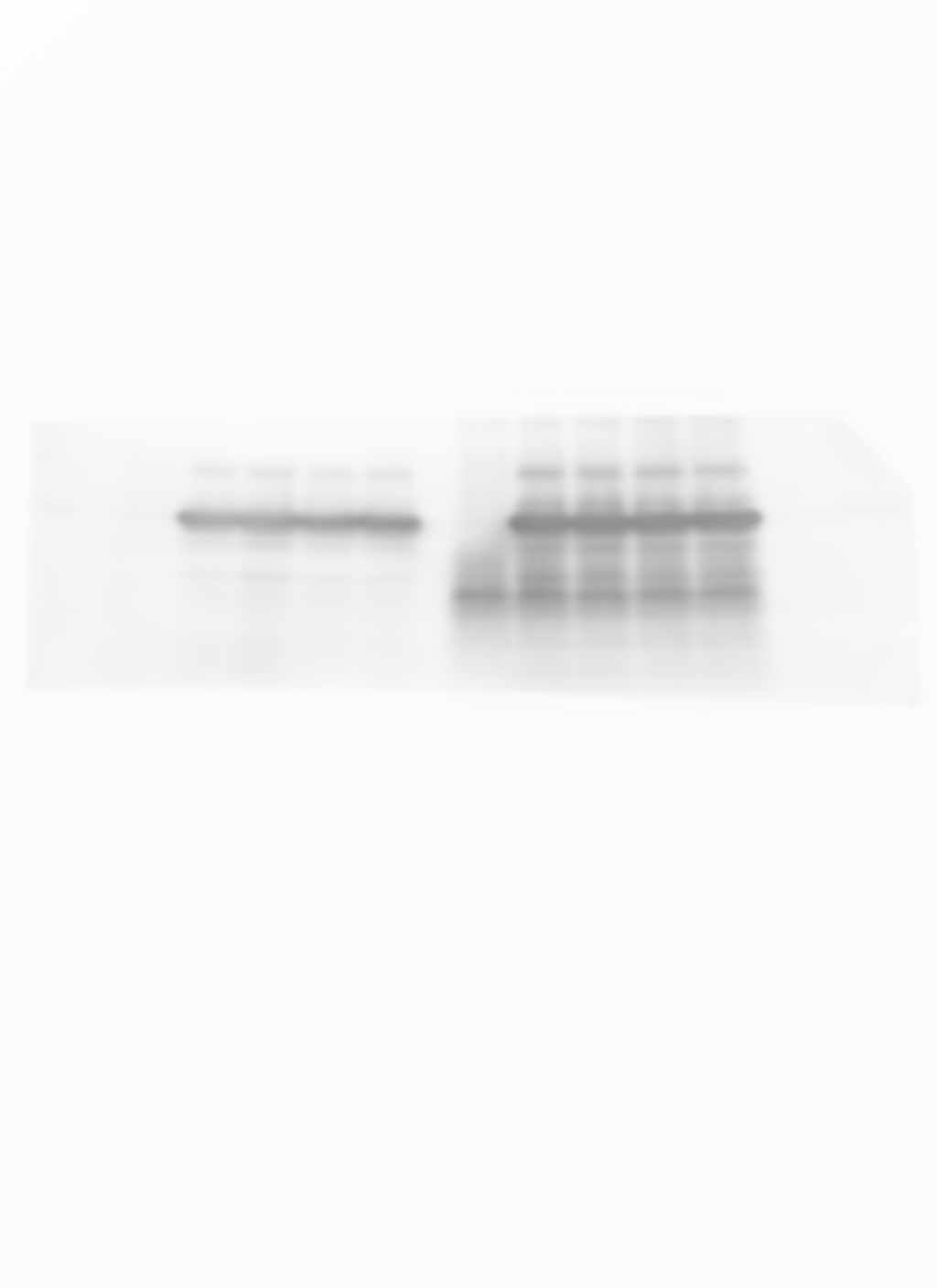

Supplement: Source data 1. [file elife-71966-data1.zip › Source Data Files/Raw Data/Figure 2-figure supplement 3/2 and 4, IB-HA (Input and IP).tif]

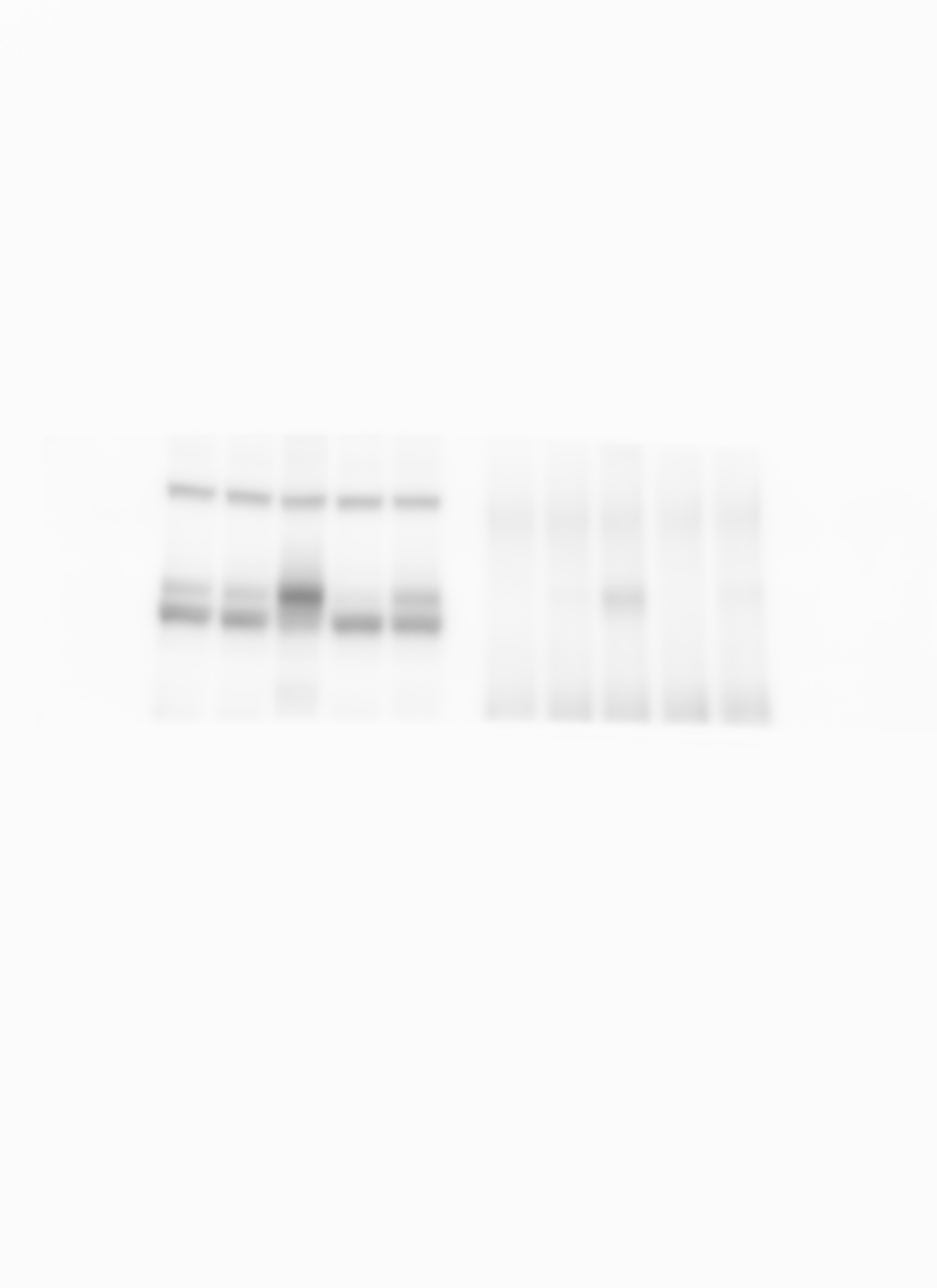

Supplement: Source data 1. [file elife-71966-data1.zip › Source Data Files/Raw Data/Figure 2-figure supplement 3/3, IB-Reg1 (Input).tif]

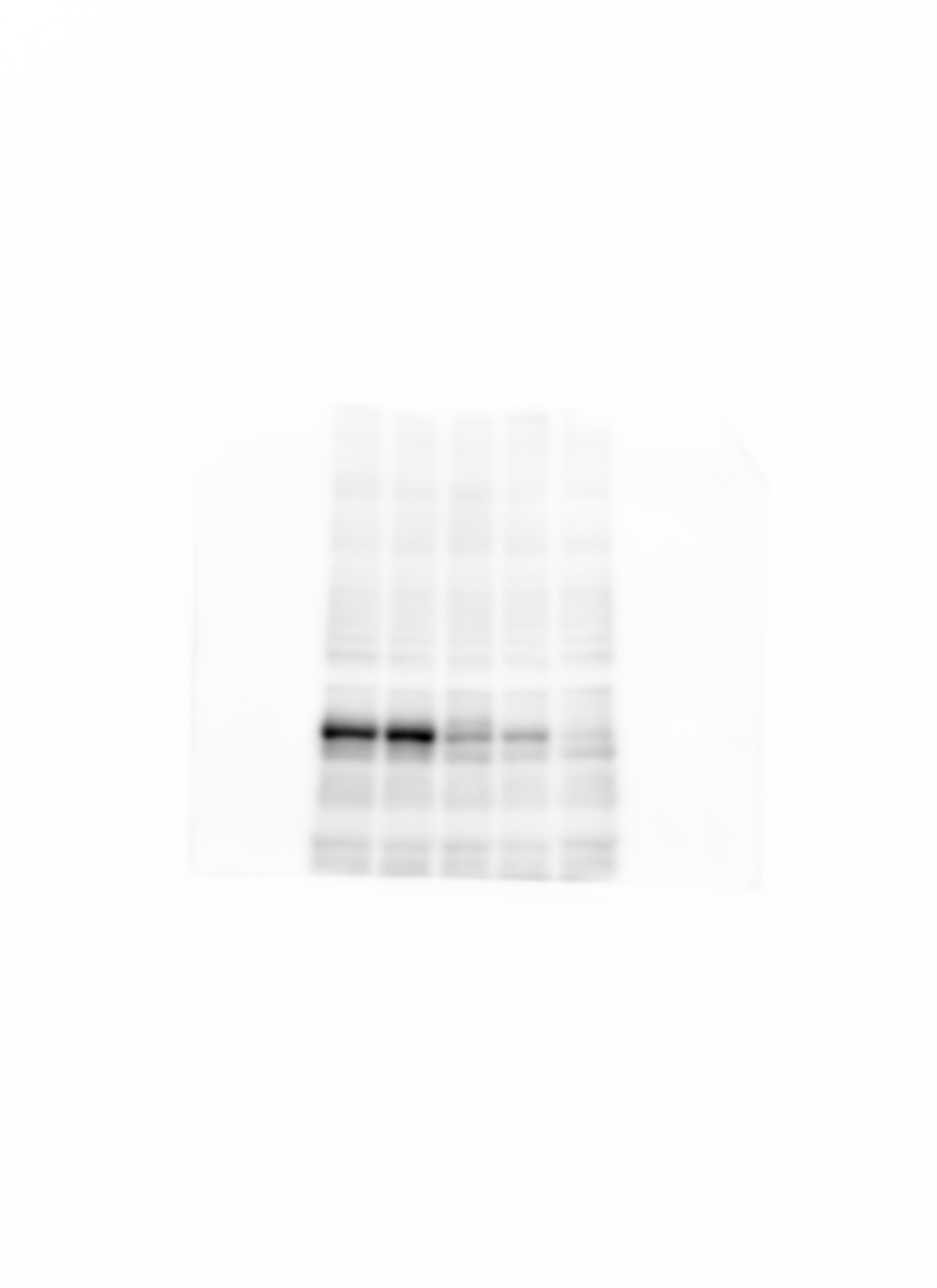

Supplement: Source data 1. [file elife-71966-data1.zip › Source Data Files/Raw Data/Figure 2-figure supplement 3/5, IB-IRAK1 (Input).tif]

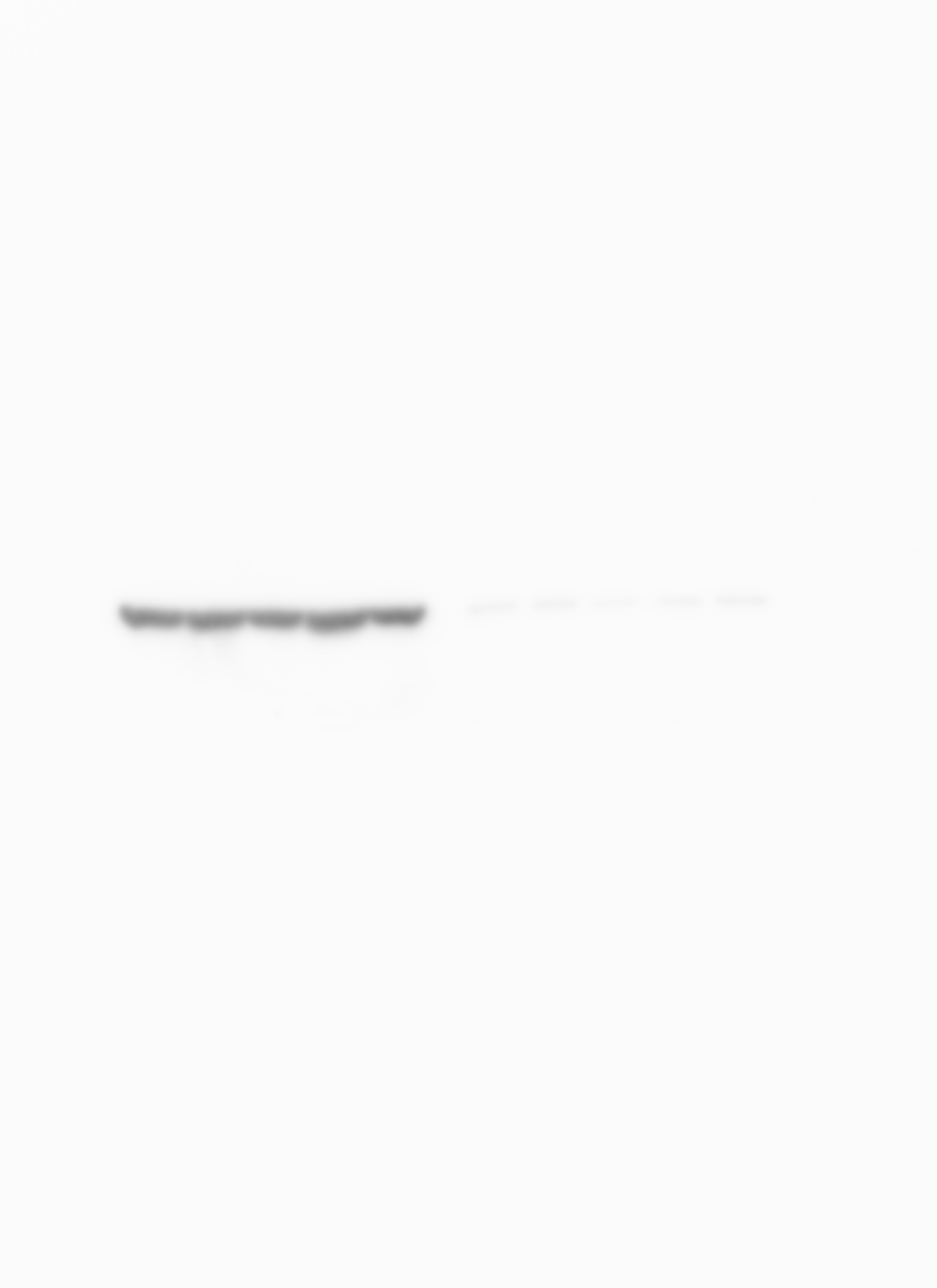

Supplement: Source data 1. [file elife-71966-data1.zip › Source Data Files/Raw Data/Figure 2-figure supplement 3/6, IB-Actin (Input).tif]

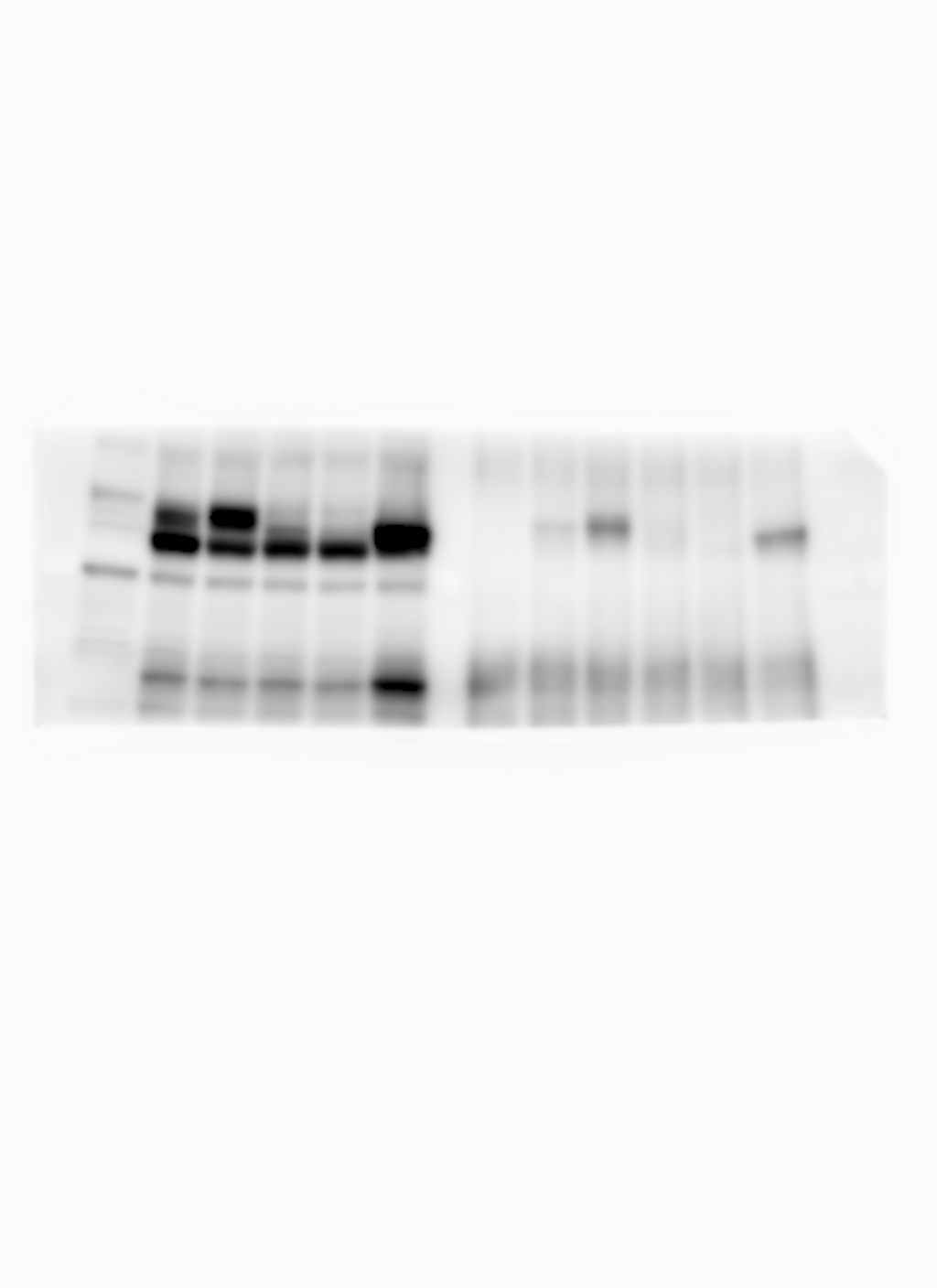

Supplement: Source data 1. [file elife-71966-data1.zip › Source Data Files/Raw Data/Figure 2-figure supplement 8/1 and 3, IB-FLAG (IP and Input).tif]

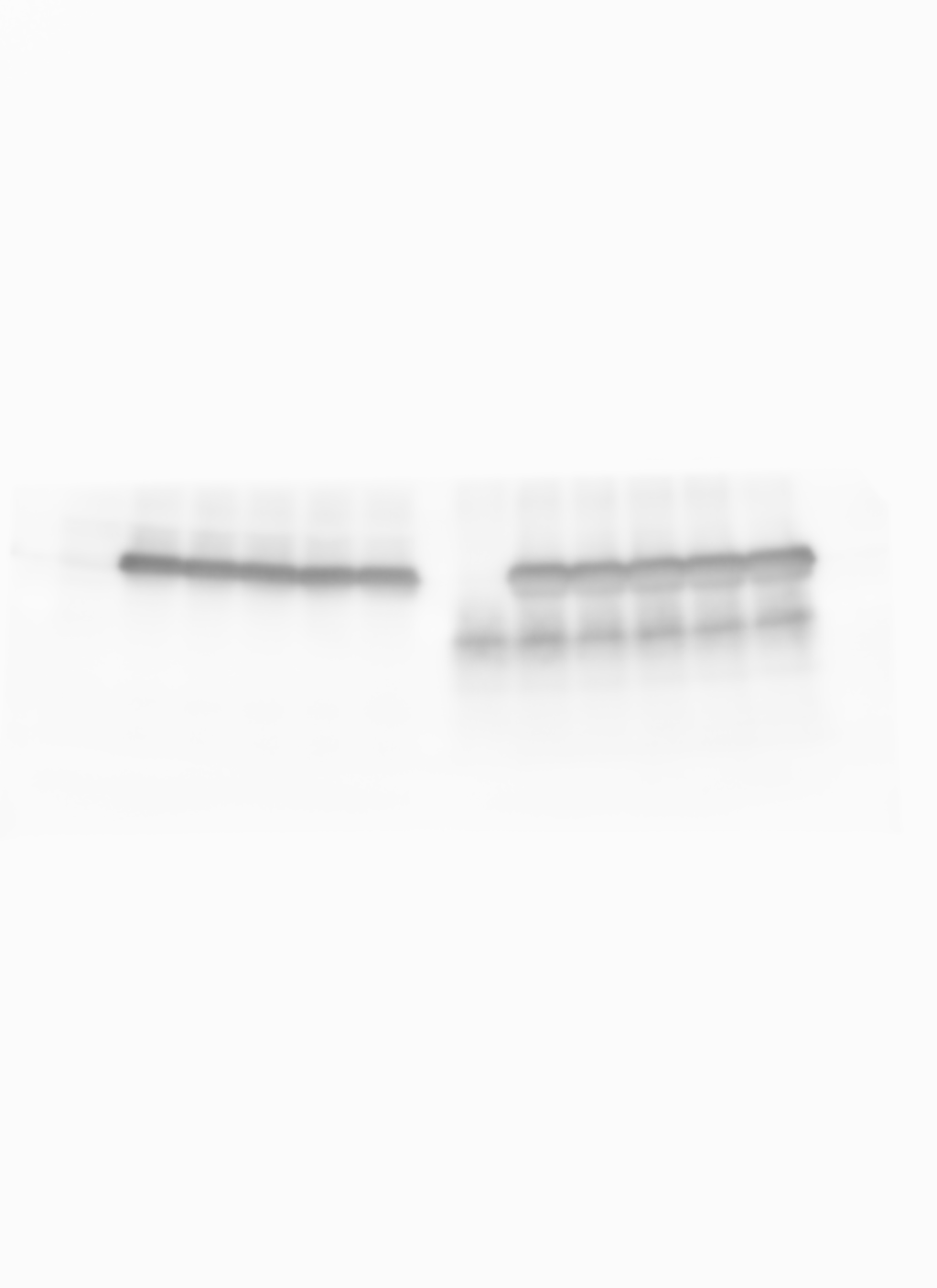

Supplement: Source data 1. [file elife-71966-data1.zip › Source Data Files/Raw Data/Figure 2-figure supplement 8/2 and 4, IB-HA (IP and Input).tif]

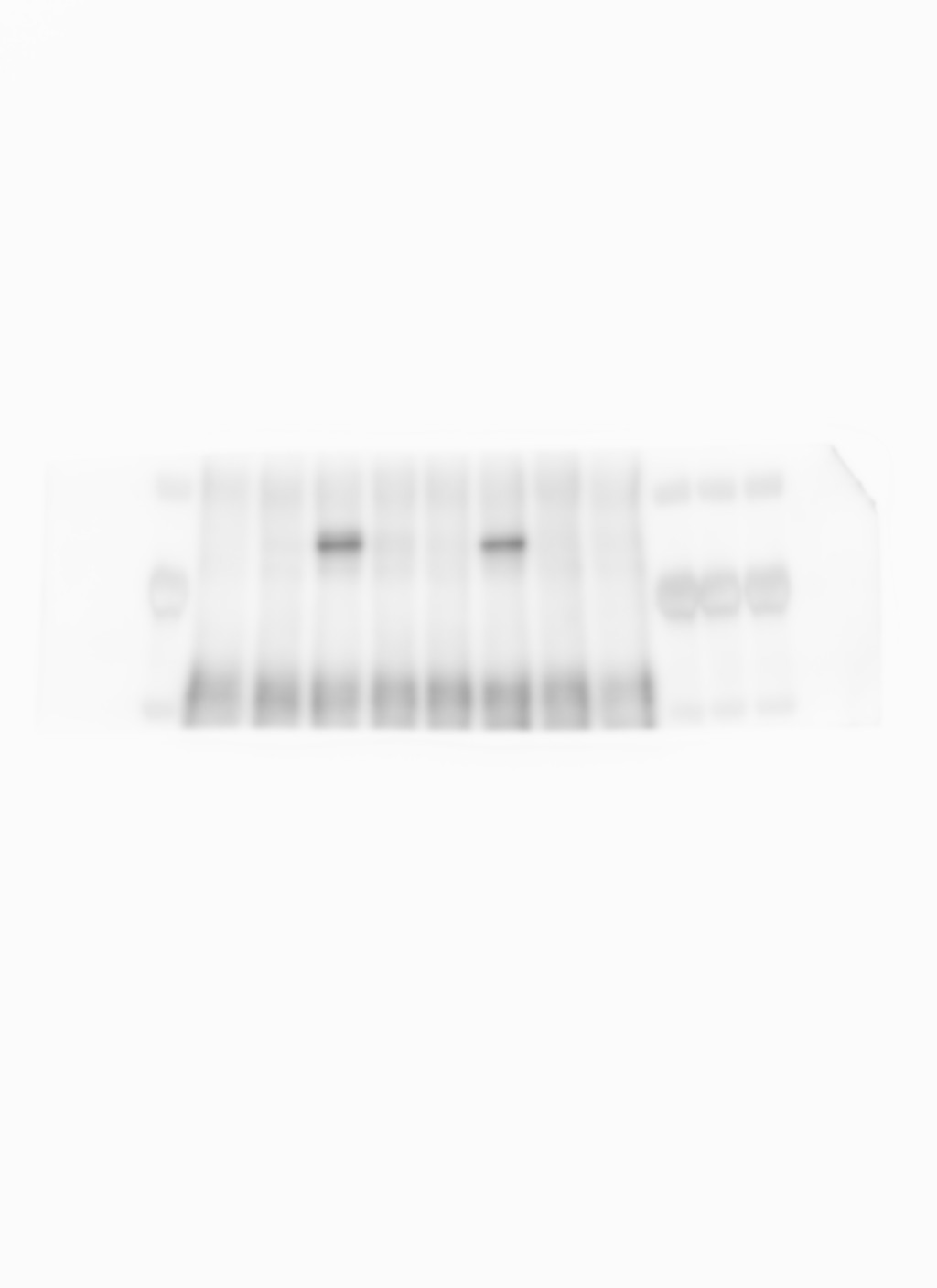

Supplement: Source data 1. [file elife-71966-data1.zip › Source Data Files/Raw Data/Figure 2-G/1, IB-FLAG (IP).tif]

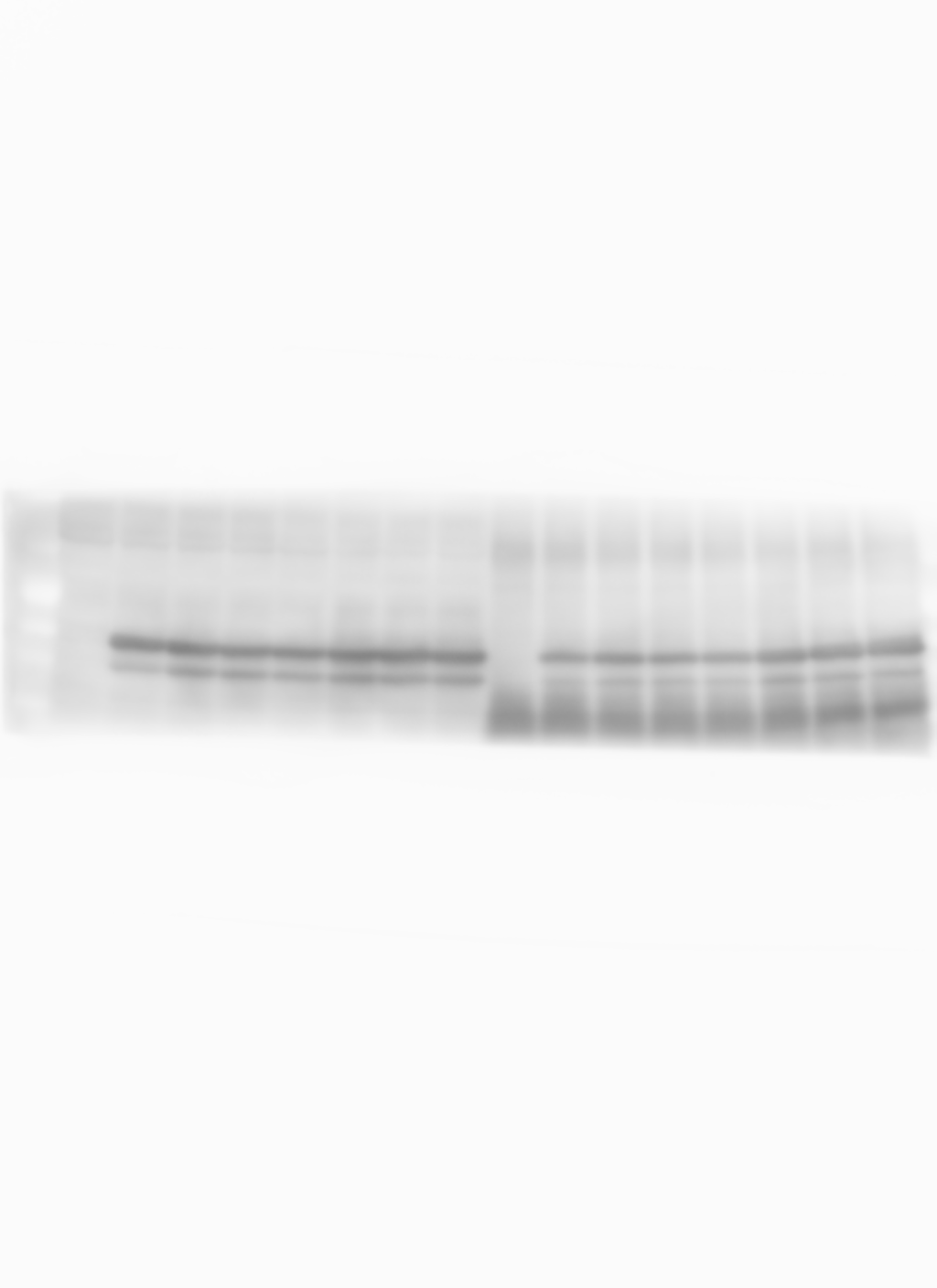

Supplement: Source data 1. [file elife-71966-data1.zip › Source Data Files/Raw Data/Figure 2-G/2 and 5, IB-Myc (IP and Input).tif]

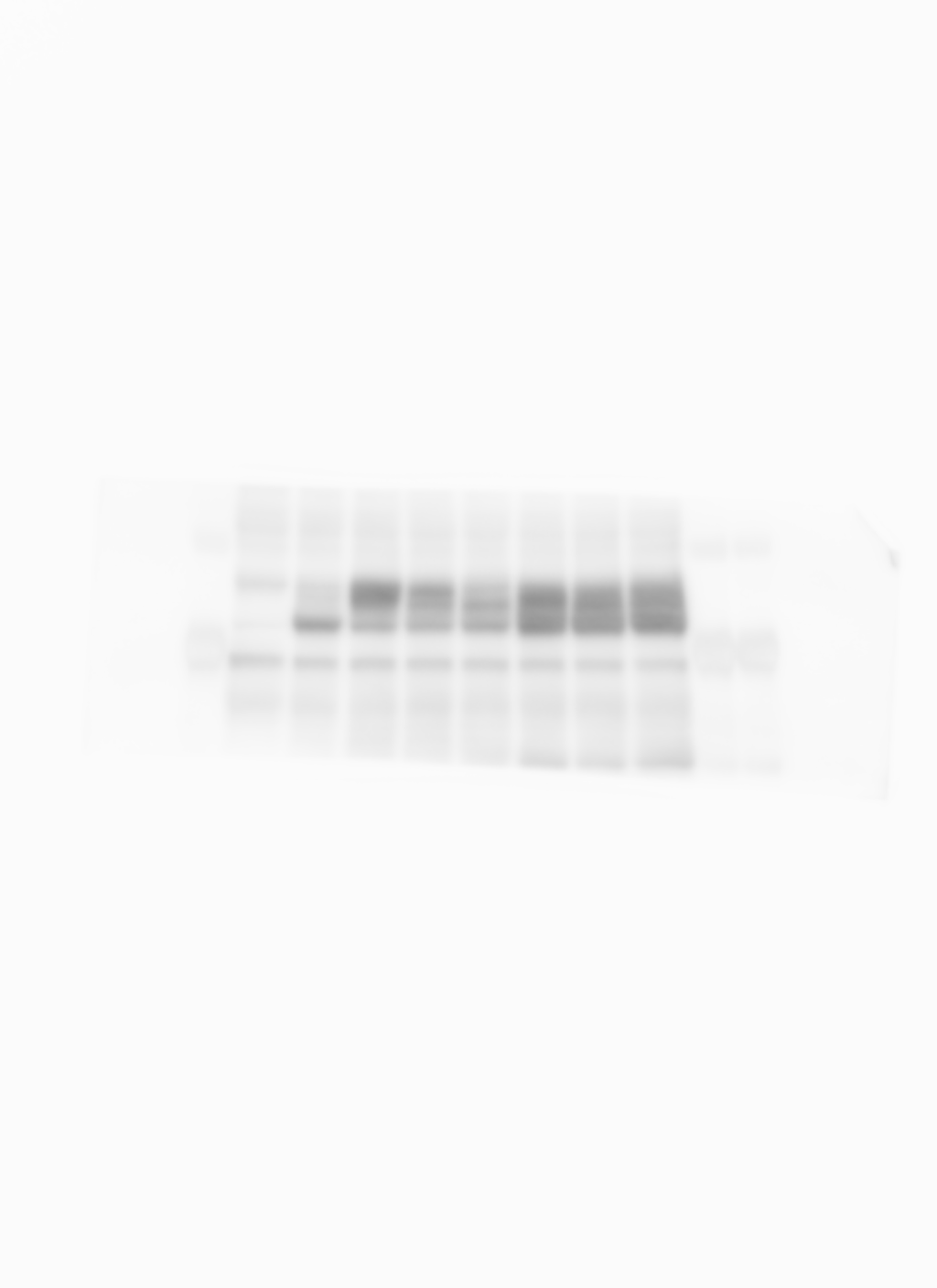

Supplement: Source data 1. [file elife-71966-data1.zip › Source Data Files/Raw Data/Figure 2-G/3, IB-FLAG (Input).tif]

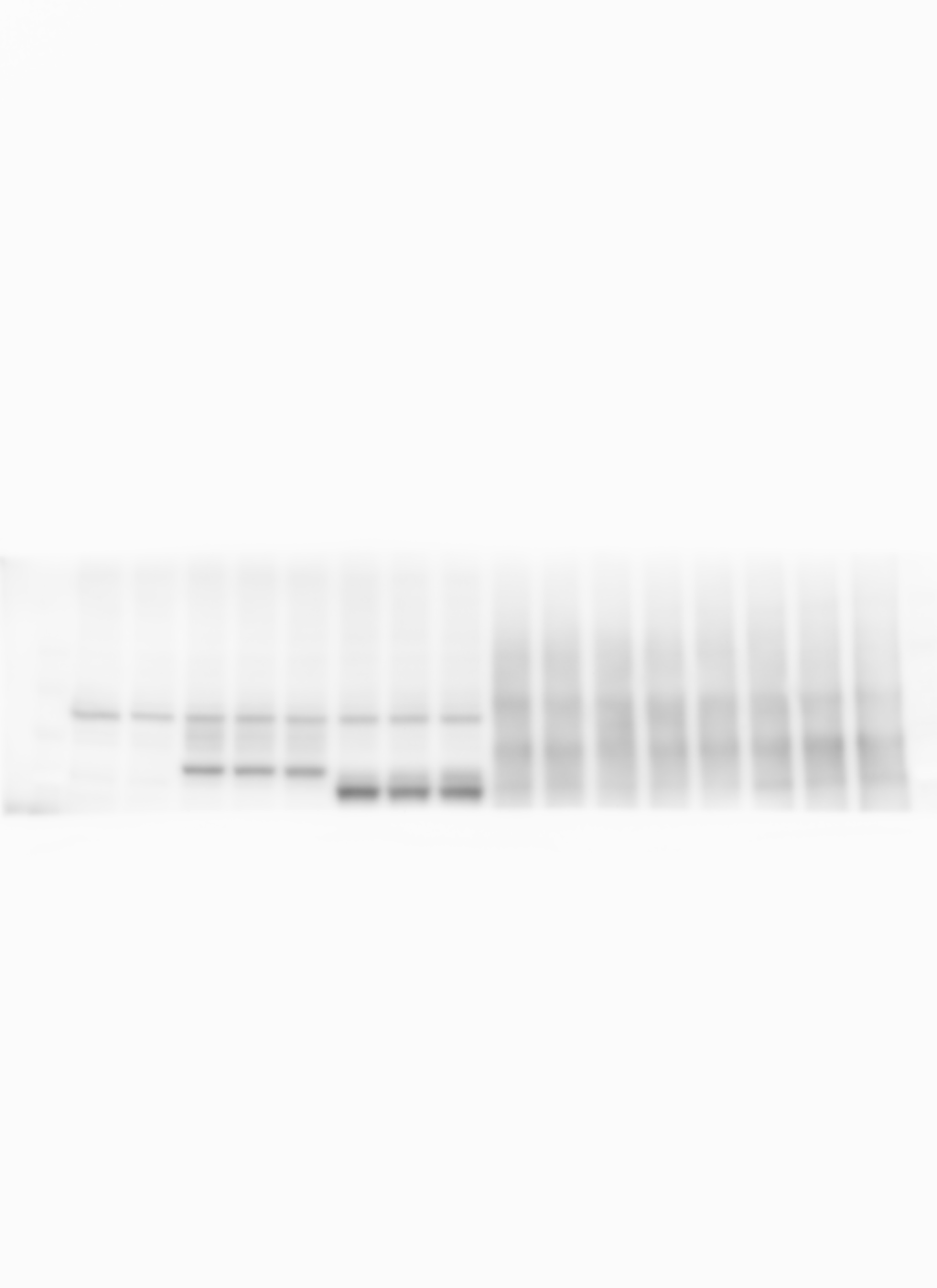

Supplement: Source data 1. [file elife-71966-data1.zip › Source Data Files/Raw Data/Figure 2-G/4, IB-HA (Input).tif]

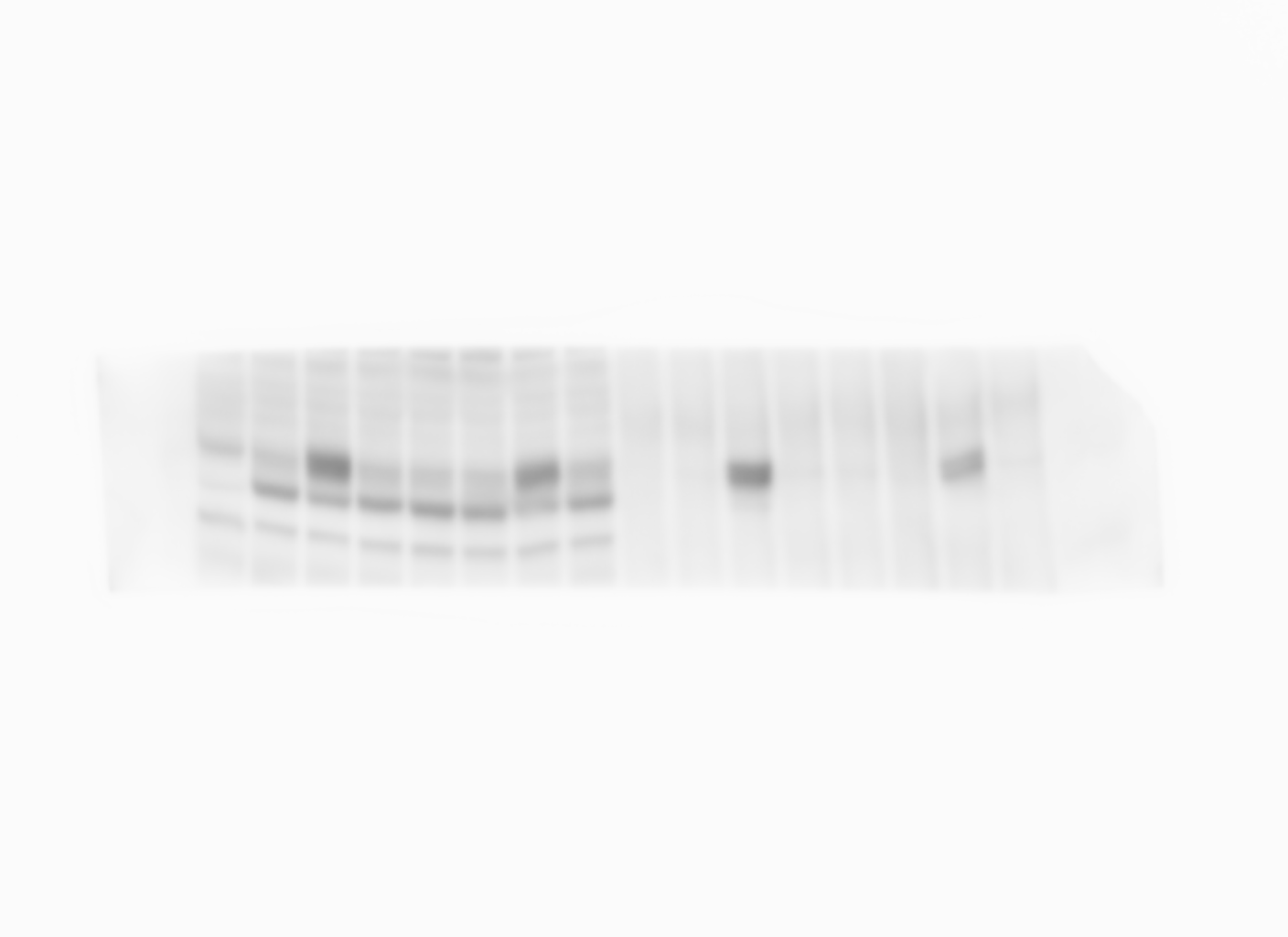

Supplement: Source data 1. [file elife-71966-data1.zip › Source Data Files/Raw Data/Figure 2-I/1 and 3, IB-FLAG (IP and Input).tif]

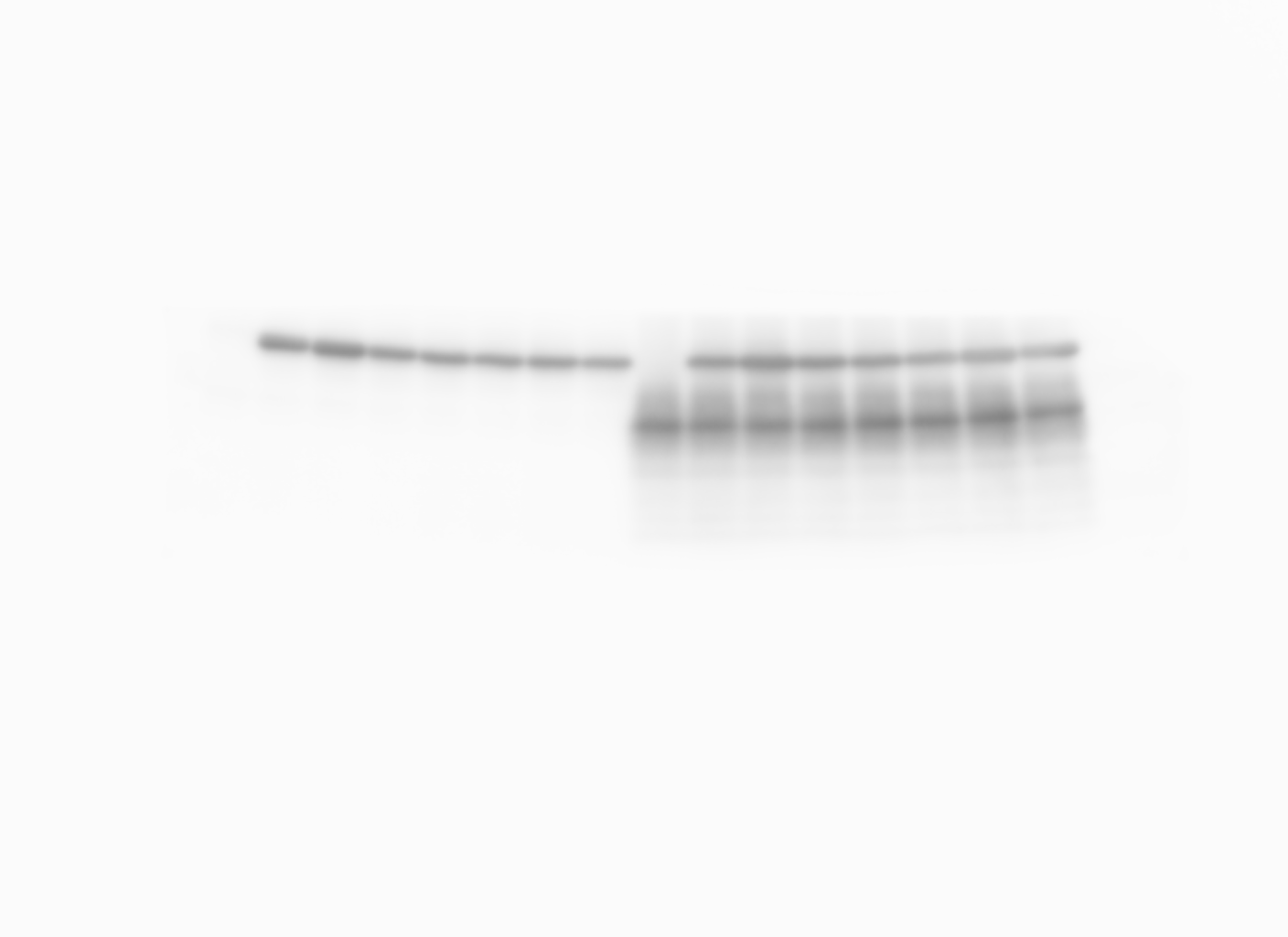

Supplement: Source data 1. [file elife-71966-data1.zip › Source Data Files/Raw Data/Figure 2-I/2 and 4, IB-HA (IP and Input).tif]

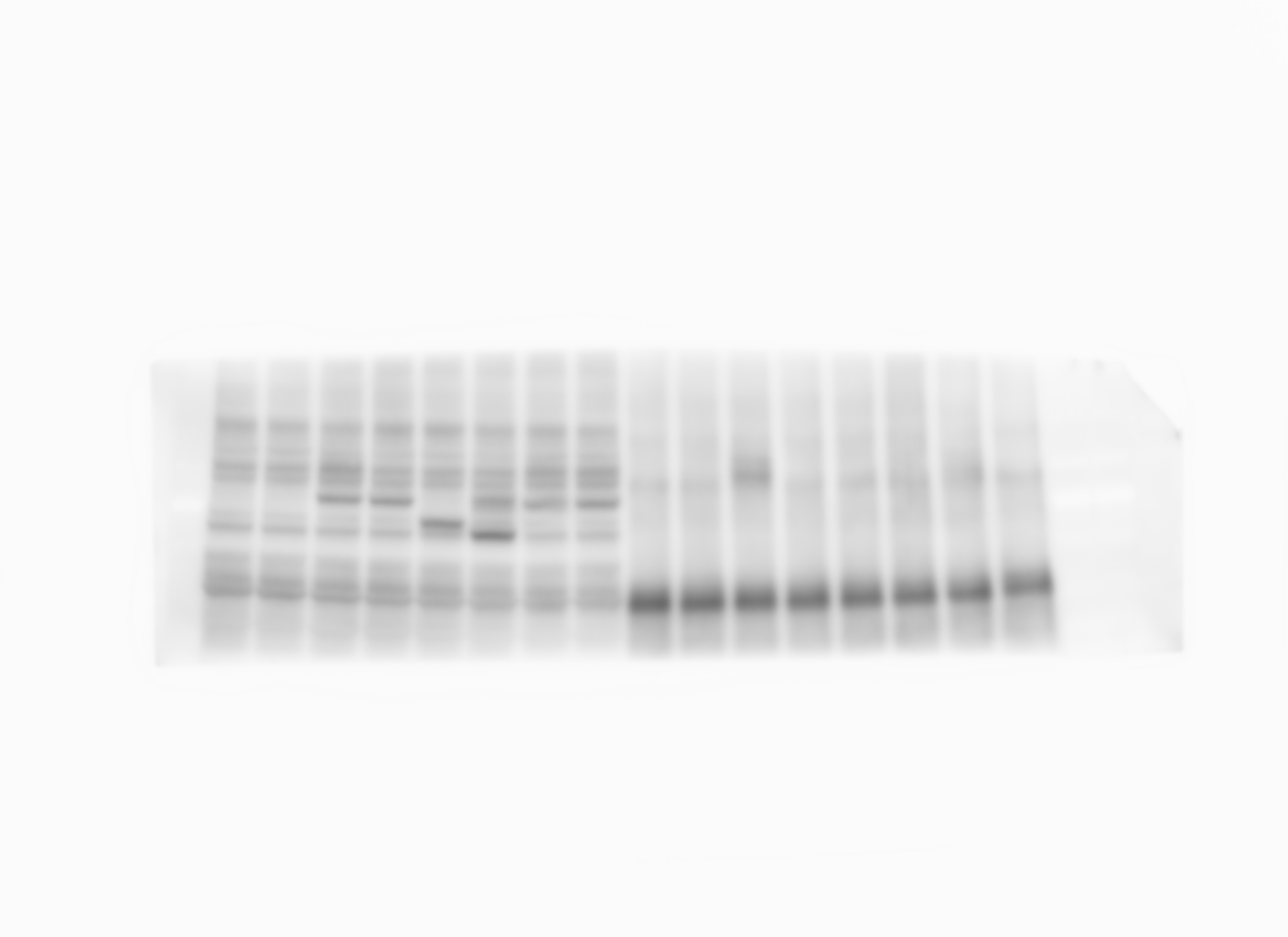

Supplement: Source data 1. [file elife-71966-data1.zip › Source Data Files/Raw Data/Figure 2-I/5, IB-Myc (Input).tif]

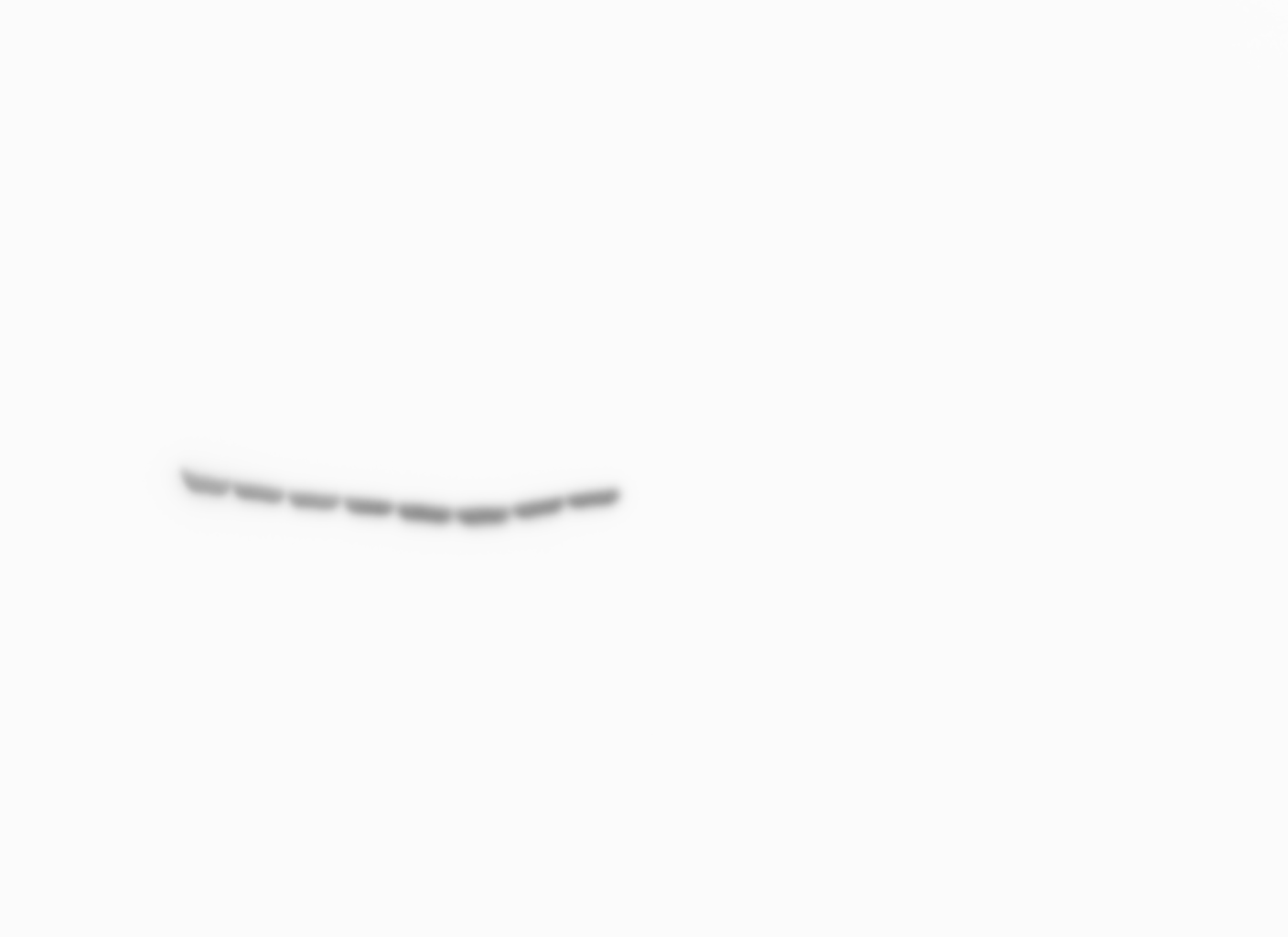

Supplement: Source data 1. [file elife-71966-data1.zip › Source Data Files/Raw Data/Figure 2-I/6, IB-Actin (Input).tif]

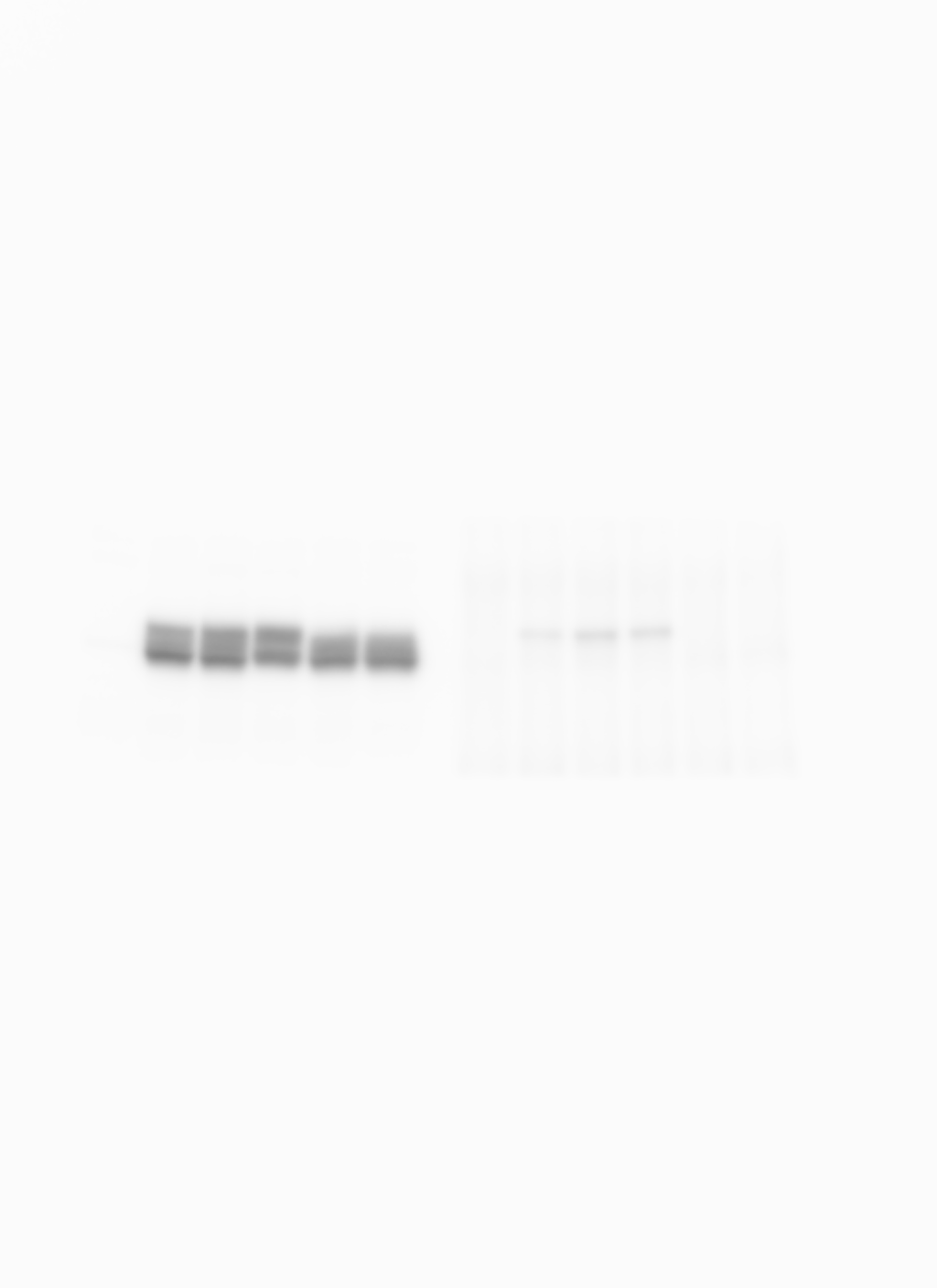

Supplement: Source data 1. [file elife-71966-data1.zip › Source Data Files/Raw Data/Figure 3-A/1 and 3, IB-HA (IP and Input).tif]

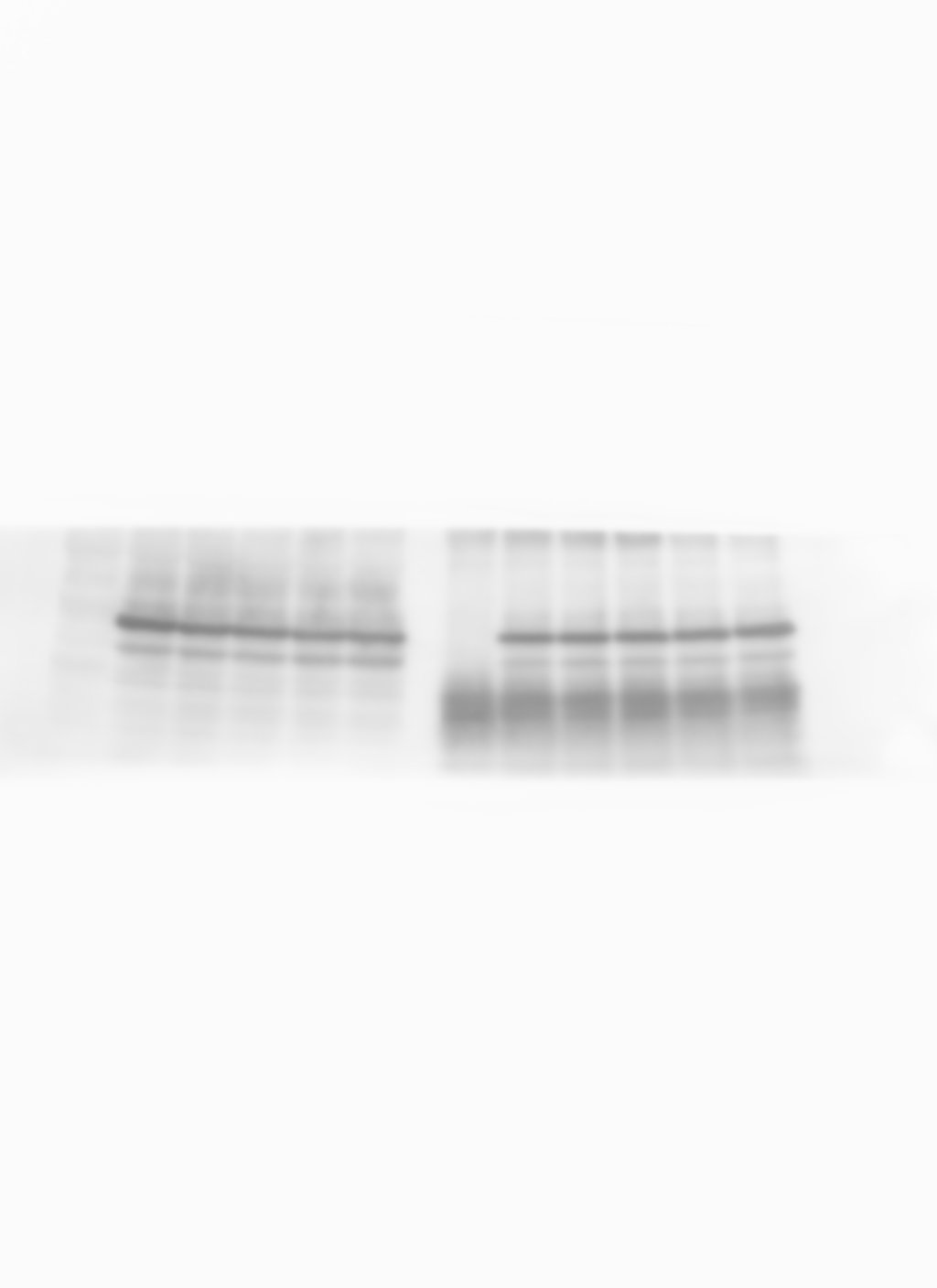

Supplement: Source data 1. [file elife-71966-data1.zip › Source Data Files/Raw Data/Figure 3-A/2 and 4, IB-Myc (IP and Input).tif]

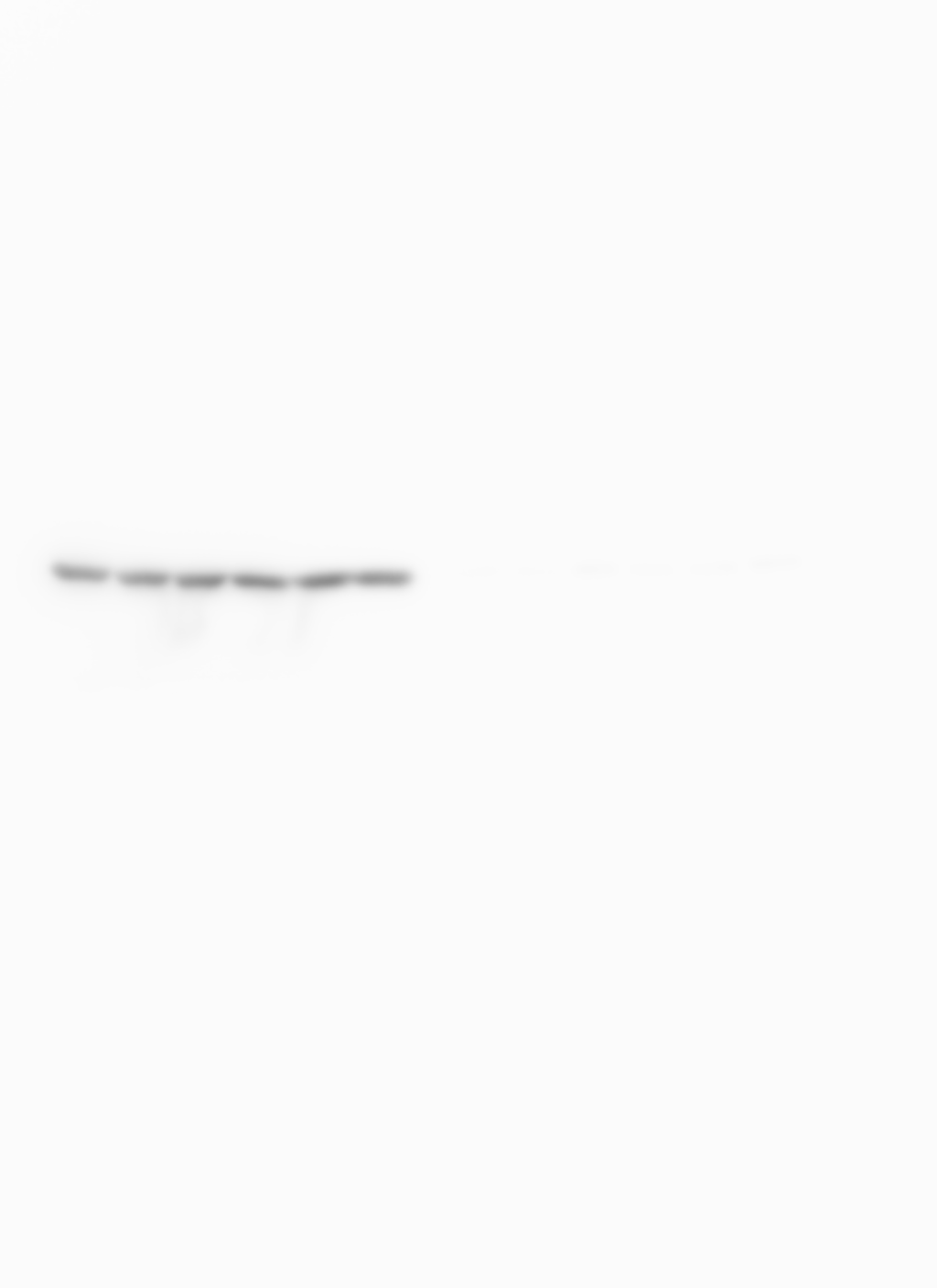

Supplement: Source data 1. [file elife-71966-data1.zip › Source Data Files/Raw Data/Figure 3-A/5, IB-Actin (Input).tif]

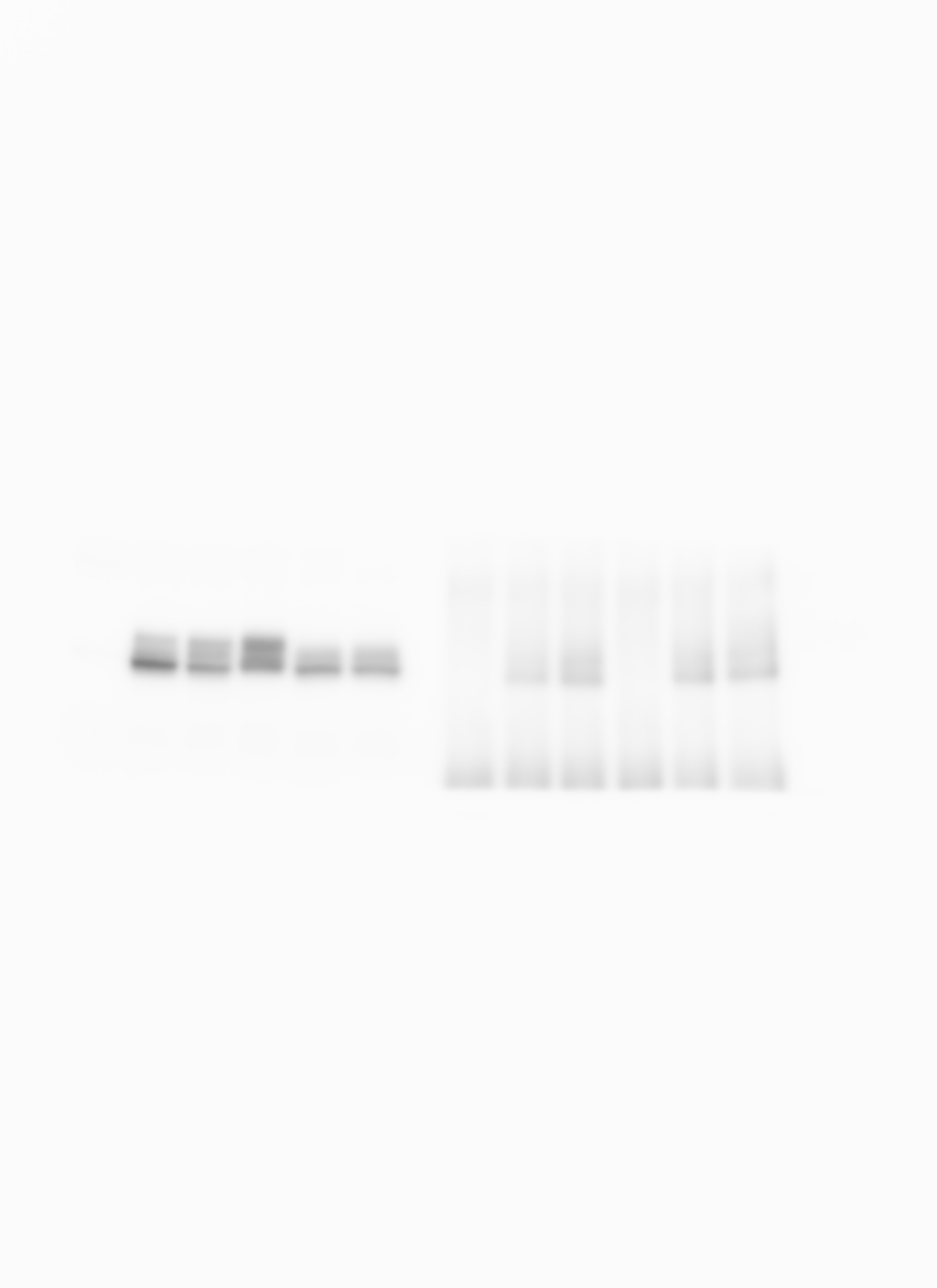

Supplement: Source data 1. [file elife-71966-data1.zip › Source Data Files/Raw Data/Figure 3-B/1 and 3, IB-HA (IP and Input).tif]

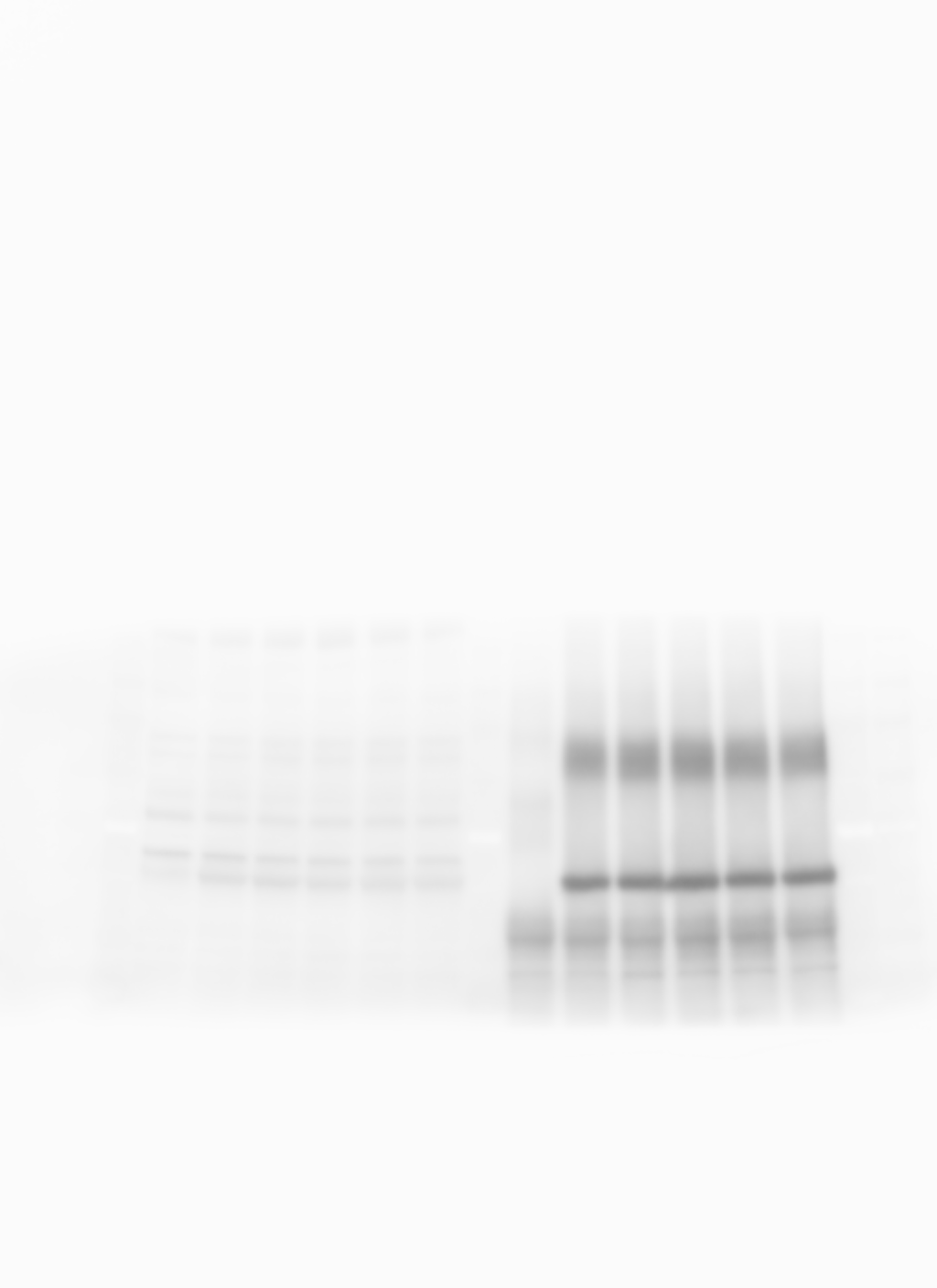

Supplement: Source data 1. [file elife-71966-data1.zip › Source Data Files/Raw Data/Figure 3-B/2 and 4, IB-FLAG (IP and Input).tif]

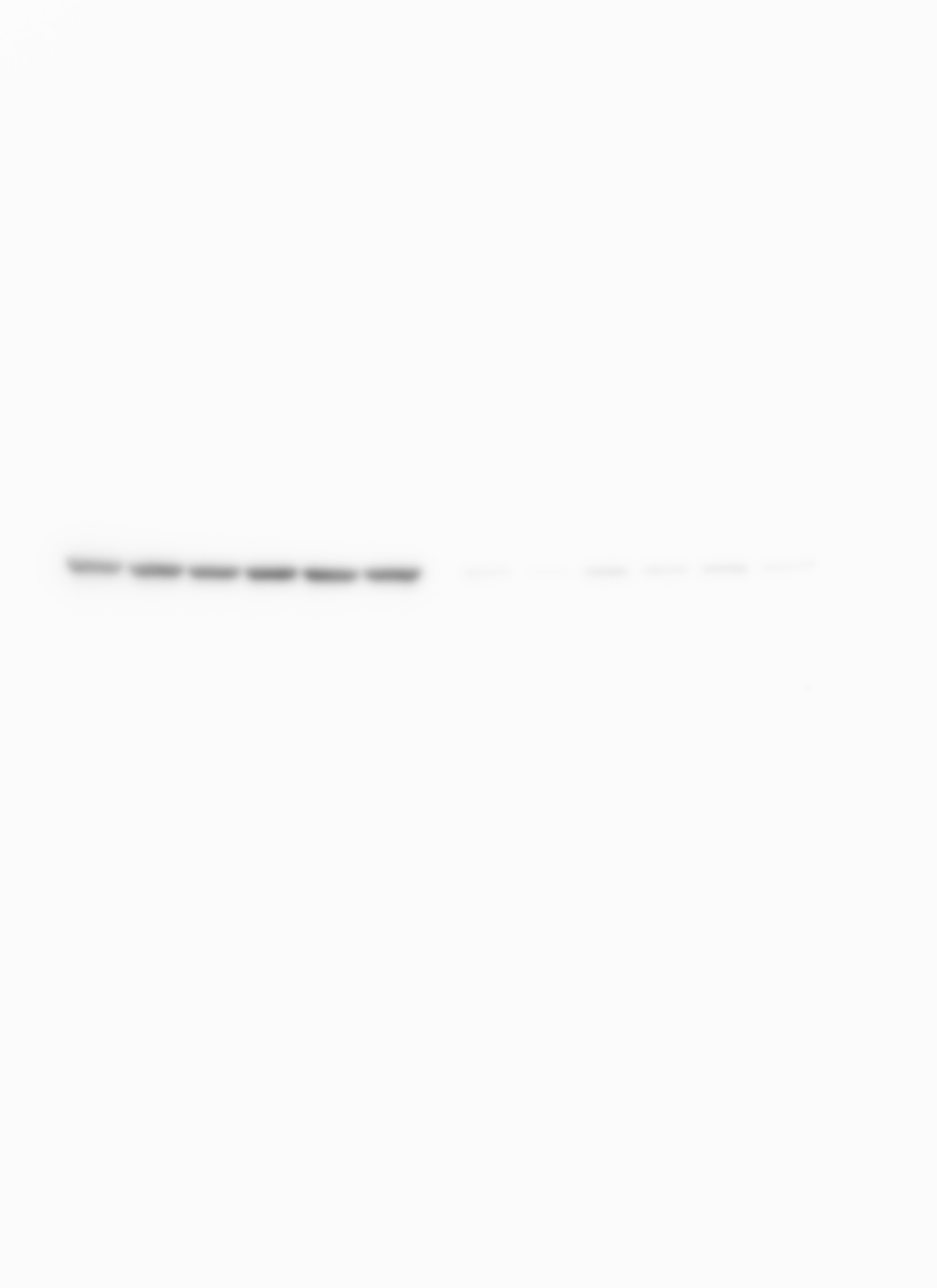

Supplement: Source data 1. [file elife-71966-data1.zip › Source Data Files/Raw Data/Figure 3-B/5, IB-Actin (Input).tif]

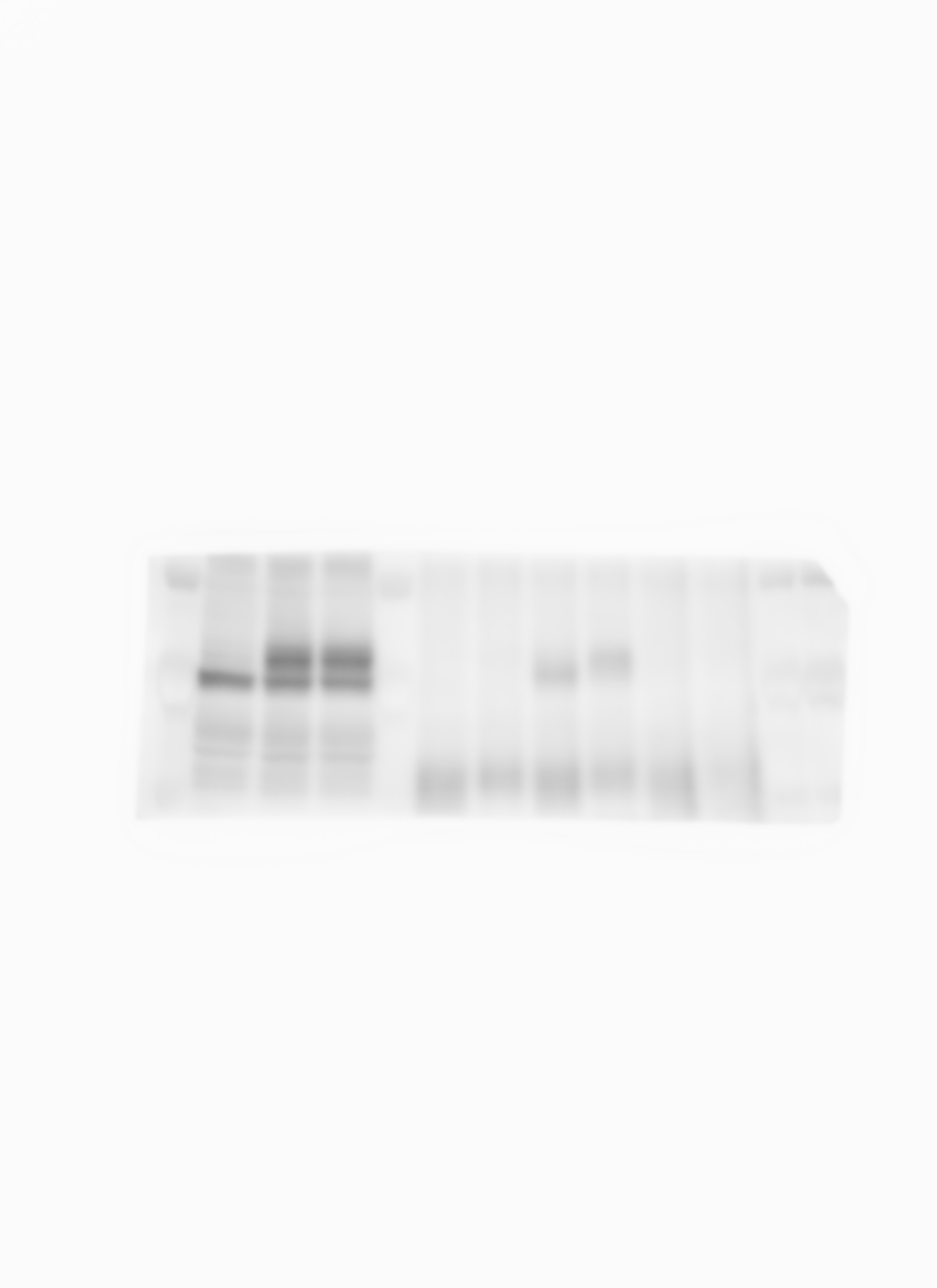

Supplement: Source data 1. [file elife-71966-data1.zip › Source Data Files/Raw Data/Figure 3-C/1, IB-Reg1.tif]

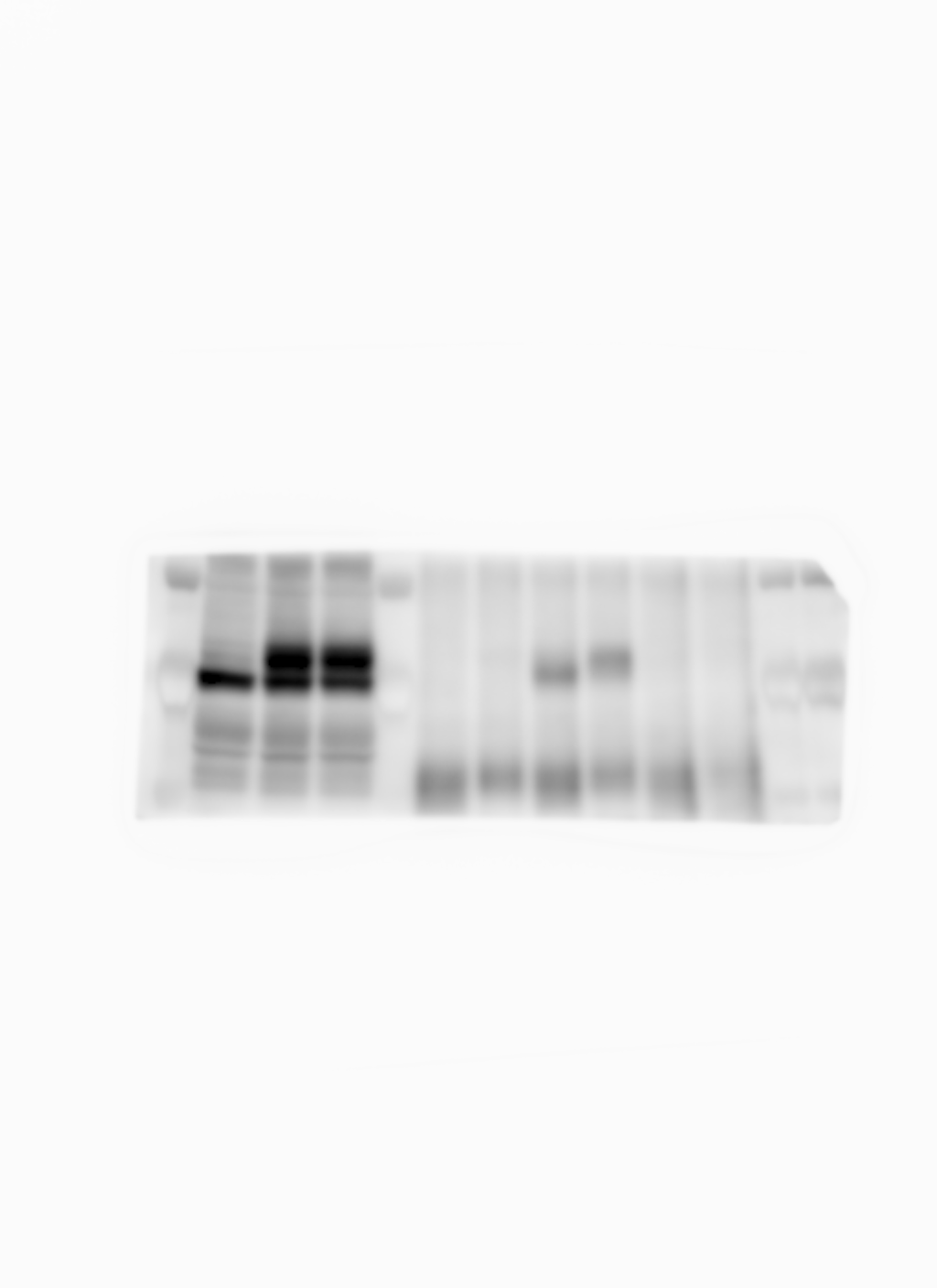

Supplement: Source data 1. [file elife-71966-data1.zip › Source Data Files/Raw Data/Figure 3-C/2, IB-Reg1 (Long exposure).tif]

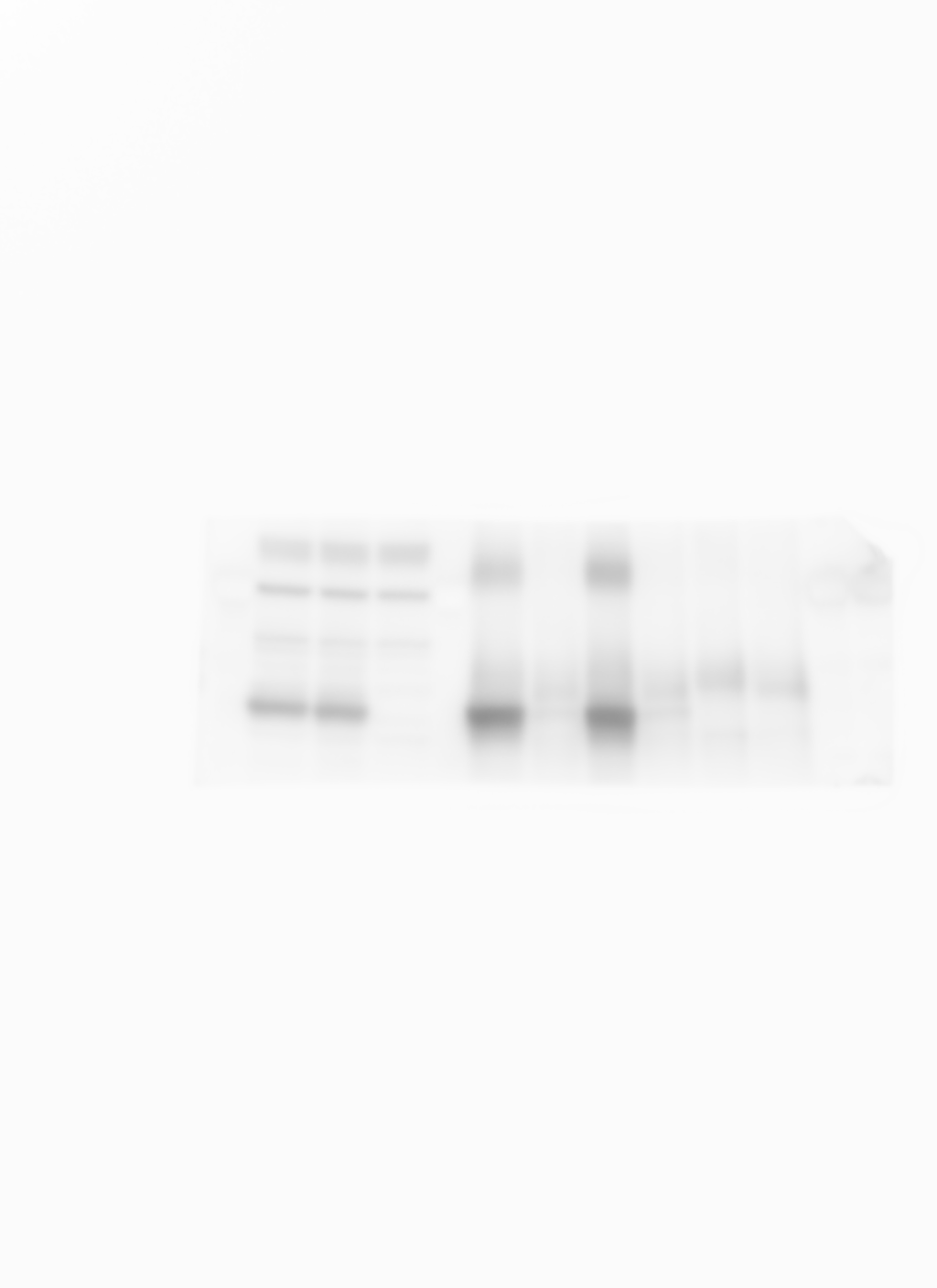

Supplement: Source data 1. [file elife-71966-data1.zip › Source Data Files/Raw Data/Figure 3-C/3, IB-FLAG.tif]

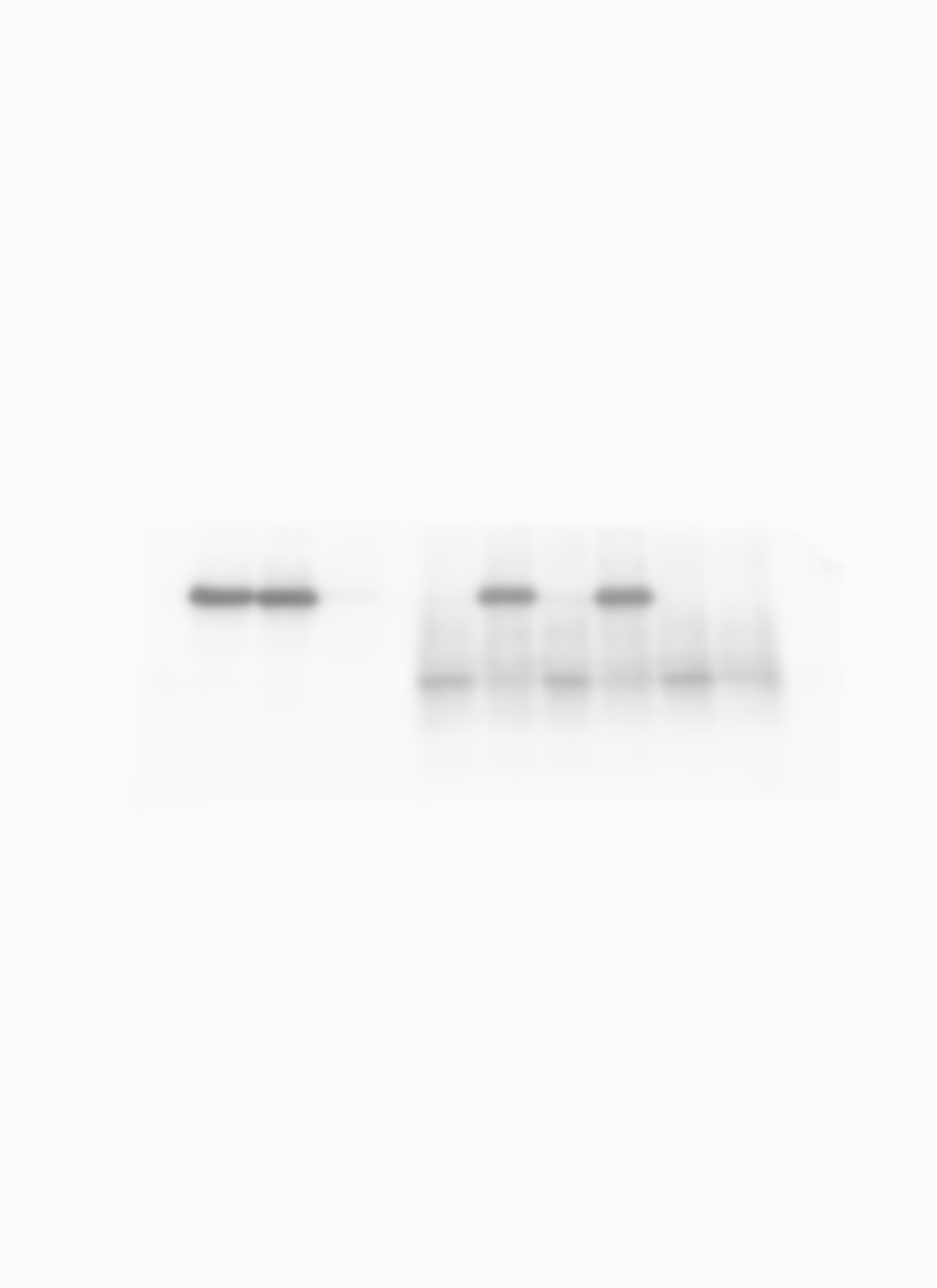

Supplement: Source data 1. [file elife-71966-data1.zip › Source Data Files/Raw Data/Figure 3-C/4, IB-HA.tif]

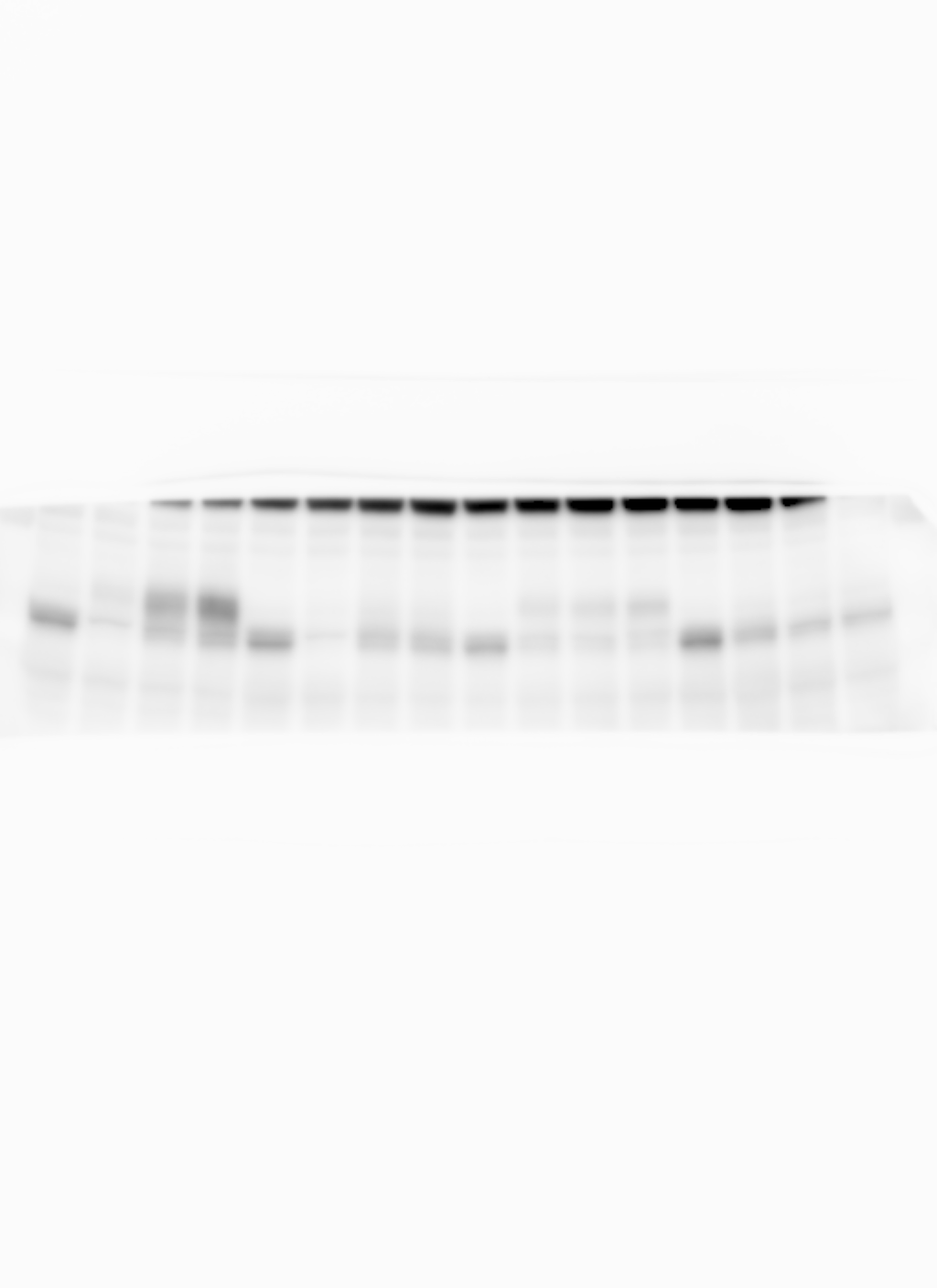

Supplement: Source data 1. [file elife-71966-data1.zip › Source Data Files/Raw Data/Figure 4-A/1, IB-Reg1.tif]

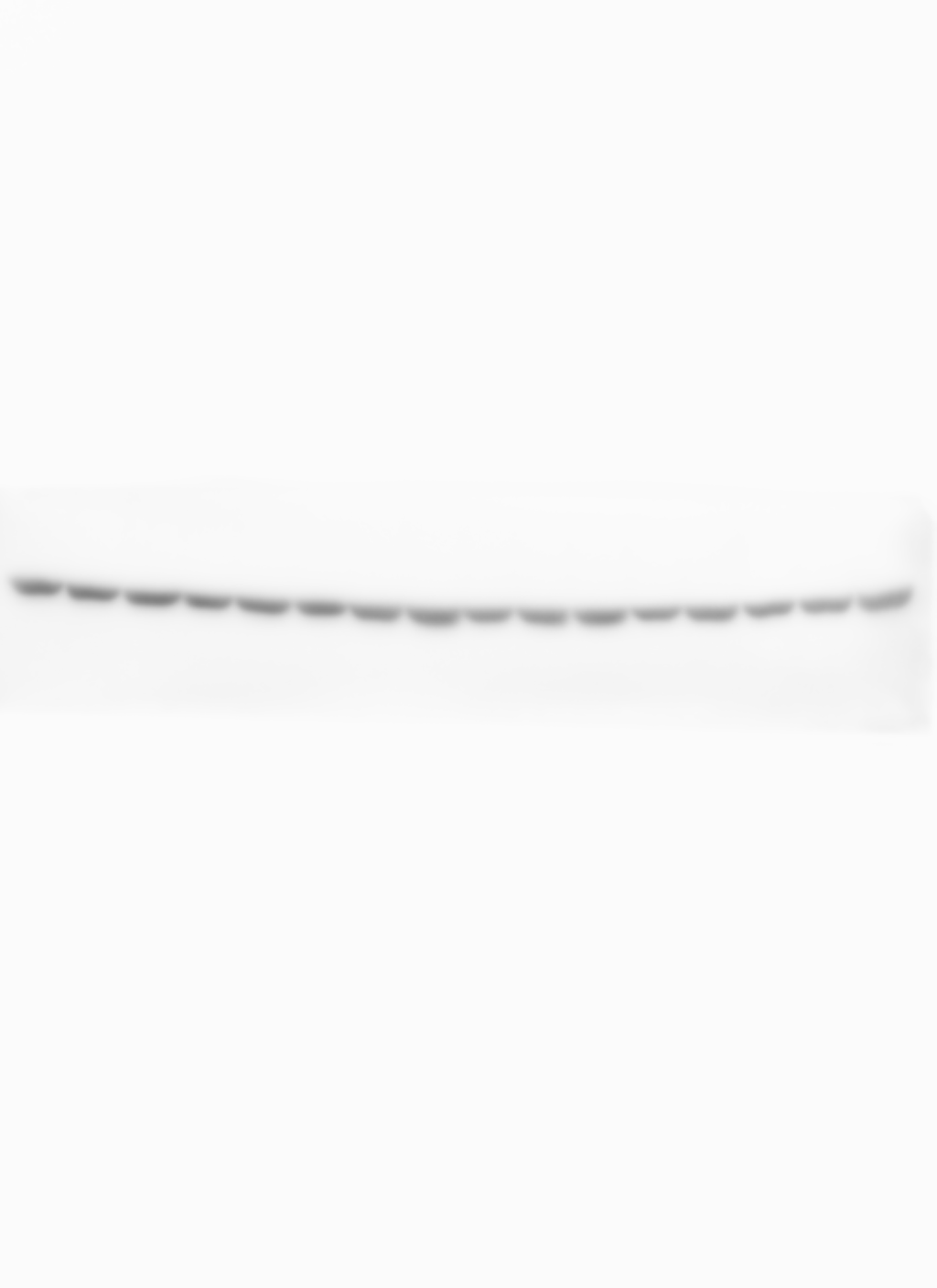

Supplement: Source data 1. [file elife-71966-data1.zip › Source Data Files/Raw Data/Figure 4-A/2, IB-Actin.tif]

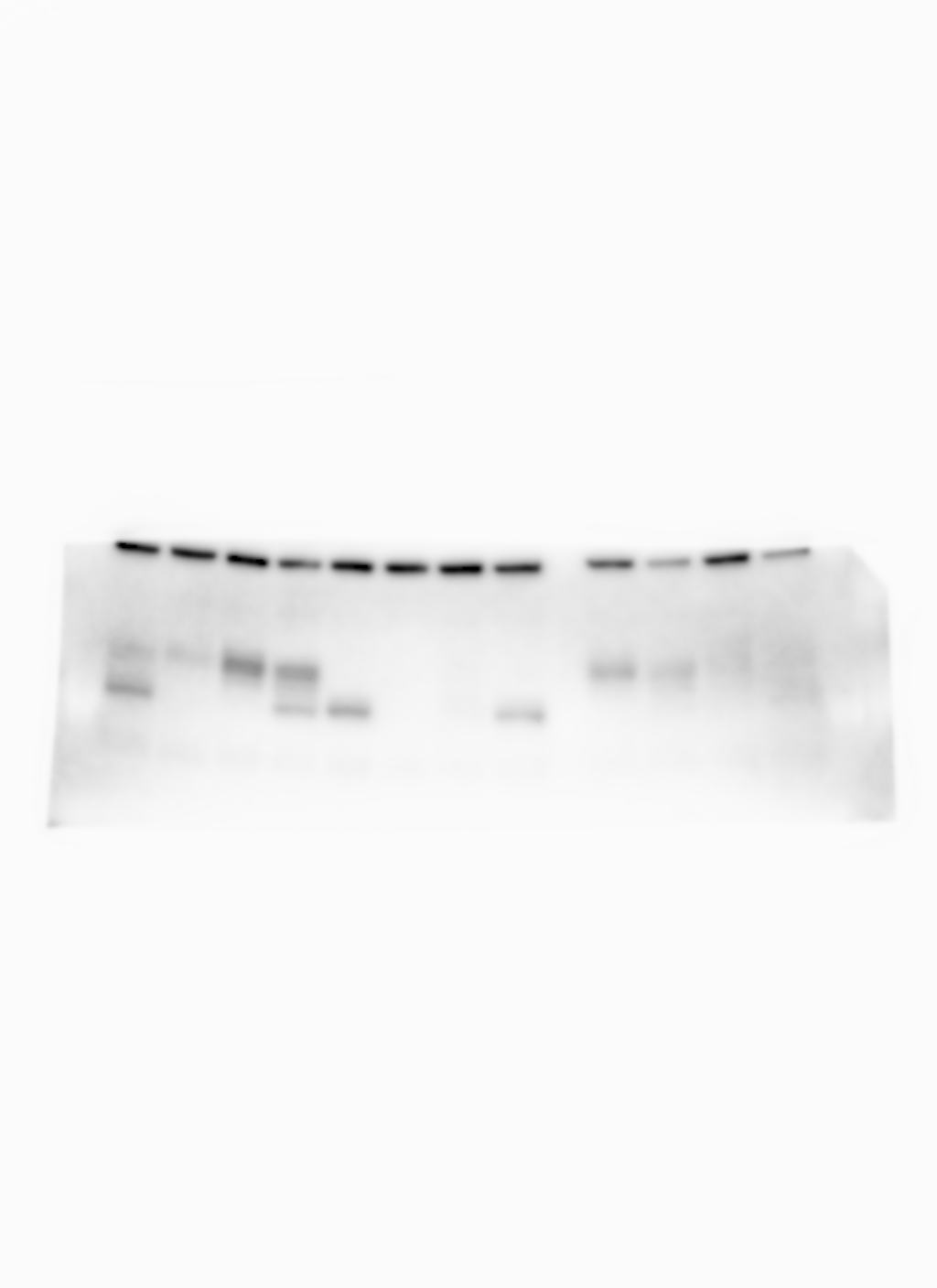

Supplement: Source data 1. [file elife-71966-data1.zip › Source Data Files/Raw Data/Figure 4-B/1, IB-Reg1.tif]

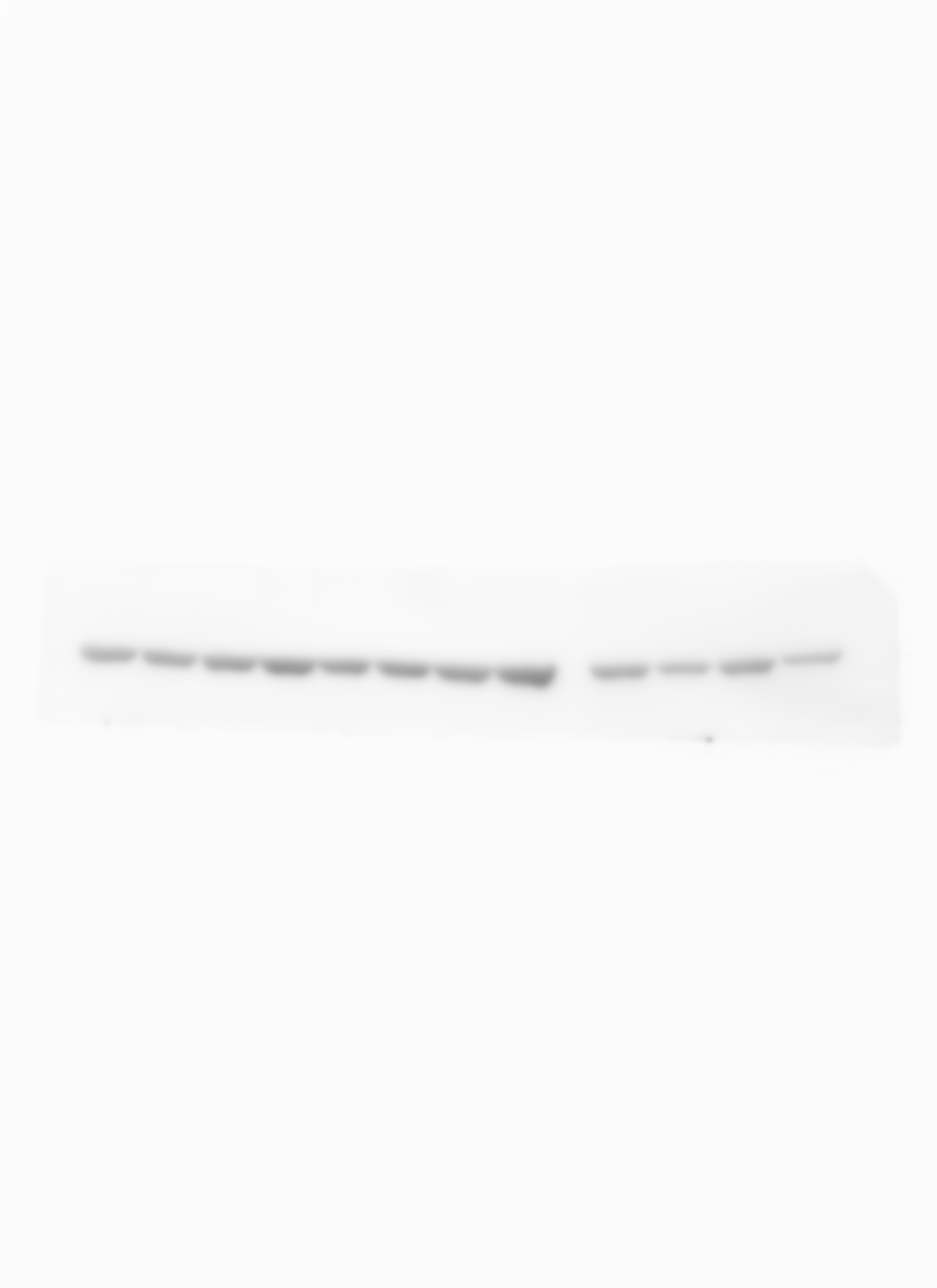

Supplement: Source data 1. [file elife-71966-data1.zip › Source Data Files/Raw Data/Figure 4-B/2, IB-Actin.tif]

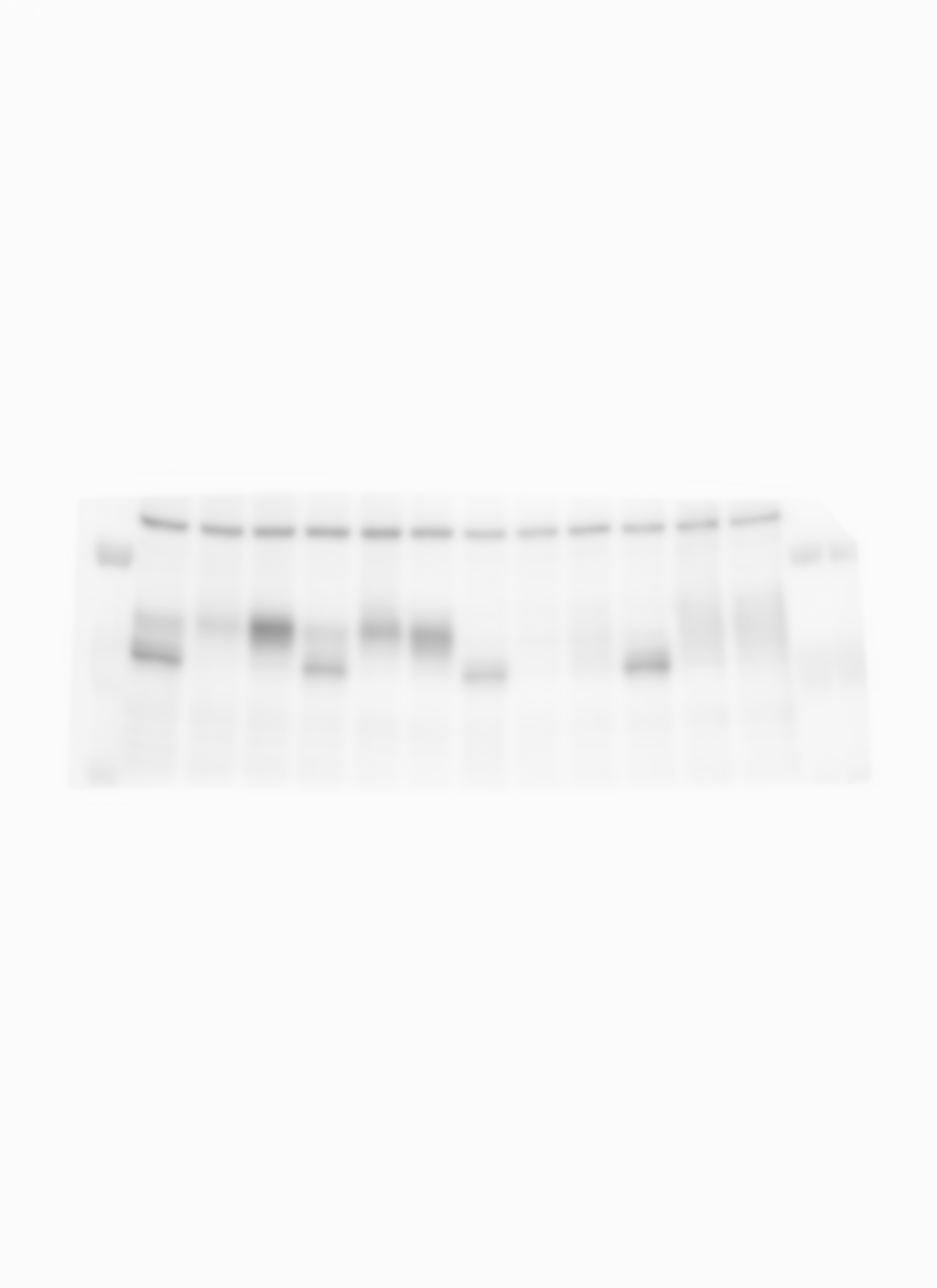

Supplement: Source data 1. [file elife-71966-data1.zip › Source Data Files/Raw Data/Figure 4-C/1, IB-Reg1.tif]

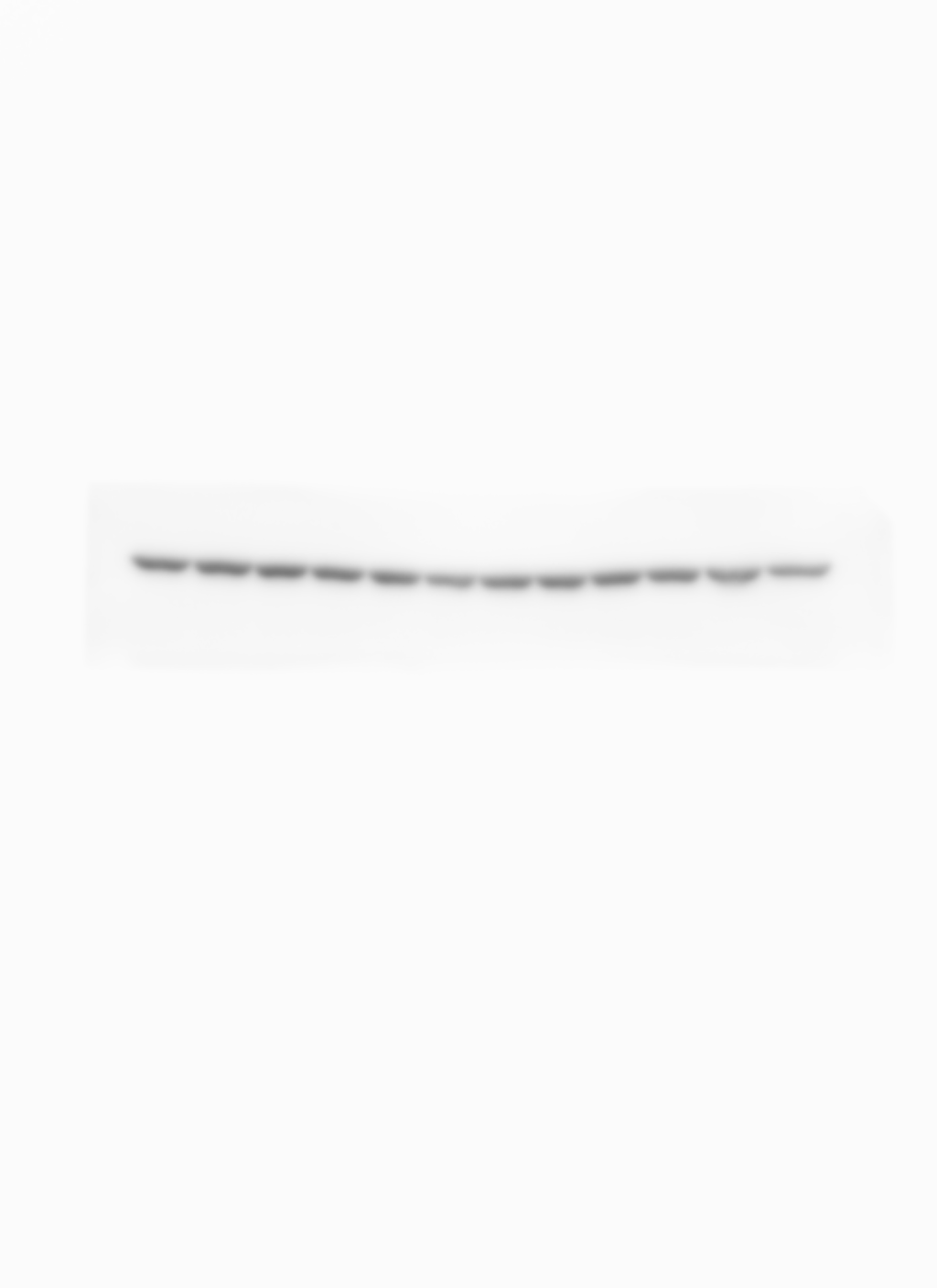

Supplement: Source data 1. [file elife-71966-data1.zip › Source Data Files/Raw Data/Figure 4-C/2, IB-Actin.tif]

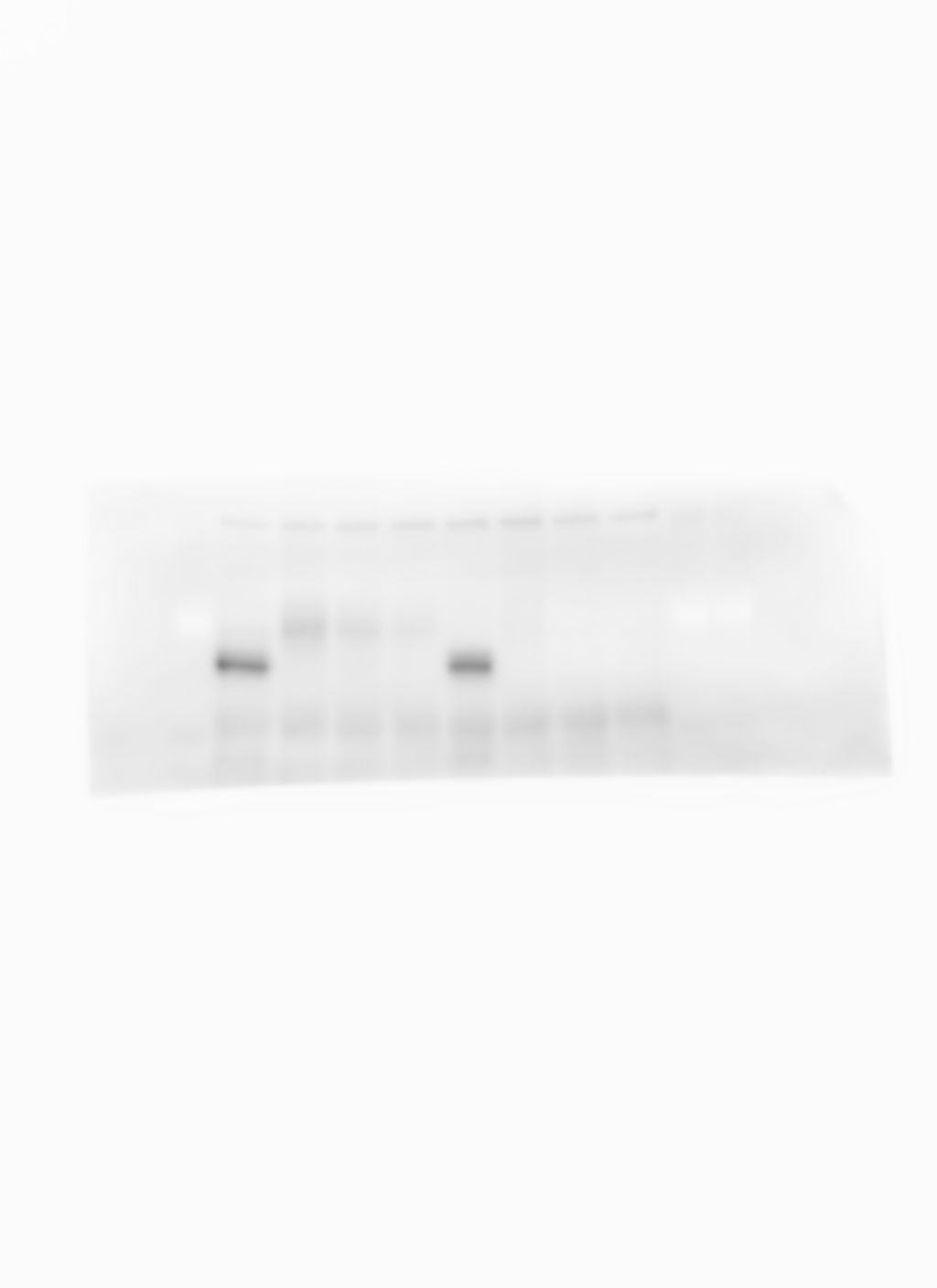

Supplement: Source data 1. [file elife-71966-data1.zip › Source Data Files/Raw Data/Figure 4-figure supplement 2/1, IB-Reg1.tif]

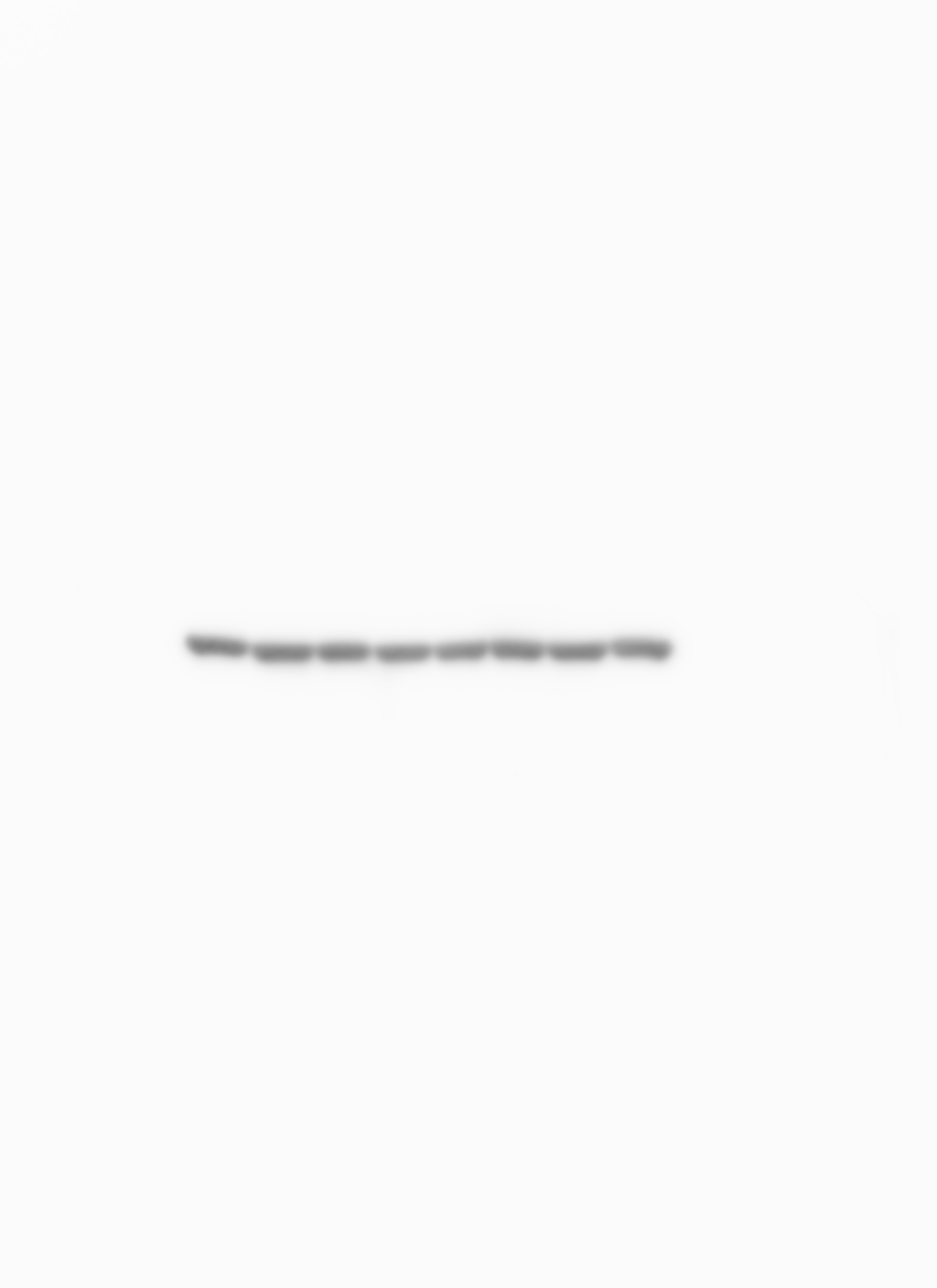

Supplement: Source data 1. [file elife-71966-data1.zip › Source Data Files/Raw Data/Figure 4-figure supplement 2/2, IB-Actin.tif]

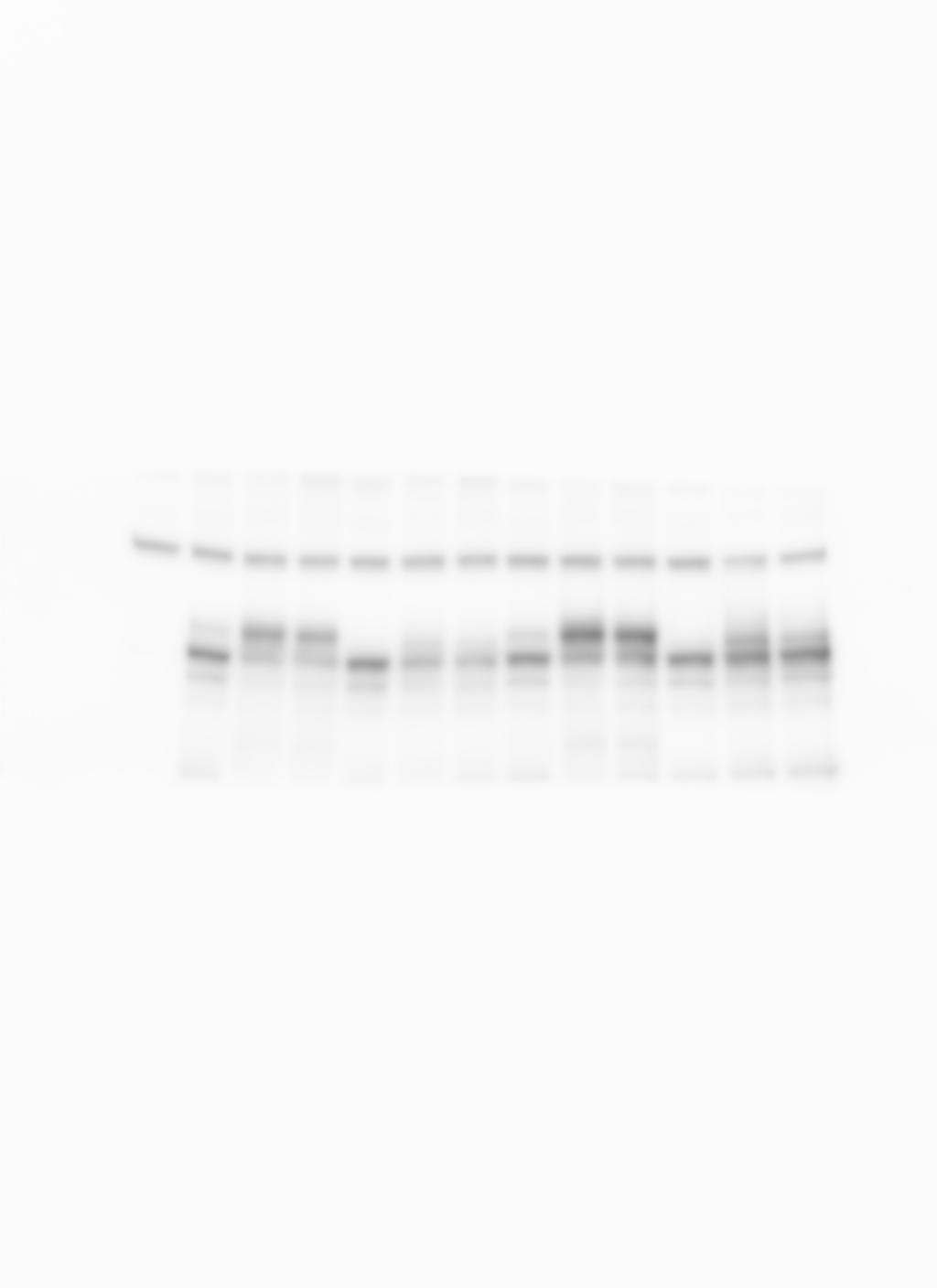

Supplement: Source data 1. [file elife-71966-data1.zip › Source Data Files/Raw Data/Figure 5-A/1, IB-Reg1.tif]

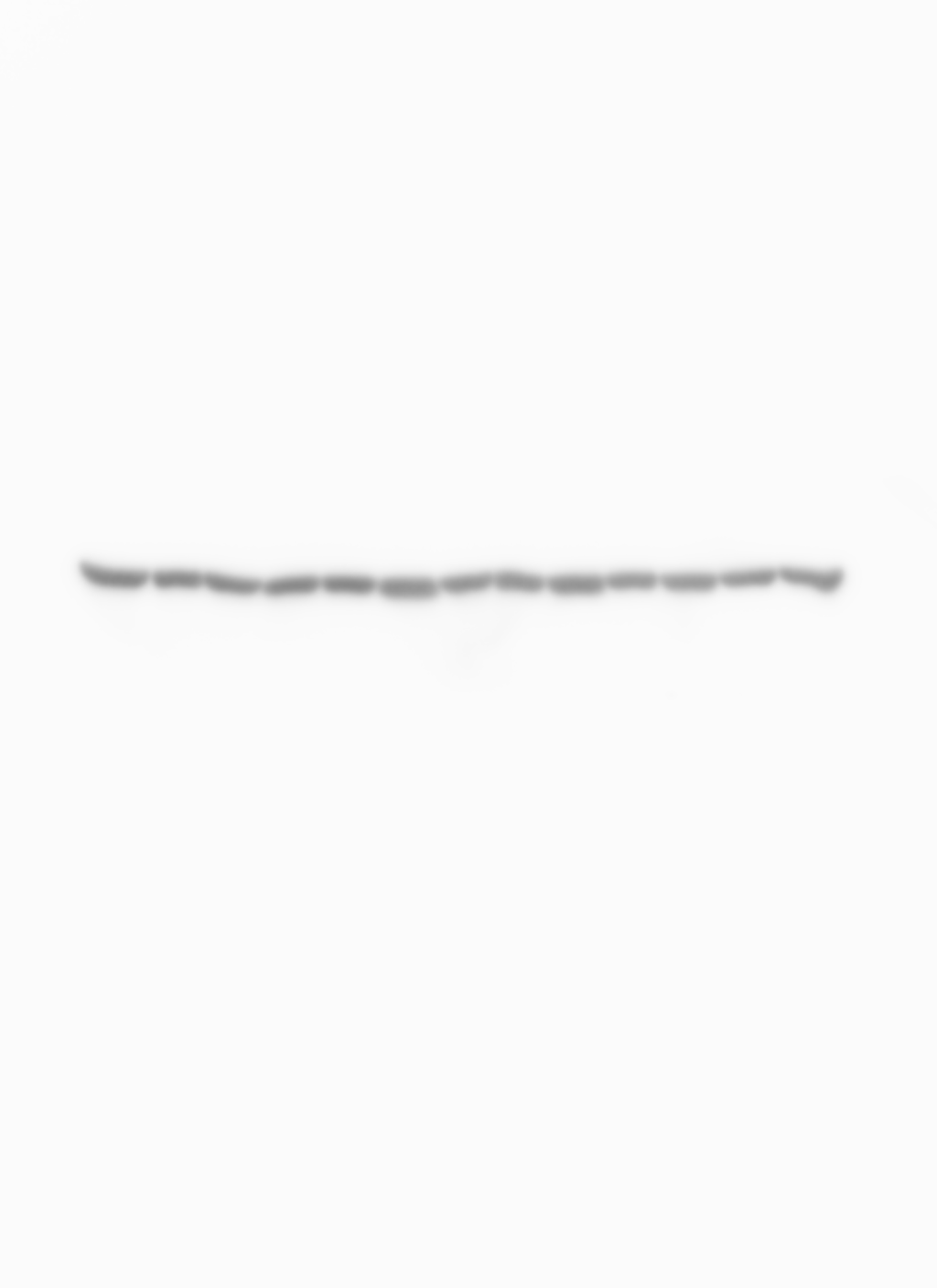

Supplement: Source data 1. [file elife-71966-data1.zip › Source Data Files/Raw Data/Figure 5-A/2, IB-Actin.tif]

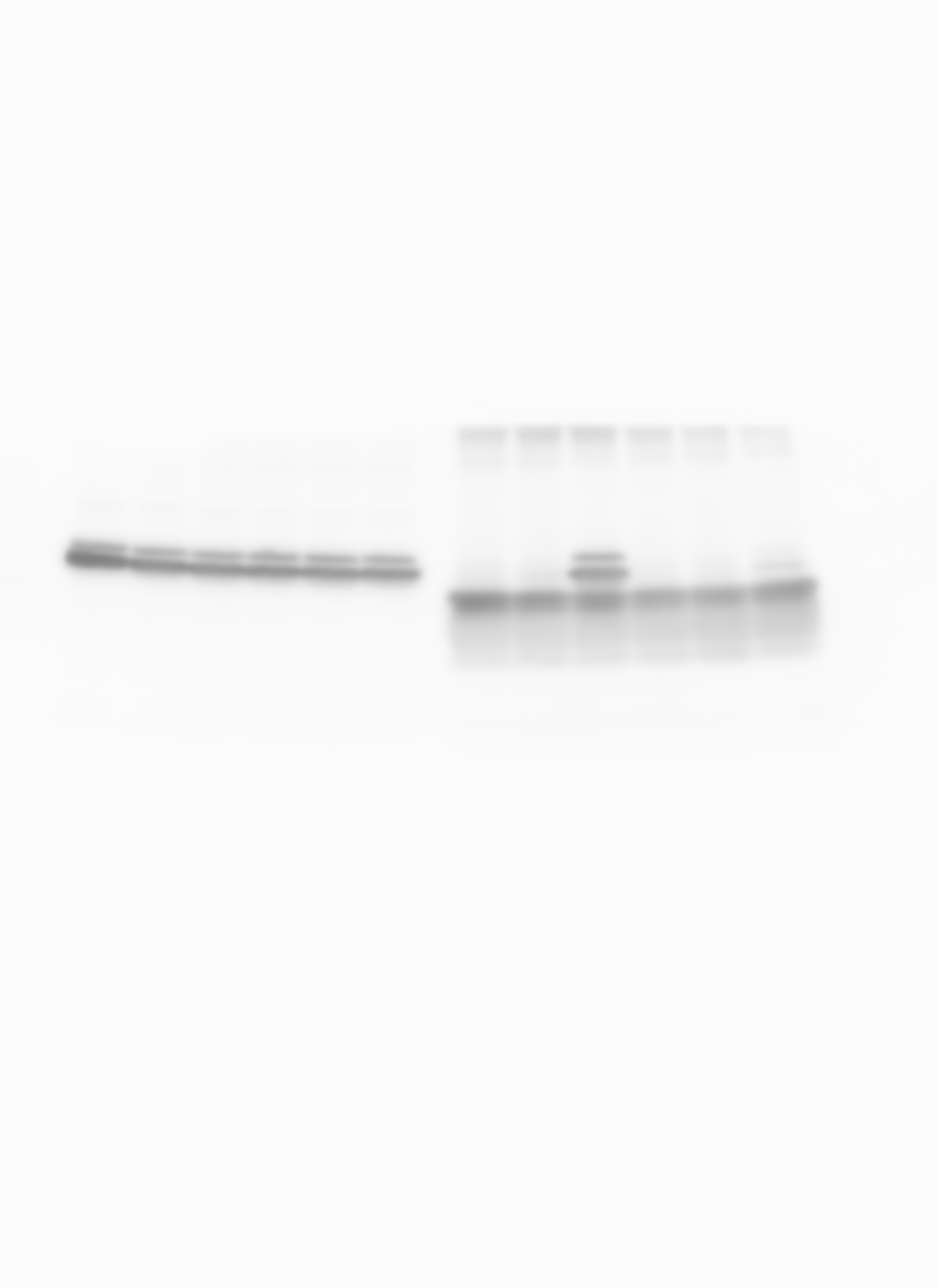

Supplement: Source data 1. [file elife-71966-data1.zip › Source Data Files/Raw Data/Figure 5-C/1 and 3, IB-pan_14-3-3 (IP and Input).tif]

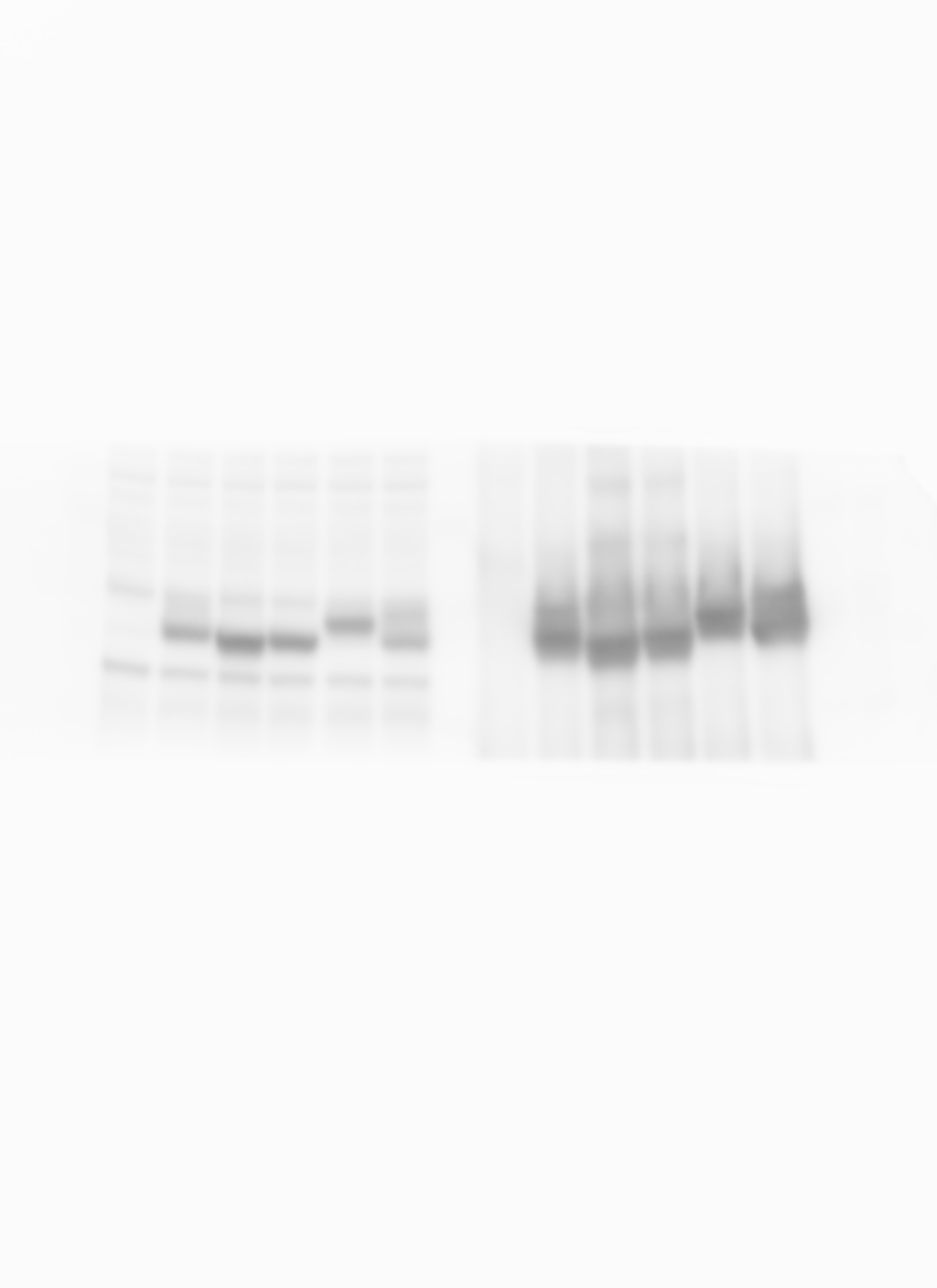

Supplement: Source data 1. [file elife-71966-data1.zip › Source Data Files/Raw Data/Figure 5-C/2 and 4, IB-FLAG (IP and Input).tif]

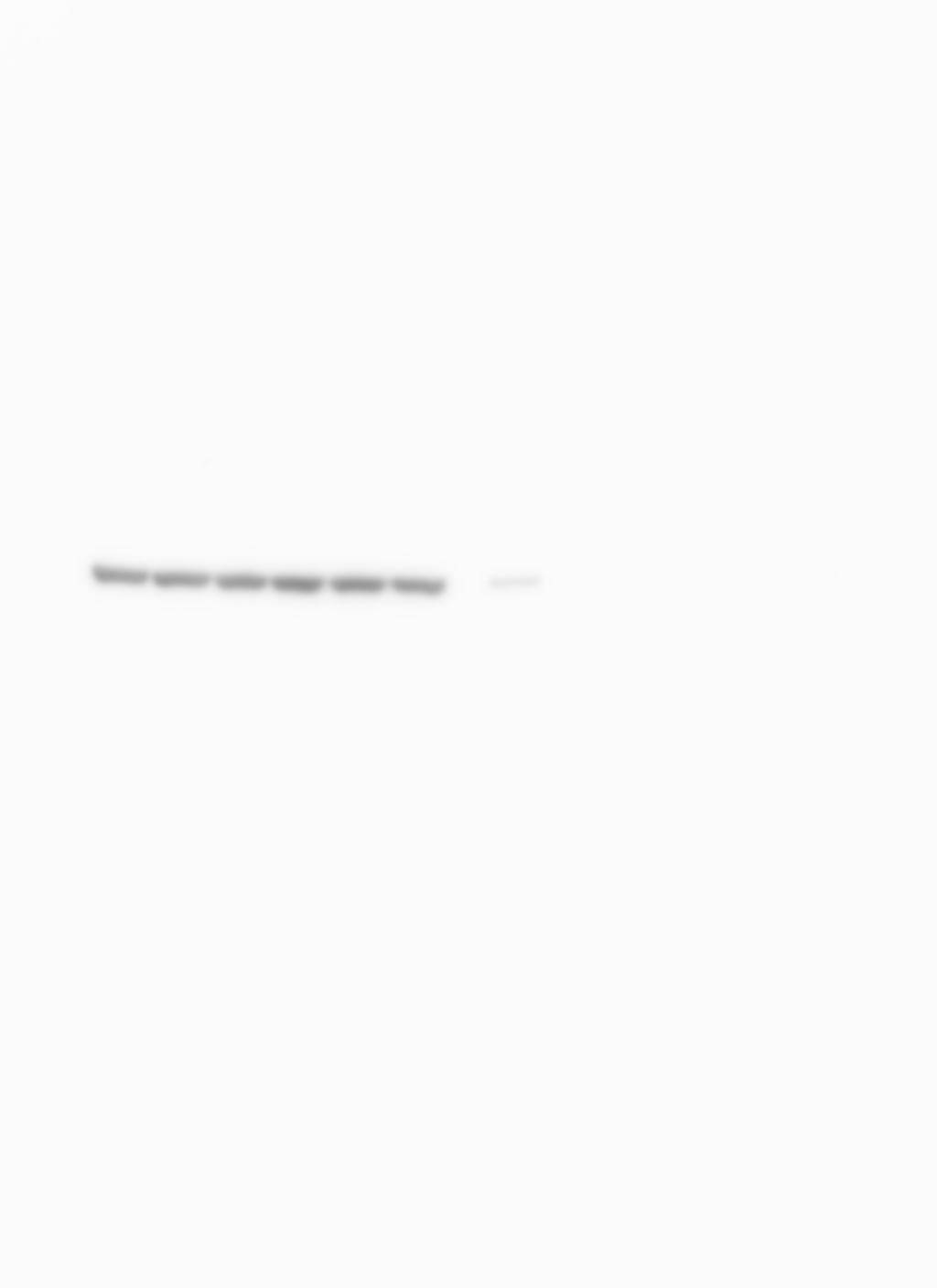

Supplement: Source data 1. [file elife-71966-data1.zip › Source Data Files/Raw Data/Figure 5-C/5, IB-Actin (input).tif]

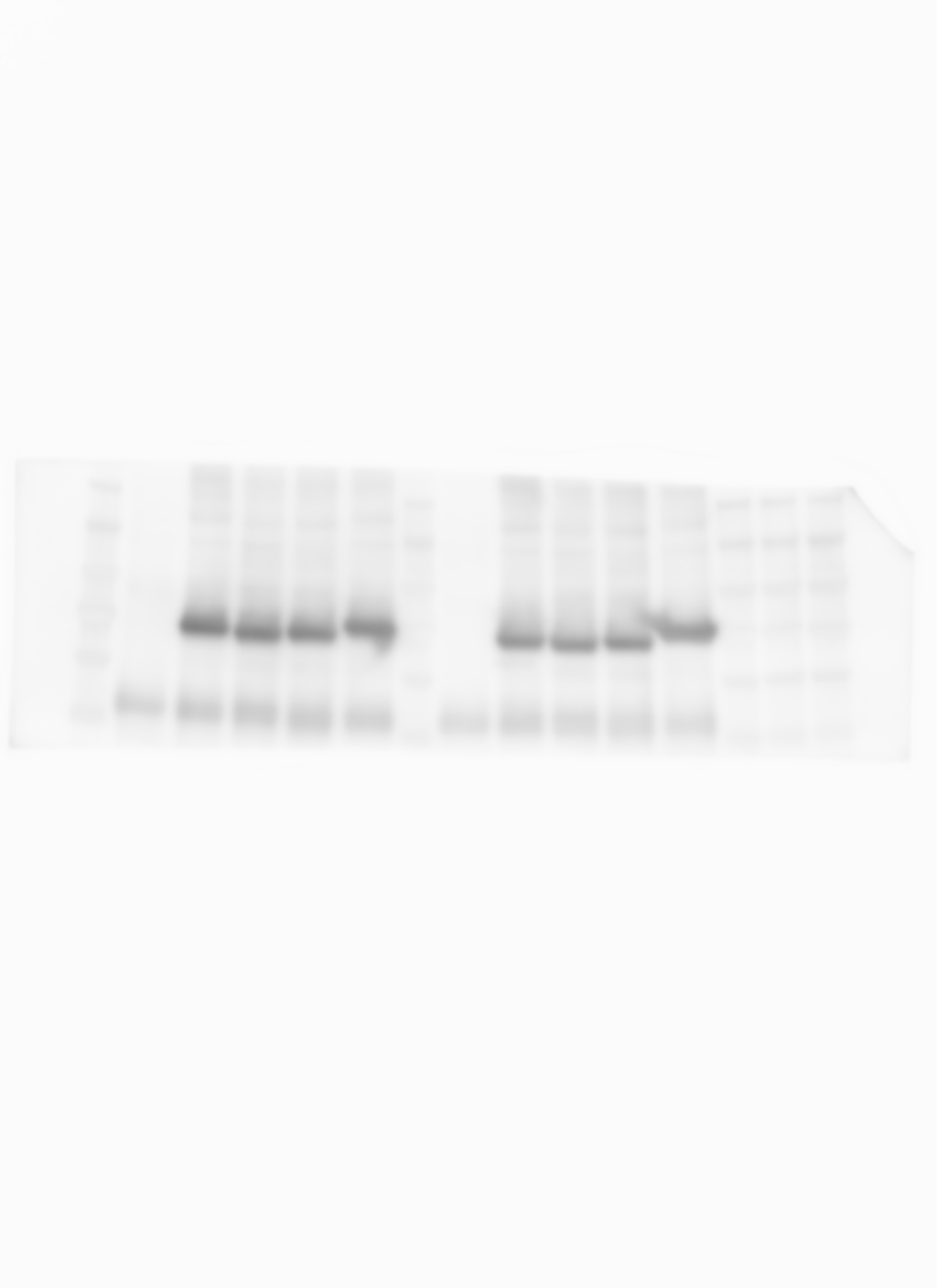

Supplement: Source data 1. [file elife-71966-data1.zip › Source Data Files/Raw Data/Figure 5-figure supplement 1/1, IB-FLAG (IP).tif]

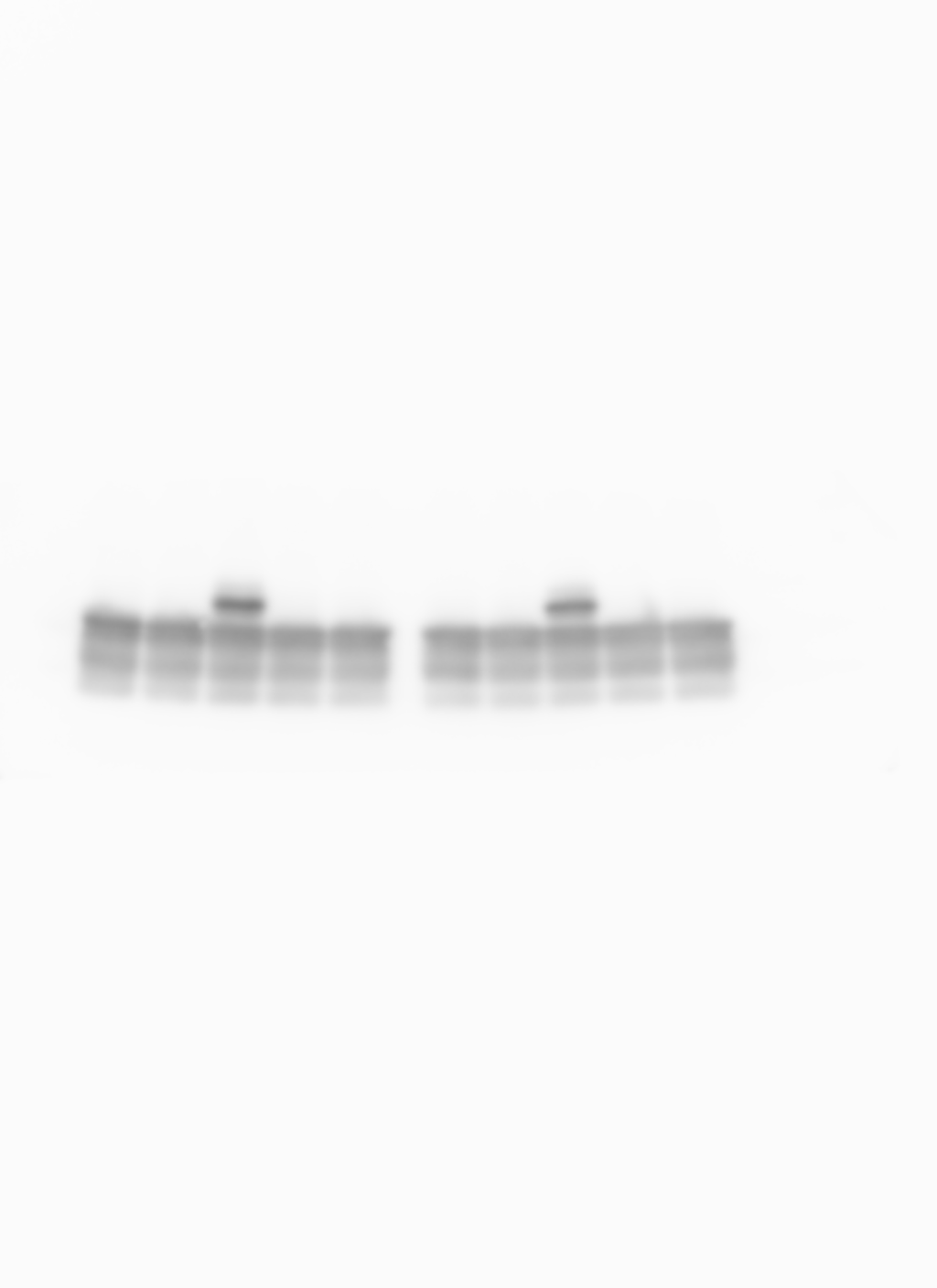

Supplement: Source data 1. [file elife-71966-data1.zip › Source Data Files/Raw Data/Figure 5-figure supplement 1/2, IB-pan_14-3-3 (IP).tif]

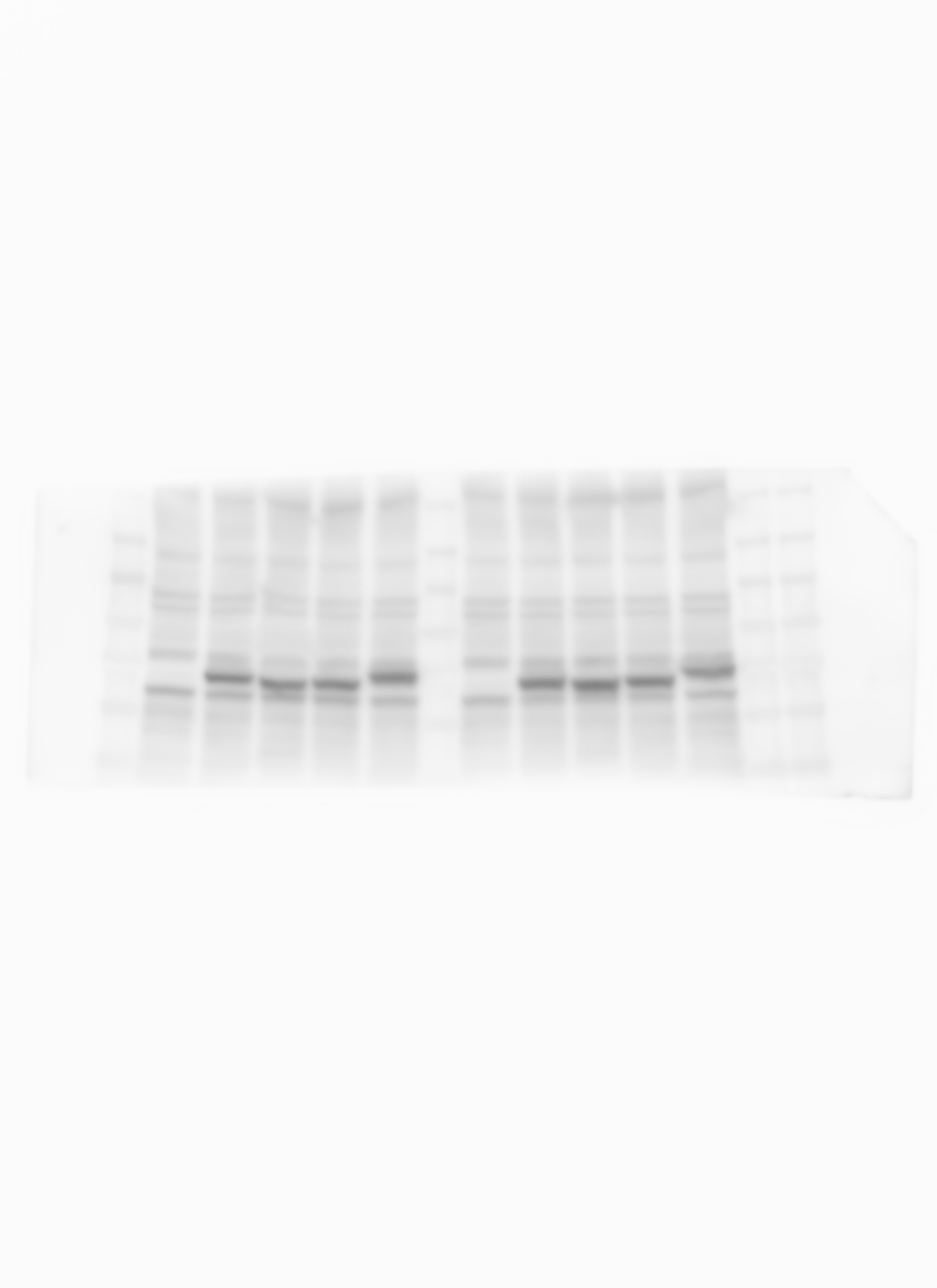

Supplement: Source data 1. [file elife-71966-data1.zip › Source Data Files/Raw Data/Figure 5-figure supplement 1/3, IB-FLAG (Input).tif]

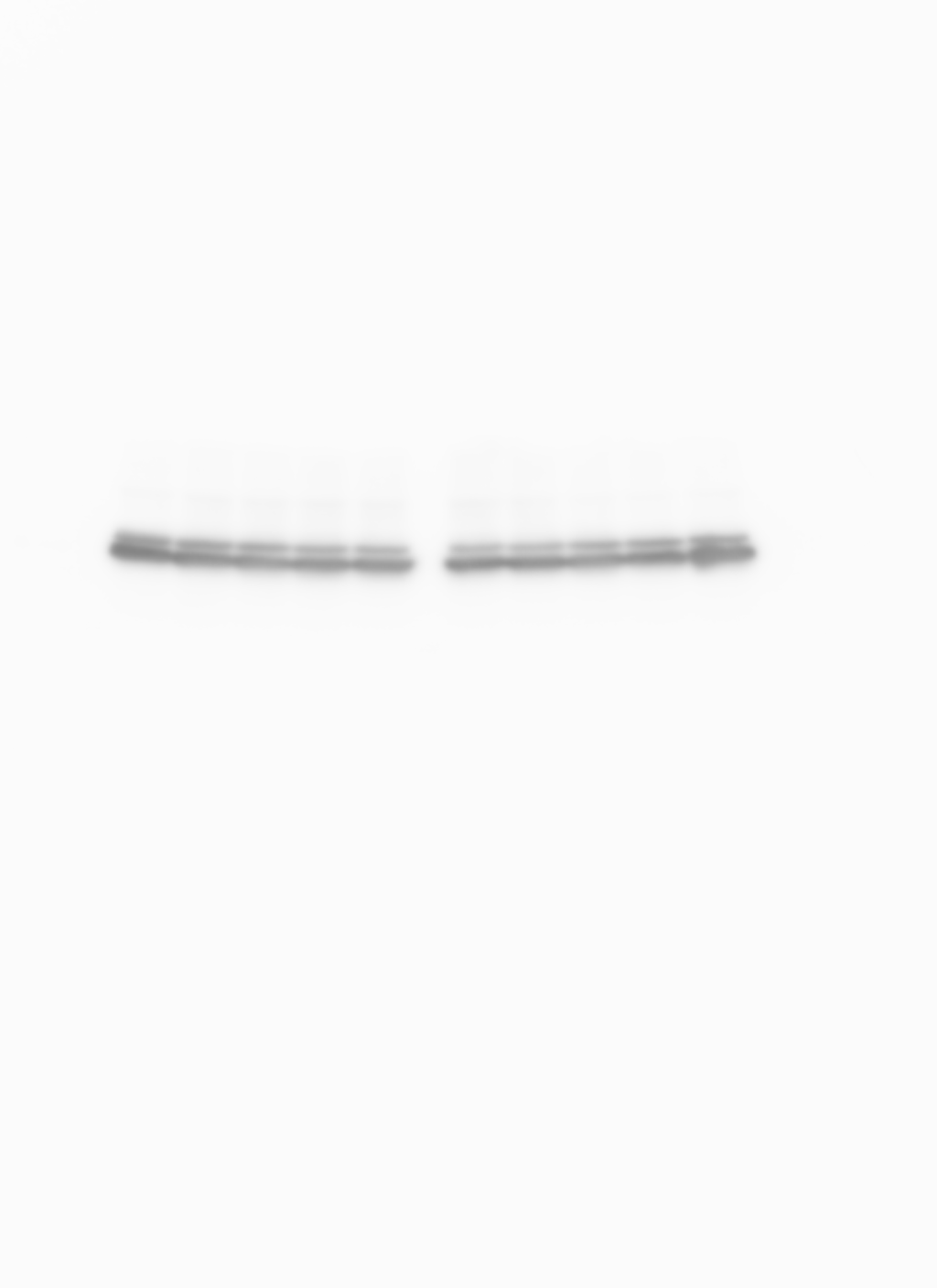

Supplement: Source data 1. [file elife-71966-data1.zip › Source Data Files/Raw Data/Figure 5-figure supplement 1/4, IB-pan_14-3-3 (Input).tif]
